# Educational Attainment at Age 10–11 Years Predicts Health Risk Behaviors and Injury Risk During Adolescence

**DOI:** 10.1016/j.jadohealth.2017.02.003

**Published:** 2017-08

**Authors:** Joanne C. Demmler, Rebecca A. Hill, Muhammad A. Rahman, Amrita Bandyopadhyay, Melanie A. Healy, Shantini Paranjothy, Simon Murphy, Adam Fletcher, Gillian Hewitt, Ann John, Ronan A. Lyons, Sinead T. Brophy

**Affiliations:** a Farr Institute, School of Medicine, Swansea University, Swansea, Wales, United Kingdom; b Institute of Primary Care and Public Health, School of Medicine, Cardiff University, Cardiff, Wales, United Kingdom; c Centre for the Development and Evaluation of Complex Interventions for Public Health Improvement (DECIPHer), School of Social Sciences, Cardiff University, Cardiff, Wales, United Kingdom

**Keywords:** Injury, Educational attainment, Adolescence, Children, Cohort

## Abstract

**Purpose:**

To examine the effect of educational attainment in primary school on later adolescent health.

**Methods:**

Education data attainments at age 7 and 11 were linked with (1) primary and secondary care injury consultation/admissions and (2) the Health Behaviour in School-aged Children survey. Cox regression was carried out to examine if attainment in primary school predicts time to injury in adolescence.

**Results:**

Pupils that achieve attainment at age 7 but not at age 11 (i.e., declining attainment over time in primary school) are more likely to have an injury during adolescence. These children are also more likely to self-report drinking in adolescence.

**Conclusions:**

Interventions aimed at children with declining attainment in primary school could help to improve adolescent health.

**Implications and Contribution**

Targeting interventions in primary school according to educational attainment might help to reduce injury risk in adolescence and improve adolescent health.

Adolescence is an important period in life that is characterized by physiological and psychological changes that can have long-term consequences for future physical and mental health [1], [2]. Once considered the healthiest stage of life, more recently there has been a shift in the age of onset of noncommunicable diseases into younger adolescent years [3].

Adolescence is a time when individuals are particularly vulnerable to injury [4]. Many of these injuries are related to the increase in health risk behaviors during adolescence, such as alcohol consumption, illicit substance misuse, and engagement in violent and other criminal behavior. Adolescence is also a time when other more ubiquitous behaviors that impact on health emerge, such as lack of physical activity, poor dietary behaviors, and cigarette smoking [1], [5], [6]. Not all adolescents will engage in risk-taking behavior, but they are more likely to do so than children and adults [7]. Some of these risk behaviors are important because they can lead to chronic diseases in later life [8] but also have more immediate consequences for rates of adolescent injuries, even after controlling for the social environment and its associated environmental hazards [9], [10].

Preventable and often self-inflicted injuries are believed to be among the greatest threats to the health and well-being of adolescents [11], [12]. Gradients in risk for injury are therefore an important influence on adolescent health inequalities [10]. Knowledge about the predictors of injury risk could be used to inform the design and targeting of preventive interventions aimed at improving adolescent health outcomes [13]. In a previous international analysis of young people, reported injuries increased in direct association with increased frequency of reported risk behaviors; this gradient remained consistent across culturally diverse countries, within all demographic strata defined by age and sex, across different injury types and with and without adjustment for potential confounders [9]. However, evidence of the effect of previous and concurrent educational attainment on adolescent injury as a proxy measure of health is sparse.

There is a well-established association between education and general health: good education predicts good health, and disparities in both are persistent and closely linked [14], [15], [16]. The influence of education on health is both potentiating and protective; it can trigger healthier futures, mitigate social stressors, and provide access to employment opportunities and life chances that could protect individuals from later-life disadvantage [17], [18]. The relationship between education and health is also mutually reinforcing: health and educational attainments affect each other [19]. Past and present states of health profoundly shape individuals' levels of educational attainment which, in turn, are consistently linked to concurrent and future states of health [15], [20], [21], [22].

Pati et al. [23] state that early school success is clearly related to later success and health [24], [25] and strongly linked to the development of child behaviors in the preteenage years [24]. Risk-taking behaviors thus acquired are known to have a major impact on health in adolescence and adulthood [26]. However, poor attainment might lead to low self-worth and negative stereotyping in students, which in turn might lead to continued low attainment and health risks [27].

This study is based on life course theory, whereby low-educated individuals are exposed to cumulative disadvantage through socioeconomic adversity, chronic stress, and poor health lifestyles and environments, among other mechanisms. All those factors manifest over the long term and culminate in poorer health in late life [28].

It was designed to contribute to this literature by examining the effect of educational attainment on adolescent health, using injury rates as a proxy for risk-taking behaviors.

## Methods

### Study design: Record-linked e-cohort study

#### Routine data

Children from the Wales Electronic Cohort for Children (WECC; version 1.4) were linked to their educational records (Pre-16 years Educational Attainment Data set), mortality data, hospital admissions data, and general practice records. WECC contains all children born in or living in Wales and registered with a general practitioner in Wales between the dates January 1, 1990 and December 31, 2013. The educational data set contains assessment results for years 2003–2012, with sparse information for earlier years. This means that only children born before 2002 (unless they took the assessment early) were old enough to be included in the analysis and only those born from 1996 onward (age 7 in 2003) had good data coverage in the education data set.

We are using two time points of educational attainment. Key stage 1 (KS1) is a national assessment in mathematics and in the English or Welsh language at age 7/8, and key stage 2 (KS2) is the equivalent national assessment at age 10/11.

The linkage and hosting of this data were through the Secure Anonymised Information Linkage (SAIL) databank [29], [30]. The SAIL databank anonymously record-links routinely collected data held in health and social care data sets at the Centre for Improvement in Population Health through E-records Research, Swansea University, United Kingdom and is part of the Farr Institute [31]. For each data set within the SAIL databank, an individual is assigned an Anonymised Linking Field, based on their names, address, or National Health Service number, which is used to link across data sets. All data within the SAIL gateway are treated in accordance with the Data Protection Act 1998.

To date, the SAIL databank incorporates over 10 billion records from multiple health and social care events and at the time of analysis received data from 42% (195/468) of general practices (GPs, i.e., these are visits to the family physician) in Wales containing information on 2,340,210 (46%) out of 5,066,916 individuals ever registered with a GP. SAIL receives all inpatient hospital episodes for Wales from the Patient Episode Database for Wales.

#### Survey data

The Health Behaviour in School-aged Children (HBSC) survey [32] is a research collaboration with the World Health Organization Regional Office for Europe and is conducted every 4 years in 44 countries across Europe and North America, asking pupils to self-report health behaviors. As part of the 2013/2014 HBSC survey in Wales, we conducted an Medical Research Council–funded pilot study among adolescents aged 11–16 years in nine secondary schools, who were asked to complete the HBSC questionnaire and to consent to linkage of their survey data to their health and education data within the SAIL databank.

### Analysis

#### Routine data

Data linkage and data preparation within the SAIL databank were conducted using IBM DB2 9.7 SQL. Data were then imported into Stata 13, which was used for all statistical analyses.

The aim of this study was to compare injury rates among children that have the same education attainment at baseline (attainment at age 7), that is, they either attain at age 7 (group A) or they do not (group B). Children in the same group of analysis should therefore have the same potential for learning and achieving at age 11. We hypothesized that not achieving in the early years had a detrimental impact on health behavior in adolescence. We used injuries during adolescence as a proxy for health behavior to assess the impact; however, 15 children left Wales before the age of 12 years and were therefore removed from the cohort. Reasons as to why children might be missing are rather complex: (1) obviously we only have good data coverage for the years 1996–2001; (2) children move in and out of Wales (or die) and might leave the school before the attainment assessment or only join the school afterwards; and (3) children might take the assessment early or late which means their attainment was outside of our data coverage. Other reasons for missing attainment might be illness or repeat of school years.

Confounders were calculated at baseline (KS1; see Supplement 1). The KS1 assessment date was set to the first of May of the assessment year. The mean age at KS1 was 7.18 years. If the assessment year was missing (.1% of cases) then it was set to the assessment date following the child's seventh birthday.

The final cohort of children was stratified according to achievement at KS1, that is, group A: achieved KS1 and group B: did not achieve KS1. The groups were stratified further by achievement at KS2 and compared for time to injury after age 12 years. Therefore, analysis of group A examined those who had achieved all core indicators (mathematics and languages) at KS1 and compared those who achieved KS2 (consistent achievers) and those who did not achieve all core indictors at KS2 (declining in attainment) for injury outcomes in adolescence. Analysis of group B examined injury outcomes for those who had not achieved KS1 and compared those who also did not achieve core indicators in KS2 (consistent nonachievers) with those who did achieve in KS2 (improving in attainment). For comparison, we added a further analysis C, which examined those improving in attainment (did not achieve KS1 but did achieve KS2) with those declining in attainment (did achieve KS1 but did not achieve KS2).

Outcomes assessed were time to admission for injury between the age of 12 and 18 years (i.e., during secondary school) in the inpatients data set and contacts to the GP for injury. Analysis was conducted for boys and girls separately.

Cox regression analysis was used for analyses of groups A, B, and C. The follow-up was calculated as the time from the child's 12th birthday to outcome assessed as the date of (1) death, (2) injury, (3) migration out of Wales, (4) end of study, or (5) the child's 18th birthday. Log-log survival plots and Schoenfeld residuals were used to assess the assumption of proportionality of hazards. The analyses were repeated to adjust for free school meal entitlement/deprivation characteristics.

Outcomes of injury were defined using medical records. GP data in the United Kingdom are coded using Read Codes which contains some 300,000 codes for symptoms, diagnosis, treatment, and management [33]. Data within the hospital admission system are recorded using International Classification of Diseases-10 codes [34] (see Supplement 2 and 3).

#### Survey data

Chi-squared analysis was used in Stata 13 to examine the proportion of pupils self-reporting alcohol consumption and alcohol intoxication according to educational achievement in primary school. The questions and variables this analysis was based on can be found in Supplement 4.

### Ethical approval

The study design uses anonymized data, and therefore, the need for ethical approval and participant consent was waived by the approving institutional review board. The independent Information Governance Review Panel, which contains members from the UK National Health Service Research Ethics Service, approved the study. The HBSC survey data aspect of the study required participant consent to link survey data to health and education records. Ethical approval was obtained from the Cardiff University School of Social Sciences Research Ethics Committee and the Information Governance Review Panel.

## Results

A total of 172,436 children (male: 88,384; female: 84,052) had assessment results for both of the national assessments at age 7 and 11.

Fifteen children were removed from the cohort as they had either died or left Wales before their 12th birthday (providing no follow-up data), leaving 172,421 children in the cohort. After stratification by KS1 results there were Analysis A: 126,240 (73%) children who achieved both KS1 and KS2 (achievers), and 13,396 (7.8%) who achieved KS1 but not KS2 (declining), Analysis B: 9,858 (5.7%) children who did not achieve KS1 but did achieve KS2 (improvers), and 22,927 (13.3%) who did not achieve either of the national assessments (nonachieving; see Supplement 5).

### Primary care

Nearly 99.99% of the children with educational attainment data were registered with a general practitioner. Thirteen thousand four hundred thirty-four children had a GP consultation because of injuries (7.8% of the joint WECC and educational attainment cohort). Only 14 children could not be linked to GP data. The time to the first injury-related GP contact was analyzed using Cox regression. Twenty children had a follow-up of less than 1 day (i.e., injury on the day they left the cohort) and were excluded from analysis, leading to 172,401 children in the primary care cohort. The mean time of follow-up from the 12th birthday to first injury-related GP contact was 2.45 years. The full results of this analysis including confidence intervals can be found in Table 1.

**Table 1:** Injury rate; time to first GP contact for injury by key stage achievement and gender

	Number of injuries	Follow-up years	Crude incidence rate (95% CI)	Crude hazard ratio (95% CI)	Hazard ratio adjusted for free school meals entitlement (95% CI)
Total					
Achieving (n = 126,226)	9,420	381,407	2.47 (2.42–2.52)		
Declining (n = 13,394)	1,269	44,450	2.85 (2.70–3.02)	1.18 (1.11–1.25)∗	1.16 (1.09–1.23)∗
Improving (n = 9,856)	727	27,103	2.68 (2.49–2.88)		
Not achieving (n = 22,925)	2,018	70,077	2.88 (2.76–3.01)	1.10 (1.01–1.19)∗	1.07 (.98–1.16)
Boys					
Achieving (n = 60,539)	5,424	180,489	3.01 (2.93–3.09)		
Declining (n = 7,330)	802	24,005	3.34 (3.12–3.58)	1.14 (1.06–1.22)∗	1.12 (1.04–1.21)∗
Improving (n = 5,910)	516	16,072	3.21 (2.95–3.50)		
Not achieving (n = 14,584)	1,392	44,245	3.15 (2.99–3.32)	1.00 (.91–1.11)	.98 (.89–1.09)
Girls					
Achieving (n = 65,687)	3,996	200,919	1.99 (1.93–2.05)		
Declining (n = 6,064)	467	20,445	2.28 (2.09–2.50)	1.17 (1.06–1.29)∗	1.13 (1.02–1.24)∗
Improving (n = 3,946)	211	11,031	1.91 (1.67–2.19)		
Not achieving (n = 8,341)	626	25,832	2.42 (2.24–2.62)	1.29 (1.10–1.50)∗	1.23 (1.05–1.44)∗

CI = confidence interval.

∗ Statistically significant at 95% CI.

Analysis A: Children who achieved KS1 but not KS2 (i.e., declining in attainment) were at higher risk of injury in adolescence (that resulted in attendance at the GP) compared with those who achieved both stages (hazard ratio: 1.14, boys; hazard ratio: 1.17, girls).

Analysis B: Girls who did not achieve both stages were at higher risk of injury compared with girls who initially did not achieve KS1 but then went on to achieve KS2 (improvers) (hazard ratio: 1.29). This was not observed in the boys (i.e., improvers and consistent nonachievers had the same risk of GP injury attendance).

Analysis C: Improving girls were less likely to present with injuries at their GP's than decliners (hazard ratio: .81; see Supplement 6).

#### Multiple general practice contacts

About 4,100 children had more than one GP visit due to an injury. The majority of children with injuries (86%) had two or three GP visits.

A group-level Cox regression was performed for all injury-related GP contacts. In this type of analysis only the group membership (i.e., consistent achiever, decliner, improver, and consistent nonachiever) was taken into account. Results for multiple GP contacts were similar to the results for single GP contacts (see Table 2).

**Table 2:** Injury rate; time to first GP contact (group level including multiple injuries per individual) for injury by key stage achievement and gender

	Number of injuries	Follow-up years	Crude incidence rate (95% CI)	Crude hazard ratio (95% CI)	Hazard ratio adjusted for free school meals entitlement (95% CI)
Total					
Achieving (n = 130,814)	13,996	392,137	3.57 (3.51–3.63)		
Declining (n = 14,155)	2,030	46,314.	4.38 (4.20–4.58)	1.24 (1.18–1.29)^∗^	1.20 (1.14–1.26)^∗^
Improving (n = 10,244)	1,115	28,017	3.98 (3.75–4.22)		
Not achieving (n = 23,987)	3,073	72,628	4.23 (4.08–4.38)	1.07 (1.00–1.14)	1.04 (.97–1.12)
Boys					
Achieving (n = 63,393)	8,271	187,123	4.42 (4.33–4.52)		
Declining (n = 7,810)	1,282	25,160.	5.10 (4.82–5.38)	1.16 (1.09–1.23)^∗^	1.14 (1.08–1.21)^∗^
Improving (n = 6,206)	812	16,761	4.84 (4.52–5.19)		
Not achieving (n = 15,379)	2,183	46,128.	4.73 (4.54–4.94)	.98 (.91–1.06)	.96 (.89–1.05)
Girls					
Achieving (n = 67,421)	5,725	205,014.	2.79 (2.72–2.87)		
Declining (n = 6,345)	748	21,154	3.54 (3.29–3.80)	1.27 (1.18–1.37)^∗^	1.20 (1.11–1.30)^∗^
Improving (n = 4,038)	303	11,256.	2.69 (2.41–3.01)		
Not achieving (n = 8,608)	890	26,499.	3.36 (3.15–3.59)	1.25 (1.10–1.42)^∗^	1.19 (1.05–1.36)

CI = confidence interval.

Analysis A: Children who achieved KS1 but not KS2 (i.e., declining in attainment) were at higher risk of injury in adolescence (that resulted in attendance at the GP) compared with those who achieved both stages (hazard ratio: 1.16, boys; hazard ratio: 1.27, girls).

Analysis B: The girls who did not achieve both stages were at higher risk of injury compared with girls who initially did not achieve KS1 but who went on to achieve KS2 (improvers) (hazard ratio: 1.25). This was not observed in the boys (i.e., improvers and consistent nonachievers had the same risk of injury).

Analysis C: Improving girls were less likely to present several times with injuries at their GP's than decliners (hazard ratio: .76; see Supplement 7).

### Hospital admission

A total of 5,370 (3.11%) children had at least one injury-related hospital admission after the age of 12 years (2.83% in the consistent achieving group and 3.85% in the consistent nonachieving group). The results of the Cox regression to the first injury admission to hospital are in Table 3. Five children had a follow-up of less than 1 day (i.e., injury on the day they left the cohort) and were excluded from analysis, leading to 172,416 children in the secondary care cohort. The mean time of follow-up from the 12th birthday to first hospital admission was 2.52 years.

**Table 3:** Injury rate; time to first hospital admission for injury by key stage achievement and gender

	Number of injuries	Follow-up years	Crude incidence rate (95% CI)	Crude hazard ratio (95% CI)	Hazard ratio adjusted for free school meals entitlement (95% CI)
Total					
Achieving (n = 126,239)	3,576	395,090	.91 (.88–.94)		
Declining (n = 13,396)	580	46,393.	1.25 (1.15–1.36)	1.40 (1.28–1.52)^∗^	1.33 (1.22–1.46)^∗^
Improving (n = 9,858)	328	28,051	1.17 (1.05–1.30)		
Not achieving (n = 22,923)	882	72,715	1.21 (1.14–1.30)	1.05 (.92–1.19)	1.03 (.90–1.17)
Boys					
Achieving (n = 60,545)	2,178	188,112	1.16 (1.11–1.21)		
Declining (n = 7,331)	385	25,193.	1.53 (1.38–1.69)	1.34 (1.20–1.50)^∗^	1.31 (1.17–1.46)^∗^
Improving (n = 5,912)	223	16,781.	1.33 (1.17–1.52)		
Not achieving (n = 14,584)	613	46,078.	1.33 (1.23–1.44)	1.01 (.87–1.18)	1.00 (.85–1.16)
Girls					
Achieving (n = 65,694)	1,398	206,978	.68 (.64–.71)		
Declining (n = 6,065)	195	21,200.	.92 (.80–1.06)	1.36 (1.17–1.58)^∗^	1.21 (1.04–1.40)^∗^
Improving (n = 3,946)	105	11,270	.93 (.77–1.13)		
Not achieving (n = 8,339)	269	26,637	1.01 (.90–1.14)	1.08 (.86–1.36)	1.06 (.84–1.32)

CI = confidence interval.

Analysis A: The children who achieved KS1 but not KS2 (i.e., declining in attainment) were at higher risk of admission for injury in adolescence compared with those who achieved both stages (consistent achievers) (hazard ratio: 1.34, boys; hazard ratio: 1.36, girls).

Analysis B: There was no significant difference between those who did not achieve at KS1 and those who did. Those who were improving in educational achievement over KS1 and KS2 were not at any different risk to those who were not achieving academically.

Analysis C: Improving boys were less likely to have an injury-related hospital admission than declining girls (hazard ratio: .85; see Supplement 8).

#### Multiple hospital admissions

Six hundred seventeen children had more than one hospital admission for injuries between the age of 12 and 18 years. The majority (80.7%) of these children had two admissions, 17 children had more than five admissions, but some of these were connected to inpatient physiotherapy visits. Children with more than five admissions were more likely to have International Classification of Diseases-10 codes relating to risk factors (e.g., history of self-harm; 13 cases) and intentional self-poisoning (12 cases).

Hazard ratios were similar to the results for single hospital admissions (see Table 4).

**Table 4:** Injury rate; time to hospital admission (group level including multiple injuries per individual) for injury by key stage achievement and gender

	Number of injuries	Follow-up years	Crude incidence rate (95% CI)	Crude hazard ratio (95% CI)	Hazard ratio adjusted for free school meals entitlement (95% CI)
Total					
Achieving (n = 126,790)	4,127	396,374	1.04 (1.01–1.07)		
Declining (n = 13,503)	687	46,656	1.47 (1.37–1.59)	1.42 (1.31–1.54)^∗^	1.33 (1.23–1.44)^∗^
Improving (n = 9,897)	367	28,140	1.30 (1.18–1.44)		
Not achieving (n = 23,049)	1,008	72,999	1.38 (1.30–1.47)	1.07 (.95–1.20)	1.05 (.93–1.18)
Boys					
Achieving (n = 60,829)	2,462	188,685.	1.30 (1.25–1.36)		
Declining (n = 7,381)	435	25,324	1.72 (1.56–1.89)	1.33 (1.21–1.48)^∗^	1.30 (1.17–1.44)^∗^
Improving (n = 5,940)	251	16,839.	1.49 (1.32–1.69)		
Not achieving (n = 14,656)	685	46,216.	1.49 (1.38–1.60)	1.01 (.87–1.16)	.99 (.86–1.14)
Girls					
Achieving (n = 65,961)	1,665	207,689	.80 (.76–.84)		
Declining (n = 6,122)	252	21,332.	1.18 (1.04–1.34)	1.47 (1.28–1.67)^∗^	1.27 (1.11–1.45)^∗^
Improving (n = 3,957)	116	11,300.	1.03 (.86–1.23)		
Not achieving (n = 8,393)	323	26,782.	1.21 (1.08–1.34)	1.17 (.94–1.44)	1.13 (.91–1.40)

CI = confidence interval.

Analysis A: The children who achieved KS1 but not KS2 (i.e., declining in attainment) were at higher risk of hospital admission for injury in adolescence compared with those who achieved both stages (hazard ratio: 1.33, boys; hazard ratio: 1.47, girls).

Analysis B: There was no significant difference between those who did not achieve at KS1. Those who were improving in educational attainment were not at any different risk to those who were not achieving academically.

Analysis C: Improving boys were less likely to have multiple injury-related hospital admissions than decliners (hazard ratio: .85; see Supplement 9).

#### Self-reported health data

There were 801 children who completed the HBSC questionnaire and 756 (94%) gave consent to routine data linkage; of these, 616 (81%) could be linked with health records and 398 (52%) could be linked to education records for both KS1 and KS2 from primary school. Of 398 (51.5% male and 48.5% female) children with educational linkage, none were in year 7 (aged 11–12 years), 142 (35.6%) were in year 8 (aged 12–13 years), 108 (27.1%) were in year 9 (aged 13–14 years), 115 (28.9%) were in year 10 (aged 14–15 years), and 33 (8.2%) were in year 11 (aged 15–16 years). The percentage of those who have ever drunk alcohol by school year in this sample was 33% (year 8), 51% (year 9), 64% (year 10), and 84% (year 11).

Analysis A: The pupils who were declining in attainment were the group most likely to be drinking alcohol (14/20, 70%) compared with those consistently achieving in primary school (171/309, 55.3%, *p* = .0001) and were more likely to have ever been intoxicated (8/20%–40%) compared with those consistently achieving (76/309, 24.6%, *p* = .088). They were also more likely to be physically inactive (self-reported physical activity less than once per week) compared with those who consistently achieved in primary school (7/20, 31.6% compared with 50/309, 16.4%, *p* = .03). Injuries in this group are thus less likely to be associated with sports injuries.

Analysis B: Among the children improving in educational attainment there were also higher levels of drinking (13/29, 44.8%) compared with those not achieving (9/40, 22.5%, *p* = .049).

## Discussion

Children at high risk of injuries in adolescence can be identified in their primary school years as those who initially were doing well in education but then decline in attainment, that is, those who pass KS1 (age: 6–7 years) but fail KS2 (age 10–11 years). Both boys and girls are consistently at higher risk of injuries in this category (i.e., declining), although the risk is slightly reduced for those children in this category eligible for free school meals. Girls that failed both KS1 and KS2 were also more likely to present with injuries at their GP.

This study also suggests that these injuries may be due to risk-taking behaviors such as alcohol consumption and that children who are not achieving well academically in primary school, especially at ages 10–11 years (i.e., completion of KS2), may benefit from being targeted with health behavior intervention (e.g., alcohol and self-harm–related interventions) as they are more likely to drink in adolescence (70%).

Externalizing behaviors (such as conduct disorders, impulsivity, and antisocial behavior), which might present in later primary school years, are known to increase injury risk in adolescents [35], [36]. These are strongly predictive of adult injuries sustained due to violent assaults and permanently disabling accidents [37] and may provide partial explanation for the higher injury rates among those in the “declining” category.

Peer effects on risk taking and risky decision-making are strong among adolescents [38] and stronger than they are among adults [39]. It is possible that those who underachieve in education may form friendship groups with similarly underachieving peers which value risk behaviors such as smoking, extreme sports, and fighting that tend to reinforce antischool values, providing a measure of social integration that further reinforces health risk behavior and disengagement from school life [40]. However, at KS2, the children in this study are probably too young to be subjected to external peer effects, and a decline in school is more likely to be linked to family dynamics.

### Limitations

Of those who did not link to health records (177 children), there were more girls (59.6%, compared with 48.5% girls in those who link) and self-reported drinking alcohol was 40% (compared with 43% in those who did have linkage). Furthermore, linkage of education records for KS1 and KS2 combined with follow-up data beyond the age of 12 years was only available for 24% of children on the education data set. This cohort study includes those children born before 2001 (to have follow-up after age 12) and therefore cannot reflect any effects of recent changes in educational policy and/or health interventions in contemporary school settings.

We are using time to injury as primary outcome variable; this allows us to adjust for each child's length of stay in the cohort. However, we might be missing very mobile children that attend hospital in neighboring home countries. We also can only make a statement about GP admissions for those children whose GPs have registered with our databank.

The ability to predict the occurrence of adverse health events associated with risk behaviors may be tempered somewhat by the existence of protective factors, such as family and material supports [40], which were not investigated.

The HBSC survey contained more children in the younger age group. It therefore provides a better measure of the health of younger rather than older adolescents (i.e., older than 15 years) [8] and gives a less than complete picture of health in older adolescence when many health risk behaviors, such as tobacco and alcohol use, other substance misuse, obesity, and physical inactivity become established [41].

Injuries may also be triggered by diseases or prevalent health conditions, which would be difficult to isolate in our data. A summary of noncommunicable diseases at KS2 (see Table 5), however, only indicates a slightly higher prevalence of asthma in children that fail KS2. Adding comorbidities to our model might explain factors between declining in attainment and injuries. However, this was not within the scope of our project and will be part of another study.

**Table 5:** Number of children with key comorbidities by key stage achievement; some children might have more than one comorbidity

	Key stage achievement group	Grand total	Number of children with asthma		Number of children with diabetes		Number of children with mental health conditions	
Analysis A	Consistent achiever	121,573	6,422	5.28%	189	.16%	315	.26%
	Decliner	12,953	830	6.41%	20	.15%	61	.47%
Analysis B	Improver	9,457	550	5.82%	8	.08%	49	.52%
	Consistent nonachiever	22,156	1,606	7.25%	30	.14%	112	.51%
	Grand total	166,139	9,408		247		537	

The strength of this study on the other hand is that it comprises of a rare combination of data sets, that is, health data, education data, and survey data.

In summary, this study finds that children at risk of alcohol, substance abuse, and self-inflicted injuries are those who are declining in primary school educational attainment. They initially enter school achieving but then decline in education between the ages 5 and 10 years. Interventions aimed at identifying and targeting children declining in educational attainment in primary school could help to improve adolescent health. This analysis suggests that the triggers for some of the outcomes are before the national assessment at age 10/11, so interventions may need to start in early primary years.
