# Supplementary material for: Educational Attainment at Age 10–11 Years Predicts Health Risk Behaviors and Injury Risk During Adolescence
Source: J Adolesc Health. 2017 Aug;61(2):212–8. doi: 10.1016/j.jadohealth.2017.02.003 (PMC5516262; doi:10.1016/j.jadohealth.2017.02.003)
Supplement: Supplement 1 [file mmc1.docx]

Supplement 1: Baseline confounders at Key Stage 1

| Boys |  |  |  | |
| --- | --- | --- | --- | --- |
|  | Total Boys | Boys with FSM entitlement | |  |
| Achieved KS1 and KS2 | 60,546 | 12.67% | |  |
| Achiever KS1 but not KS2 | 7,331 | 26.26% | |  |
| Did not achieve KS1 but KS2 | 5,912 | 26.47% | |  |
| Did not achieve either | 14,586 | 37.45% | |  |
| Girls |  |  |  | |
|  | Total Girls | Girls with FSM entitlement | |  |
| Achieved KS1 and KS2 | 65,694 | 14.57% | |  |
| Achiever KS1 but not KS2 | 6,065 | 30.95% | |  |
| Did not achieve KS1 but KS2 | 3,946 | 31.42% | |  |
| Did not achieve either | 8,341 | 41.78% | |  |
