# Supplementary material for: Educational Attainment at Age 10–11 Years Predicts Health Risk Behaviors and Injury Risk During Adolescence
Source: J Adolesc Health. 2017 Aug;61(2):212–8. doi: 10.1016/j.jadohealth.2017.02.003 (PMC5516262; doi:10.1016/j.jadohealth.2017.02.003)
Supplement: Supplement 2 [file mmc2.docx]

Supplement 2: READ injury nature codes for Family Physician visits

| **Categories** | **READ codes** |
| --- | --- |
| **Injury nature** | |
| 1 Contusion, bruise | SD..., SD0.., SD08., SD0y., SD0y0, SD0y1, SD0y2, SD0yz, SD0z., SD0z0, SD0z1, SD0z2, SD0zz, SD1.., SD18., SD1y., SD1y0, SD1y1, SD1y2, SD1y3, SD1y4, SD1y5, SD1y6, SD1y7, SD1y8, SD1y9, SD1yA, SD1yB, SD1yC, SD1yD, SD1yz, SD1z., SD1z0, SD1z1, SD1z2, SD1z3, SD1z4, SD1z5, SD1z6, SD1z7, SD1z8, SD1z9, SD1zA, SD1zB, SD1zC, SD1zD, SD1zz, SD2.., SD28., SD2y., SD2y0, SD2y1, SD2y2, SD2y3, SD2yz, SD2z., SD2z0, SD2z1, SD2z2, SD2z3, SD2zz, SD3.., SD38., SD39., SD3y., SD3y0, SD3y1, SD3y2, SD3yz, SD3z., SD3z0, SD3z1, SD3z2, SD3zz, SD4.., SD4y., SD4z., SD5.., SD5y., SD5z., SD6.., SD68., SD69., SD6A., SD6y., SD6y0, SD6y1, SD6y2, SD6y3, SD6y4, SD6yz, SD6z., SD6z0, SD6z1, SD6z2, SD6z3, SD6z4, SD6zz, SD7.., SD7y., SD7y0, SD7y1, SD7y2, SD7yz, SD7z., SD7z0, SD7z1, SD7zz, SD8.., SD80., SD80z, SD81., SD81z, SD82., SD8z., SD9.., SD9y., SD9z., SDA.., SDC.., SDC0., SDC1., SDX.., SDz.., SE..., SE0.., SE00., SE01., SE02., SE03., SE04., SE05., SE06., SE07., SE08., SE09., SE0z., SE1.., SE10., SE11., SE12., SE13., SE14., SE1z., SE2.., SE20., SE21., SE22., SE220, SE221, SE222, SE223, SE22z, SE23., SE230, SE231, SE232, SE233, SE234, SE23z, SE24., SE240, SE241, SE242, SE243, SE244, SE24z, SE25., SE2y., SE2z., SE3.., SE30., SE300, SE301, SE302, SE303, SE304, SE30y, SE30z, SE31., SE310, SE311, SE31z, SE32., SE320, SE321, SE322, SE323, SE324, SE325, SE32z, SE33., SE330, SE331, SE332, SE333, SE33z, SE3y., SE3z., SE4.., SE40., SE400, SE401, SE40z, SE41., SE410, SE411, SE41z, SE42., SE420, SE421, SE42z, SE43., SE44., SE45., SE46., SE4y., SE4z., SEz.., SK1x1, Syu00, Syu01, Syu10, Syu11, Syu12, Syu20, Syu21, Syu23, Syu24, Syu30, Syu31, Syu40, Syu50, Syu51, Syu60, Syu61, Syu70, Syu80, Syu81, Syu90, Syu91, SyuA0, SyuB9 |
| 2 Open Wound and abrasion | S8..., S80.., S800., S801., S802., S803., S804., S80y., S80z., S81.., S810., S811., S812., S813., S814., S815., S816., S817., S818., S81z., S82.., S820., S8200, S8201, S8202, S820w, S820z, S821., S8210, S8211, S8212, S821z, S822., S82v., S82v0, S82v1, S82v2, S82v3, S82vz, S82w., S82w0, S82w1, S82w2, S82w3, S82wz, S82x., S82y., S82z., S83.., S830., S8301, S831., S832., S8320, S8321, S8322, S8323, S832x, S832z, S833., S8330, S8331, S8332, S8333, S833x, S833z, S834., S8340, S8341, S8342, S8343, S8344, S8345, S8346, S834x, S834z, S835., S8350, S8351, S8352, S8353, S8354, S8355, S835x, S835z, S836., S8360, S8361, S8362, S8363, S8364, S8365, S8366, S8367, S836x, S836z, S837., S8370, S8371, S8372, S8373, S8374, S8375, S8376, S837x, S837z, S83x., S83y., S83z., S84.., S840., S8400, S8401, S8402, S840z, S841., S8410, S8411, S8412, S841z, S842., S843., S844., S845., S84x., S84x0, S84x1, S84x2, S84xz, S84y., S84y0, S84y1, S84yz, S84z., S85.., S850., S8500, S851., S852., S853., S855., S85X., S85z., S86.., S860., S8600, S861., S86z., S87.., S870., S8700, S871., S87z., S88.., S880., S8800, S8801, S881., S882., S8820, S8821, S8822, S882z, S883., S8830, S8831, S883z, S884., S8840, S8841, S8842, S884z, S885., S8850, S8851, S8852, S885z, S886., S887., S88x., S88y., S88z., S89.., S890., S891., S892., S8920, S8921, S8922, S8923, S892z, S893., S8930, S8931, S8932, S8933, S893z, S894., S8940, S8941, S8942, S8943, S8944, S8945, S894z, S895., S8950, S8951, S8952, S8953, S8954, S895z, S896., S89v., S89v0, S89v1, S89vz, S89w., S89w0, S89w1, S89wz, S89x., S89y., S89z., S8A.., S8W.., S8X.., S8z.., S9..., S90.., S900., S9000, S9001, S9002, S9003, S9004, S900x, S900z, S901., S9010, S9011, S9012, S9013, S901x, S901z, S902., S9020, S9021, S9022, S9023, S9024, S9025, S9026, S9027, S9028, S9029, S902x, S902z, S903., S9030, S9031, S9032, S9033, S904., S905., S906., S90z., S91.., S910., S9100, S9101, S9102, S9103, S9104, S910z, S911., S9110, S9111, S9112, S911z, S912., S9120, S9121, S9122, S9123, S9124, S9125, S9126, S912z, S913., S9130, S9131, S9132, S9133, S914., S915., S91z., S92.., S920., S9200, S9201, S9202, S921., S922., S9220, S9221, S9222, S9223, S923., S9230, S9231, S924., S925., S92z., S93.., S930., S9300, S9301, S9302, S931., S932., S9320, S9321, S9322, S9323, S9324, S9325, S9326, S9327, S9328, S9329, S932A, S932B, S932C, S932D, S933., S9330, S9331, S9332, S9333, S9334, S9335, S934., S935., S936., S937., S938., S93z., S94.., S940., S941., S942., S94z., S95.., S950., S9500, S9501, S9502, S9503, S9504, S9505, S9506, S9507, S9508, S951., S95z., S96.., S960., S9600, S9601, S9602, S9603, S9604, S9605, S9606, S9607, S9608, S9609, S960A, S961., S96X., S96z., S97.., S970., S9700, S9701, S9702, S9703, S970X, S971., S972., S9720, S9721, S9722, S973., S974., S975., S976., S977., S97X., S97z., S9z.., SA..., SA0.., SA00., SA000, SA001, SA00z, SA01., SA010, SA011, SA01z, SA02., SA020, SA021, SA022, SA023, SA024, SA025, SA02z, SA03., SA030, SA031, SA04., SA05., SA0z., SA1.., SA10., SA100, SA101, SA102, SA10z, SA11., SA110, SA111, SA112, SA11z, SA12., SA120, SA121, SA122, SA123, SA124, SA125, SA126, SA12z, SA13., SA130, SA131, SA132, SA14., SA15., SA16., SA1z., SA2.., SA20., SA201, SA202, SA203, SA21., SA22., SA220, SA221, SA222, SA223, SA23., SA230, SA231, SA232, SA24., SA25., SA2z., SA3.., SA30., SA31., SA32., SA33., SA330, SA331, SA34., SA3z., SA4.., SA40., SA41., SA42., SA4z., SA5.., SA50., SA500, SA501, SA502, SA51., SA5z., SA6.., SA60., SA600, SA61., SA62., SA63., SA6z., SA7.., SA70., SA701, SA702, SA71., SA72., SA720, SA721, SA722, SA73., SA74., SA75., SA76., SA77., SA78., SA7X., SA7z., SA8.., SA9.., SAA.., SAz.., SD00., SD000, SD001, SD002, SD00z, SD01., SD010, SD011, SD012, SD01z, SD10., SD100, SD101, SD102, SD103, SD104, SD105, SD106, SD107, SD108, SD109, SD10A, SD10B, SD10C, SD10D, SD10z, SD11., SD110, SD111, SD112, SD113, SD114, SD115, SD116, SD117, SD118, SD119, SD11A, SD11B, SD11C, SD11D, SD11z, SD20., SD200, SD201, SD202, SD203, SD204, SD205, SD20z, SD21., SD210, SD211, SD212, SD213, SD21z, SD30., SD300, SD301, SD302, SD303, SD30z, SD31., SD310, SD311, SD312, SD31z, SD40., SD400, SD401, SD41., SD50., SD500, SD501, SD502, SD51., SD60., SD600, SD601, SD602, SD603, SD604, SD60z, SD61., SD610, SD611, SD612, SD613, SD614, SD61z, SD70., SD700, SD701, SD70z, SD71., SD710, SD711, SD71z, SD800, SD810, SD811, SD90., SD91., SK1x2, SK1x5, SK1x7, SK1x8, SK1x9, SK1xA, SQ..., Syu02, Syu08, Syu09, Syu0A, Syu0M, Syu13, Syu14, Syu22, Syu25, Syu26, Syu32, Syu33, Syu41, Syu52, Syu62, Syu71, Syu82, Syu92, Syu93, SyuA1, SyuBA, TG304, TGyz7, TP51. |
| 3 Fractures | S0..., S00.., S000., S0000, S0001, S0002, S0003, S0004, S0005, S0006, S000z, S001., S0010, S0011, S0012, S0013, S0014, S0015, S0016, S001z, S002., S0020, S0021, S0022, S0023, S0024, S0025, S0026, S002z, S003., S0030, S0031, S0032, S0033, S0034, S0035, S0036, S003z, S00z., S01.., S010., S0100, S0101, S0102, S0103, S0104, S0105, S0106, S010z, S011., S0110, S0111, S0112, S0113, S0114, S0115, S0116, S011z, S012., S0120, S0121, S0122, S0123, S0124, S0125, S0126, S012z, S013., S0130, S0131, S0132, S0133, S0134, S0135, S0136, S013z, S01z., S02.., S020., S021., S022., S0220, S0221, S0222, S0223, S0224, S0225, S0226, S0227, S0228, S022x, S022z, S023., S0230, S0231, S0232, S0233, S0234, S0235, S0236, S0237, S0238, S023x, S023z, S024., S0240, S0241, S024z, S025., S0250, S0251, S025z, S026., S027., S028., S0280, S0281, S0282, S0283, S02A., S02B., S02C., S02x., S02x0, S02x1, S02x2, S02xz, S02y., S02y0, S02y1, S02y2, S02yz, S02z., S03.., S030., S0300, S0301, S0302, S0303, S0304, S0305, S0306, S030z, S031., S0310, S0311, S0312, S0313, S0314, S0315, S0316, S031z, S032., S0320, S0321, S0322, S0323, S0324, S0325, S0326, S032z, S033., S0330, S0331, S0332, S0333, S0334, S0335, S0336, S033z, S03z., S04.., S040., S0400, S0401, S0402, S0403, S0404, S0405, S0406, S040z, S041., S0410, S0411, S0412, S0413, S0414, S0415, S0416, S041z, S042., S0420, S0421, S0422, S0423, S0424, S0425, S0426, S042z, S043., S0430, S0431, S0432, S0433, S0434, S0435, S0436, S043z, S044., S04z., S0z.., S1..., S10.., S100., S1000, S1001, S1002, S1003, S1004, S1005, S1006, S1007, S1008, S1009, S100A, S100B, S100C, S100D, S100E, S100F, S100G, S100H, S100J, S100K, S100L, S100M, S100N, S100x, S100z, S101., S1010, S1011, S1012, S1013, S1014, S1015, S1016, S1017, S1018, S1019, S101A, S101B, S101C, S101D, S101E, S101F, S101G, S101H, S101J, S101K, S101L, S101M, S101N, S101x, S101z, S102., S1020, S1021, S1022, S1023, S1024, S1025, S1026, S102y, S102z, S103., S1030, S1031, S1032, S1033, S1034, S1035, S1036, S104., S1040, S1041, S1042, S1043, S1044, S1045, S1046, S105., S1050, S1051, S1052, S1053, S1054, S1055, S1056, S106., S1060, S1061, S107., S1070, S1071, S108., S109., S10A., S10A0, S10A1, S10A2, S10B., S10B0, S10B1, S10B2, S10B3, S10B4, S10B5, S10B6, S10x., S10y., S10z., S11.., S110., S1100, S1101, S1102, S1103, S1104, S1105, S1106, S1107, S1108, S1109, S110A, S110B, S110z, S111., S1110, S1111, S1112, S1113, S1114, S1115, S1116, S1117, S1118, S1119, S111A, S111B, S111z, S112., S1120, S1121, S1122, S1123, S1124, S1125, S1126, S1127, S1128, S1129, S112A, S112B, S112z, S113., S1130, S1131, S1132, S1133, S1134, S1135, S1136, S1137, S1138, S1139, S113A, S113B, S113z, S114., S1140, S1141, S1142, S1143, S1144, S1145, S115., S1150, S1151, S1152, S1153, S1154, S1155, S115z, S116., S1160, S1161, S1162, S1163, S116z, S117., S1170, S1171, S1172, S1173, S117z, S118., S1180, S1181, S1182, S1183, S118z, S119., S1190, S1191, S1192, S1193, S119z, S11x., S11y., S11z., S12.., S120., S1200, S1201, S1202, S1203, S1204, S1205, S1206, S1207, S1208, S1209, S120A, S120z, S121., S1210, S1211, S1212, S1213, S1214, S1215, S1216, S1217, S1218, S1219, S121z, S122., S123., S124., S1240, S1241, S125., S1250, S1251, S1252, S1253, S125z, S126., S1260, S1261, S1262, S1263, S126z, S127., S1270, S1271, S128., S12X., S12X0, S12X1, S12y., S12y0, S12y1, S12z., S13.., S130., S1300, S1301, S1302, S1303, S1304, S1305, S1306, S130y, S130z, S131., S1310, S1311, S1312, S1313, S1314, S1315, S1316, S131y, S131z, S132., S1320, S1321, S1322, S132y, S132z, S133., S1330, S1331, S1332, S133y, S133z, S134., S1340, S1341, S1342, S1343, S1344, S1345, S1346, S1347, S1348, S134z, S135., S1350, S1351, S1352, S1353, S1354, S1355, S1356, S1357, S1358, S135y, S135z, S136., S1360, S1361, S137., S1370, S1371, S138., S13y., S13z., S14.., S140., S141., S14z., S15.., S150., S1500, S1501, S1z.., S2..., S20.., S200., S2000, S2001, S2002, S2003, S200z, S201., S2010, S2011, S2012, S2013, S201z, S20z., S21.., S210., S2100, S2101, S2102, S2103, S2104, S2105, S2106, S210z, S211., S2110, S2111, S2112, S2113, S2114, S2115, S2116, S211z, S21z., S22.., S220., S2200, S2201, S2202, S2203, S2204, S2205, S2206, S2207, S220z, S221., S2210, S2211, S2212, S2213, S2214, S2215, S2216, S2217, S221z, S222., S2220, S2221, S222z, S223., S2230, S2231, S223z, S224., S2240, S2241, S2242, S2243, S2244, S2245, S2246, S2247, S2248, S2249, S224x, S224z, S225., S2250, S2251, S2252, S2253, S2254, S2255, S2256, S2257, S2258, S2259, S225x, S225z, S226., S227., S228., S22z., S23.., S230., S2300, S2301, S2302, S2303, S2304, S2305, S2306, S2307, S2308, S2309, S230A, S230B, S230z, S231., S2310, S2311, S2312, S2313, S2314, S2315, S2316, S2317, S2318, S2319, S231A, S231B, S231z, S232., S2320, S2321, S2322, S2323, S232z, S233., S2330, S2331, S2332, S2333, S233z, S234., S2340, S2341, S2342, S2343, S2344, S2345, S2346, S2347, S2348, S2349, S234A, S234B, S234C, S234D, S234E, S234F, S234G, S234z, S235., S2350, S2351, S2352, S2353, S2354, S2355, S2356, S2357, S2358, S2359, S235A, S235B, S235C, S235D, S235E, S235F, S235z, S236., S237., S238., S239., S23A., S23B., S23C., S23x., S23x0, S23x1, S23x2, S23x3, S23xz, S23y., S23y0, S23y1, S23y2, S23y3, S23yz, S23z., S24.., S240., S2400, S2401, S2402, S2403, S2404, S2405, S2406, S2407, S2408, S2409, S240A, S240B, S240C, S240D, S240E, S240F, S240y, S240z, S241., S2410, S2411, S2412, S2413, S2414, S2415, S2416, S2417, S2418, S2419, S241A, S241B, S241C, S241D, S241E, S241F, S241y, S241z, S242., S2420, S2421, S2422, S2423, S24z., S25.., S250., S2500, S2501, S2502, S2503, S2504, S2505, S2506, S2507, S2508, S2509, S250A, S250B, S250C, S250x, S250z, S251., S2510, S2511, S2512, S2513, S2514, S2515, S2516, S2517, S2518, S2519, S251A, S251B, S251C, S251x, S251z, S252., S253., S26.., S260., S2600, S2601, S2602, S2603, S2604, S2605, S2606, S2607, S2608, S2609, S260A, S260B, S260C, S260D, S260E, S260F, S260G, S260H, S260J, S260K, S260L, S260M, S260N, S260P, S260Q, S260R, S260S, S260T, S260U, S260V, S260W, S260x, S260z, S261., S2610, S2611, S2612, S2613, S2614, S2615, S2616, S2617, S2618, S2619, S261A, S261B, S261C, S261D, S261E, S261F, S261G, S261H, S261J, S261K, S261L, S261M, S261N, S261P, S261Q, S261R, S261S, S261T, S261U, S261V, S261W, S261x, S261z, S262., S263., S264., S26z., S27.., S270., S271., S27z., S28.., S280., S281., S28z., S29.., S290., S291., S292., S2920, S2921, S293., S294., S2940, S2941, S29z., S2A.., S2B.., S2z.., S3..., S30.., S300., S3000, S3001, S3002, S3003, S3004, S3005, S3006, S3007, S3008, S3009, S300A, S300y, S300z, S301., S3010, S3011, S3012, S3013, S3014, S3015, S3016, S3017, S3018, S3019, S301A, S301y, S301z, S302., S3020, S3021, S3022, S3023, S3024, S302z, S303., S3030, S3031, S3032, S3033, S3034, S303z, S304., S305., S30w., S30x., S30y., S30z., S31.., S310., S3100, S3101, S310z, S311., S3110, S3111, S311z, S312., S3120, S3121, S3122, S3123, S3124, S3125, S3126, S312x, S312z, S313., S3130, S3131, S3132, S3133, S3134, S3135, S3136, S313x, S313z, S314., S315., S31z., S32.., S320., S3200, S3201, S3202, S3203, S3204, S321., S3210, S3211, S3212, S3213, S3214, S32z., S33.., S330., S3300, S3301, S3302, S3303, S3304, S3305, S3306, S3307, S3308, S3309, S330z, S331., S3310, S3311, S3312, S3313, S3314, S3315, S3316, S3317, S3318, S3319, S331A, S331z, S332., S3320, S3321, S3322, S332z, S333., S3330, S3331, S3332, S333z, S334., S3340, S3341, S335., S3350, S3351, S336., S3360, S337., S338., S339., S3390, S3391, S33A., S33B., S33C., S33x., S33x0, S33x1, S33x2, S33xz, S33y., S33y0, S33y1, S33y2, S33yz, S33z., S34.., S340., S341., S342., S3420, S3421, S343., S3430, S3431, S344., S3440, S3441, S345., S3450, S3451, S346., S3460, S3461, S347., S3470, S3471, S348., S349., S34x., S34y., S34z., S35.., S350., S3500, S3501, S351., S3510, S3511, S352., S3520, S3521, S3522, S3523, S3524, S3525, S3526, S3527, S3528, S3529, S352A, S352B, S352C, S352D, S352E, S352F, S352G, S352H, S352J, S352z, S353., S3530, S3531, S3532, S3533, S3534, S3535, S3536, S3537, S3538, S3539, S353A, S353B, S353C, S353D, S353E, S353F, S353G, S353H, S353J, S353z, S354., S355., S356., S35z., S36.., S360., S3600, S3601, S3602, S3603, S361., S3610, S3611, S3612, S3613, S362., S3620, S3621, S363., S36z., S37.., S370., S371., S3x.., S3X.., S3x0., S3x1., S3x2., S3x3., S3x4., S3xz., S3y.., S3y0., S3y1., S3yz., S3z.., S3z0., S3z00, S3z1., S3z2., S3zz., SR1.., SR10., SR100, SR101, SR11., SR12., SR120, SR121, SR13., SR14., SR140, SR141, SR15., SR150, SR151, SR16., SR160, SR161, SR1z., SR1z0, SR1z1, Syu03, Syu04, Syu15, Syu16, Syu27, Syu28, Syu34, Syu42, Syu43, Syu44, Syu53, Syu54, Syu63, Syu64, Syu65, Syu72, Syu83, Syu8D, Syu94, SyuA2, SyuBB |
| 4 Dislocation and subluxation | S4..., S40.., S400., S401., S402., S403., S40z., S41.., S410., S4100, S4101, S4102, S4103, S4104, S4105, S410y, S410z, S411., S4110, S4111, S4112, S4113, S4114, S4115, S411y, S411z, S412., S4120, S4121, S412z, S413., S4130, S4131, S413z, S41z., S42.., S420., S4200, S4201, S4202, S4203, S4204, S4205, S4206, S420y, S420z, S421., S4210, S4211, S4212, S4213, S4214, S4215, S4216, S421y, S421z, S422., S4220, S4221, S423., S4230, S4231, S424., S42z., S43.., S430., S4300, S4301, S4302, S4303, S4304, S4305, S4306, S4307, S4308, S430y, S430z, S431., S4310, S4311, S4312, S4313, S4314, S4315, S4316, S4317, S4318, S431y, S431z, S432., S4320, S4321, S4322, S4323, S4324, S4325, S4326, S432y, S433., S4330, S4331, S4332, S4333, S4334, S4335, S4336, S433y, S43z., S44.., S440., S4400, S4401, S4402, S4403, S4404, S4405, S4406, S440z, S441., S4410, S4411, S4412, S4413, S4414, S4415, S4416, S441z, S442., S4420, S4421, S4422, S4423, S4424, S4425, S443., S4430, S4431, S4432, S4433, S4434, S4435, S44z., S45.., S450., S4500, S4501, S4502, S4503, S450z, S451., S4510, S4511, S4512, S4513, S451z, S452., S4520, S4521, S4522, S453., S4530, S4531, S4532, S45z., S46.., S460., S4600, S4601, S4602, S4603, S4604, S4605, S461., S4610, S4611, S4612, S4613, S4614, S4615, S462., S463., S4630, S4631, S464., S4640, S4641, S465., S4650, S4651, S4652, S4653, S4654, S4655, S4656, S465z, S466., S4660, S4661, S4662, S4663, S4664, S4665, S4666, S466z, S467., S4670, S4671, S468., S4680, S4681, S469., S4690, S4691, S4692, S4693, S4694, S4695, S4696, S46A., S46A0, S46A1, S46A2, S46A3, S46A4, S46A5, S46A6, S46B., S46C., S46D., S46z., S47.., S470., S471., S472., S473., S47z., S48.., S480., S4800, S4801, S4802, S4803, S4804, S4805, S4806, S4807, S4808, S4809, S480A, S480z, S481., S4810, S4811, S4812, S4813, S4814, S4815, S4816, S4817, S4818, S4819, S481A, S481z, S482., S4820, S4821, S4822, S4823, S4824, S4825, S4826, S4827, S4828, S4829, S483., S4830, S4831, S4832, S4833, S4834, S4835, S4836, S4837, S4838, S4839, S48z., S49.., S490., S4900, S4901, S4902, S4903, S4904, S4905, S4906, S4907, S4908, S4909, S490A, S490B, S490C, S490D, S490x, S490z, S491., S4910, S4911, S4912, S4913, S4914, S4915, S4916, S4917, S4918, S4919, S491A, S491B, S491C, S491D, S491x, S491z, S492., S4920, S4921, S4922, S4923, S4924, S4925, S4926, S4927, S4928, S4929, S492A, S492B, S492C, S492z, S493., S4930, S4931, S4932, S4933, S4934, S4935, S4936, S4937, S4938, S4939, S493A, S493B, S493C, S493z, S494., S4940, S4941, S4942, S494z, S495., S4950, S4951, S4952, S495z, S496., S4960, S4961, S4962, S4963, S4964, S4965, S4966, S4967, S496z, S497., S4970, S4971, S4972, S4973, S4974, S4975, S4976, S4977, S497z, S498., S4980, S4981, S4982, S4983, S4984, S4985, S4986, S4987, S4988, S4989, S498A, S498B, S498C, S498D, S498x, S498z, S499., S4990, S4991, S4992, S4993, S4994, S4995, S4996, S4997, S4998, S4999, S499A, S499B, S499C, S499D, S499x, S499z, S49A., S49A0, S49A1, S49A2, S49A3, S49A4, S49A5, S49A6, S49A7, S49A8, S49A9, S49AA, S49AB, S49AC, S49Az, S49B., S49B0, S49B1, S49B2, S49B3, S49B4, S49B5, S49B6, S49B7, S49B8, S49B9, S49BA, S49BB, S49BC, S49Bz, S49C., S49C0, S49C1, S49C2, S49Cz, S49D., S49D0, S49D1, S49D2, S49Dz, S49E., S49E0, S49E1, S49E2, S49E3, S49E4, S49E5, S49E6, S49E7, S49Ez, S49F., S49F0, S49F1, S49F2, S49F3, S49F4, S49F5, S49F6, S49F7, S49Fz, S49G., S49x., S49X., S49X0, S49y., S49z., S4A.., S4A0., S4A00, S4A01, S4A1., S4A10, S4A11, S4A2., S4A20, S4A21, S4A3., S4A30, S4A31, S4B.., S4B0., S4B00, S4B01, S4B1., S4B10, S4B11, S4B2., S4B20, S4B21, S4B3., S4B30, S4B31, S4C.., S4C0., S4C00, S4C01, S4C02, S4C03, S4C04, S4C05, S4C06, S4C0y, S4C1., S4C10, S4C11, S4C12, S4C13, S4C14, S4C15, S4C16, S4C1y, S4C2., S4C20, S4C21, S4C22, S4C23, S4C24, S4C25, S4C26, S4C2y, S4C3., S4C30, S4C31, S4C32, S4C33, S4C34, S4C35, S4C36, S4C3y, S4D.., S4D0., S4D00, S4D01, S4D02, S4D03, S4D04, S4D05, S4D06, S4D1., S4D10, S4D11, S4D12, S4D13, S4D14, S4D15, S4D16, S4D2., S4D20, S4D21, S4D22, S4D23, S4D24, S4D25, S4D26, S4D3., S4D30, S4D31, S4D32, S4D33, S4D34, S4D35, S4D36, S4E.., S4E0., S4E1., S4E2., S4E3., S4F.., S4F0., S4F1., S4F2., S4F3., S4F4., S4F5., S4F6., S4F7., S4G.., S4G0., S4G1., S4G2., S4G3., S4H.., S4H0., S4H00, S4H01, S4H02, S4H03, S4H04, S4H05, S4H06, S4H1., S4H10, S4H11, S4H12, S4H13, S4H14, S4H15, S4H16, S4H2., S4H20, S4H21, S4H22, S4H23, S4H24, S4H25, S4H26, S4H3., S4H30, S4H31, S4H32, S4H33, S4H34, S4H35, S4H36, S4J.., S4J0., S4J00, S4J01, S4J02, S4J03, S4J1., S4J10, S4J11, S4J12, S4J13, S4J2., S4J20, S4J21, S4J22, S4J23, S4J3., S4J30, S4J31, S4J32, S4J33, S4z.., Syu05, Syu17, Syu29, Syu35, Syu45, Syu95 |
| 5 Sprain and strain | S5..., S50.., S500., S501., S502., S503., S504., S505., S506., S507., S5070, S5071, S508., S509., S50A., S50w., S50x., S50X., S50y., S50z., S51.., S510., S511., S512., S513., S51w., S51x., S51y., S51z., S52.., S520., S5200, S5201, S5202, S5203, S5204, S5205, S5206, S5207, S5208, S5209, S520A, S520B, S520C, S520D, S520E, S520F, S520G, S520H, S520J, S520K, S520L, S520M, S520z, S521., S5210, S5211, S5212, S5213, S5214, S521z, S522., S5220, S5221, S5222, S5223, S5224, S5225, S5226, S5227, S523., S5230, S5231, S5232, S5233, S5234, S5235, S5236, S5237, S5238, S5239, S523A, S523B, S523C, S523D, S523E, S523F, S524., S5240, S5241, S525., S5250, S5251, S526., S5260, S5261, S5262, S52z., S53.., S530., S531., S532., S533., S534., S535., S53w., S53x., S53y., S53z., S54.., S540., S5400, S5401, S541., S5410, S5411, S542., S5421, S5422, S543., S544., S545., S54w., S54x., S54x1, S54y., S54z., S55.., S550., S5500, S5501, S5502, S5503, S5504, S5505, S5506, S550z, S551., S5510, S5511, S5512, S5513, S5514, S5515, S5516, S551z, S55z., S56.., S560., S561., S5610, S5611, S562., S563., S564., S56y., S56z., S57.., S570., S5700, S5701, S5702, S5703, S5704, S570z, S571., S572., S573., S5730, S5731, S573z, S574., S57X., S57z., S57z0, S58.., S580., S581., S58z., S59.., S590., S591., S59z., S5A.., S5A0., S5A00, S5A01, S5A02, S5A03, S5A04, S5A05, S5A06, S5A07, S5A08, S5A09, S5A0A, S5A0B, S5A0C, S5A0D, S5A0E, S5A0F, S5A0G, S5A0H, S5A0J, S5A0z, S5A1., S5A10, S5A11, S5A12, S5A13, S5A1z, S5A2., S5A20, S5A21, S5A22, S5A23, S5A24, S5A25, S5A2z, S5Az., S5B.., S5B0., S5By., S5Bz., S5C.., S5C0., S5C1., S5C2., S5C3., S5Cy., S5Cz., S5D.., S5D0., S5D00, S5D01, S5D0z, S5D1., S5D10, S5D11, S5D12, S5D13, S5D1z, S5Dz., S5E.., S5E0., S5E1., S5E2., S5E20, S5E21, S5E2z, S5E3., S5E30, S5E31, S5E32, S5E3z, S5Ez., S5F.., S5F0., S5F1., S5Fz., S5G.., S5G0., S5G1., S5G2., S5G3., S5Gy., S5Gz., S5H.., S5H0., S5H00, S5H01, S5H0z, S5H1., S5H10, S5H11, S5H12, S5H13, S5H1z, S5H2., S5H20, S5H21, S5H22, S5H23, S5H24, S5H25, S5H2z, S5Hz., S5J.., S5J0., S5Jy., S5Jz., S5K.., S5K0., S5K1., S5K2., S5K3., S5K4., S5Ky., S5Kz., S5L.., S5L0., S5L00, S5L01, S5L02, S5L03, S5L0z, S5L1., S5L10, S5L11, S5L12, S5L13, S5L1z, S5Lz., S5M.., S5M0., S5M1., S5M2., S5M3., S5M30, S5M31, S5M3z, S5M4., S5M5., S5My., S5Mz., S5N.., S5N0., S5N1., S5N2., S5N3., S5Nz., S5P.., S5P0., S5P00, S5P0z, S5P1., S5P10, S5P11, S5P12, S5P1z, S5P2., S5P20, S5P21, S5P2z, S5P3., S5P30, S5P31, S5P32, S5P3z, S5Pz., S5Q.., S5Q0., S5Q1., S5Q2., S5Q3., S5Q4., S5Q5., S5Q6., S5Qz., S5R.., S5R0., S5R1., S5Rz., S5S.., S5S0., S5S1., S5S2., S5Sz., S5T.., S5T0., S5T1., S5T2., S5T3., S5Tz., S5U.., S5U0., S5U1., S5U2., S5Uz., S5V.., S5V0., S5V1., S5V2., S5V3., S5Vz., S5W.., S5y.., S5y0., S5y1., S5y10, S5y11, S5y1z, S5y2., S5y20, S5y21, S5y22, S5y23, S5y2z, S5y3., S5y30, S5y31, S5y32, S5y3z, S5y4., S5y40, S5y41, S5y42, S5y43, S5y4z, S5y5., S5y50, S5y51, S5y52, S5y53, S5y54, S5y55, S5y56, S5y57, S5y5z, S5yX., S5yy., S5yz., S5yz1, S5z.., Syu06, Syu18, Syu2A, Syu36, Syu46, Syu66, Syu84, Syu96 |
| 6 Concussion or brain injury | S6..., S60.., S600., S601., S602., S603., S604., S605., S60z., S61.., S610., S6100, S6101, S6102, S6103, S6104, S6105, S6106, S610z, S611., S6110, S6111, S6112, S6113, S6114, S6115, S6116, S611z, S612., S6120, S6121, S6122, S6123, S6124, S6125, S6126, S612z, S613., S6130, S6131, S6132, S6133, S6134, S6135, S6136, S613z, S614., S6140, S6141, S6142, S6143, S6144, S6145, S6146, S614z, S615., S6150, S6151, S6152, S6153, S6154, S6155, S6156, S615z, S616., S6160, S6161, S6162, S6163, S6164, S6165, S6166, S616z, S617., S6170, S6171, S6172, S6173, S6174, S6175, S6176, S617z, S61x., S61x0, S61x1, S61x2, S61x3, S61x4, S61x5, S61x6, S61xz, S61y., S61y0, S61y1, S61y2, S61y3, S61y4, S61y5, S61y6, S61yz, S61z., S62.., S620., S6200, S6201, S6202, S6203, S6204, S6205, S6206, S620z, S621., S6210, S6211, S6212, S6213, S6214, S6215, S6216, S621z, S622., S6220, S6221, S6222, S6223, S6224, S6225, S6226, S622z, S623., S6230, S6231, S6232, S6233, S6234, S6235, S6236, S623z, S624., S6240, S6241, S6242, S6243, S6244, S6245, S6246, S624z, S625., S6250, S6251, S6252, S6253, S6254, S6255, S6256, S625z, S626., S627., S628., S629., S6290, S6291, S62A., S62A0, S62A1, S62z., S63.., S630., S6300, S6301, S6302, S6303, S6304, S6305, S6306, S630z, S631., S6310, S6311, S6312, S6313, S6314, S6315, S6316, S631z, S63z., S64.., S640., S6400, S6401, S6402, S6403, S6404, S6405, S6406, S640z, S641., S6410, S6411, S6412, S6413, S6414, S6415, S6416, S641z, S642., S6420, S6421, S643., S644., S645., S6450, S6451, S646., S6460, S64z., S6z.., SJ0.., SJ00., SJ01., SJ02., SJ03., SJ0z., SJ1.., SJ10., SJ11., SJ12., SJ13., SRy0. |
| 7 Foreign body | SG..., SG0.., SG00., SG01., SG02., SG020, SG03., SG0y., SG0y0, SG0z., SG1.., SG10., SG11., SG1z., SG2.., SG20., SG21., SG22., SG2z., SG3.., SG30., SG300, SG30z, SG31., SG3z., SG4.., SG40., SG41., SG42., SG43., SG4z., SG5.., SG50., SG51., SG52., SG5z., SG6.., SG60., SG61., SG62., SG63., SG64., SG65., SG66., SG67., SG68., SG6z., SG7.., SG70., SG71., SG7z., SG8.., SG9.., SG90., SG900, SG901, SG90z, SG91., SG92., SG920, SG921, SG92z, SG93., SG9z., SGz.., SyuC., SyuC0, SyuC1, SyuC2, SyuC3, TF2.., TF20., TF200, TF201, TF20z, TF21., TF22., TF220, TF221, TF222, TF22z, TF2z., TF4.., TF5.., U11Q., U11Q0, U11Q1, U11Q2, U11Q3, U11Q4, U11Q5, U11Q6, U11Q7, U11Qy, U11Qz, U11R., U11R0, U11R1, U11R2, U11R3, U11R4, U11R5, U11R6, U11R7, U11Ry, U11Rz |
| 8 Burns and scalds | SH..., SH0.., SH00., SH01., SH02., SH03., SH04., SH05., SH050, SH0x., SH0y., SH0z., SH1.., SH10., SH100, SH101, SH102, SH103, SH104, SH105, SH106, SH107, SH108, SH109, SH10x, SH10z, SH11., SH110, SH111, SH112, SH113, SH114, SH115, SH116, SH117, SH118, SH119, SH11x, SH11z, SH12., SH120, SH121, SH122, SH123, SH124, SH125, SH126, SH127, SH128, SH129, SH12A, SH12B, SH12C, SH12D, SH12E, SH12F, SH12G, SH12H, SH12J, SH12K, SH12x, SH12z, SH13., SH130, SH131, SH132, SH133, SH134, SH135, SH136, SH137, SH138, SH139, SH13A, SH13x, SH13z, SH14., SH140, SH141, SH142, SH143, SH144, SH145, SH146, SH147, SH148, SH149, SH14x, SH14z, SH15., SH150, SH151, SH152, SH153, SH154, SH155, SH156, SH157, SH158, SH159, SH15x, SH15z, SH16., SH160, SH161, SH1z., SH2.., SH20., SH200, SH201, SH202, SH203, SH204, SH205, SH206, SH20x, SH20z, SH21., SH210, SH211, SH212, SH213, SH214, SH215, SH216, SH217, SH21x, SH21z, SH22., SH220, SH221, SH222, SH223, SH224, SH225, SH226, SH227, SH228, SH229, SH22A, SH22B, SH22C, SH22D, SH22E, SH22x, SH22z, SH23., SH230, SH231, SH232, SH233, SH234, SH235, SH236, SH237, SH23x, SH23z, SH24., SH240, SH241, SH242, SH243, SH244, SH245, SH246, SH24x, SH24z, SH25., SH250, SH251, SH252, SH253, SH254, SH255, SH256, SH25x, SH25z, SH26., SH2z., SH3.., SH30., SH300, SH301, SH302, SH303, SH304, SH305, SH306, SH30x, SH30z, SH31., SH310, SH311, SH312, SH313, SH314, SH315, SH316, SH31x, SH31z, SH32., SH320, SH321, SH322, SH323, SH324, SH325, SH326, SH327, SH328, SH329, SH32A, SH32B, SH32C, SH32D, SH32x, SH32z, SH33., SH330, SH331, SH332, SH333, SH334, SH335, SH336, SH33x, SH33z, SH34., SH340, SH341, SH342, SH343, SH344, SH345, SH346, SH34x, SH34z, SH35., SH350, SH351, SH352, SH353, SH354, SH355, SH356, SH35x, SH35z, SH36., SH360, SH361, SH362, SH3z., SH4.., SH40., SH400, SH401, SH402, SH403, SH404, SH405, SH406, SH407, SH40x, SH40z, SH41., SH410, SH411, SH412, SH413, SH414, SH415, SH416, SH417, SH41x, SH41z, SH42., SH420, SH421, SH422, SH423, SH424, SH425, SH426, SH427, SH428, SH429, SH42A, SH42B, SH42C, SH42D, SH42E, SH42F, SH42x, SH42z, SH43., SH430, SH431, SH432, SH433, SH434, SH435, SH436, SH437, SH43x, SH43z, SH44., SH440, SH441, SH442, SH443, SH444, SH445, SH446, SH447, SH44x, SH44z, SH45., SH450, SH451, SH452, SH453, SH454, SH455, SH456, SH457, SH45x, SH45z, SH46., SH460, SH461, SH462, SH4z., SH5.., SH50., SH500, SH501, SH502, SH503, SH504, SH505, SH506, SH50x, SH50z, SH51., SH510, SH511, SH512, SH513, SH514, SH515, SH516, SH51x, SH51z, SH52., SH520, SH521, SH522, SH523, SH524, SH525, SH526, SH527, SH528, SH529, SH52A, SH52B, SH52C, SH52D, SH52x, SH52z, SH53., SH530, SH531, SH532, SH533, SH534, SH535, SH536, SH53x, SH53z, SH54., SH540, SH541, SH542, SH543, SH544, SH545, SH546, SH54x, SH54z, SH55., SH550, SH551, SH552, SH553, SH554, SH555, SH556, SH55x, SH55z, SH56., SH560, SH561, SH562, SH57., SH570, SH571, SH572, SH5z., SH6.., SH60., SH61., SH62., SH620, SH621, SH63., SH64., SH65., SH66., SH660, SH661, SH663, SH6z., SH6z0, SH6z1, SH6z2, SH7.., SH70., SH700, SH701, SH702, SH703, SH704, SH70z, SH71., SH710, SH711, SH712, SH713, SH714, SH71X, SH71z, SH72., SH720, SH73., SH730, SH731, SH732, SH733, SH73z, SH74., SH740, SH741, SH74z, SH7y., SH7y0, SH7z., SH8.., SH80., SH800, SH801, SH80z, SH81., SH810, SH811, SH812, SH813, SH814, SH81z, SH82., SH820, SH821, SH822, SH823, SH82z, SH83., SH830, SH831, SH832, SH833, SH834, SH83z, SH84., SH840, SH841, SH842, SH843, SH844, SH845, SH84z, SH85., SH850, SH851, SH852, SH853, SH854, SH855, SH856, SH85z, SH86., SH860, SH861, SH862, SH863, SH864, SH865, SH866, SH867, SH86z, SH87., SH870, SH871, SH872, SH873, SH874, SH875, SH876, SH877, SH878, SH87z, SH88., SH880, SH881, SH882, SH883, SH884, SH885, SH886, SH887, SH888, SH889, SH88z, SH89., SH890, SH891, SH892, SH893, SH894, SH895, SH896, SH897, SH898, SH899, SH89A, SH89z, SH8z., SH9.., SH90., SH91., SH92., SH920, SH921, SH93., SH94., SH95., SH9z., SHz.., SyuD., SyuD0, SyuD1, SyuD2, SyuD3, SyuD4, SyuD5, SyuD6, SyuD7, SyuD8, SyuD9, SyuDA, SyuDB, SyuDC, SyuDD, SyuDE, T410., T4100, T4101, T4102, T4103, T4104, T4105, T4106, T410y, T410z, TD05., TD050, TD051, TD052, TD053, TD054, TD055, TD056, TD057, TD058, TD059, TD05A, TD05z, TD15., TD150, TD151, TD152, TD153, TD154, TD155, TD156, TD157, TD158, TD159, TD15A, TD15B, TD15z, TD3.., TD30., TD300, TD301, TD302, TD303, TD304, TD305, TD306, TD307, TD30z, TD31., TD310, TD311, TD312, TD313, TD314, TD315, TD316, TD317, TD31z, TD32., TD320, TD321, TD322, TD32z, TD3y., TD3y0, TD3y1, TD3y2, TD3y3, TD3y4, TD3y5, TD3y6, TD3y7, TD3y8, TD3y9, TD3yz, TD3z., TD4.., TD40., TD41., TD42., TD43., TD44., TD45., TD46., TD4z., TG300, TG8.., TG80., TG800, TG801, TG802, TG803, TG804, TG805, TG806, TG807, TG808, TG809, TG80A, TG80B, TG80C, TG80D, TG80y, TG80z, TG81., TG810, TG811, TG812, TG813, TG814, TG815, TG81y, TG81z, TG8y., TG8y0, TG8y1, TG8y2, TG8y3, TG8y4, TG8yz, TG8z., TKx1., TKx2., TKx7., TL1.., TL10., TL11., TL1z., TLx0z, TLx3., TN81., TN82., TN87., U165., U1650, U1651, U1652, U1653, U1654, U1655, U1656, U1657, U165y, U165z, U166., U1660, U1661, U1662, U1663, U1664, U1665, U1666, U1667, U166y, U166z, U17.., U170., U1700, U1701, U1702, U1703, U1704, U1705, U1706, U1707, U170y, U170z, U171., U1710, U1711, U1712, U1713, U1714, U1715, U1716, U1717, U171y, U171z, U172., U1720, U1721, U1722, U1723, U1724, U1725, U1726, U1727, U172y, U172z, U173., U1730, U1731, U1732, U1733, U1734, U1735, U1736, U1737, U173y, U173z, U174., U1740, U1741, U1742, U1743, U1744, U1745, U1746, U1747, U174y, U174z, U175., U1750, U1751, U1752, U1753, U1754, U1755, U1756, U1757, U175y, U175z, U176., U1760, U1761, U1762, U1763, U1764, U1765, U1766, U1767, U176y, U176z, U177., U1770, U1771, U1772, U1773, U1774, U1775, U1776, U1777, U177y, U177z, U178., U1780, U1781, U1782, U1783, U1784, U1785, U1786, U1787, U178y, U178z, U17y., U17y0, U17y1, U17y2, U17y3, U17y4, U17y5, U17y6, U17y7, U17yy, U17yz, U28.., U280., U281., U282., U283., U284., U285., U286., U287., U28y., U28z., U31.., U310., U311., U312., U313., U314., U315., U316., U317., U31y., U31z., U3D.., U3D0., U3D1., U3D2., U3D3., U3D4., U3D5., U3D6., U3D7., U3Dy., U3Dz., U48.., U480., U481., U482., U483., U484., U485., U486., U487., U48y., U48z. |
| 9 Injury to muscle and tendon, blood vessels and nerves | SB..., SB0.., SB00., SB000, SB001, SB002, SB003, SB00z, SB01., SB02., SB03., SB0y., SB0y0, SB0y1, SB0yz, SB0z., SB1.., SB10., SB11., SB12., SB120, SB121, SB13., SB14., SB15., SB150, SB151, SB16., SB160, SB161, SB162, SB16z, SB1y., SB1y0, SB1y1, SB1y2, SB1y3, SB1y4, SB1y5, SB1yx, SB1yy, SB1yz, SB1z., SB2.., SB20., SB21., SB210, SB211, SB21z, SB22., SB220, SB221, SB222, SB223, SB224, SB225, SB226, SB227, SB22z, SB23., SB230, SB231, SB232, SB233, SB234, SB235, SB23z, SB24., SB240, SB241, SB242, SB243, SB24z, SB25., SB250, SB251, SB252, SB253, SB254, SB255, SB256, SB25z, SB2y., SB2y0, SB2y1, SB2yx, SB2yz, SB2z., SB3.., SB30., SB300, SB301, SB302, SB303, SB304, SB30X, SB30z, SB31., SB310, SB311, SB32., SB320, SB321, SB322, SB33., SB330, SB331, SB332, SB34., SB340, SB341, SB342, SB343, SB35., SB350, SB351, SB352, SB353, SB354, SB355, SB36., SB360, SB3W., SB3x., SB3X., SB3x0, SB3y., SB3y0, SB3z., SB4.., SB40., SB400, SB401, SB41., SB410, SB411, SB42., SB420, SB421, SB43., SB430, SB431, SB43z, SB44., SB440, SB441, SB442, SB443, SB444, SB445, SB446, SB44z, SB45., SB450, SB451, SB452, SB453, SB454, SB455, SB456, SB457, SB458, SB45z, SB46., SB460, SB461, SB47., SB470, SB471, SB472, SB473, SB48., SB480, SB481, SB482, SB483, SB4W., SB4x., SB4X., SB4x0, SB4y., SB4y0, SB4y1, SB4z., SBz.., SJ..., SJ14., SJ15., SJ16., SJ160, SJ161, SJ17., SJ18., SJ1y., SJ1y0, SJ1y1, SJ1y2, SJ1yz, SJ1z., SJ2.., SJ20., SJ200, SJ201, SJ202, SJ203, SJ204, SJ205, SJ206, SJ207, SJ208, SJ209, SJ20A, SJ20B, SJ20z, SJ21., SJ210, SJ211, SJ212, SJ213, SJ214, SJ215, SJ216, SJ217, SJ218, SJ219, SJ21A, SJ21B, SJ21z, SJ22., SJ220, SJ221, SJ222, SJ223, SJ224, SJ225, SJ23., SJ24., SJ240, SJ241, SJ2x., SJ2z., SJ3.., SJ30., SJ300, SJ301, SJ302, SJ303, SJ304, SJ305, SJ306, SJ307, SJ31., SJ310, SJ311, SJ312, SJ313, SJ314, SJ315, SJ316, SJ317, SJ318, SJ319, SJ31A, SJ31B, SJ32., SJ320, SJ321, SJ322, SJ323, SJ324, SJ33., SJ330, SJ331, SJ332, SJ333, SJ334, SJ34., SJ340, SJ341, SJ342, SJ343, SJ344, SJ345, SJ35., SJ350, SJ351, SJ3x., SJ3z., SJ4.., SJ40., SJ41., SJ410, SJ411, SJ412, SJ413, SJ414, SJ415, SJ41z, SJ42., SJ43., SJ44., SJ45., SJ4y., SJ4z., SJ5.., SJ50., SJ500, SJ501, SJ51., SJ510, SJ511, SJ512, SJ513, SJ514, SJ515, SJ52., SJ520, SJ521, SJ522, SJ523, SJ524, SJ525, SJ526, SJ527, SJ528, SJ53., SJ530, SJ531, SJ532, SJ533, SJ534, SJ54., SJ540, SJ541, SJ55., SJ56., SJ560, SJ561, SJ562, SJ563, SJ564, SJ565, SJ566, SJ57., SJ570, SJ571, SJ58., SJ580, SJ581, SJ59., SJ590, SJ591, SJ5A., SJ5A0, SJ5A1, SJ5B., SJ5B0, SJ5B1, SJ5C., SJ5C0, SJ5C1, SJ5x., SJ5X., SJ5y., SJ5y0, SJ5y1, SJ5z., SJ6.., SJ60., SJ600, SJ601, SJ61., SJ610, SJ611, SJ62., SJ620, SJ621, SJ63., SJ630, SJ631, SJ632, SJ633, SJ634, SJ635, SJ64., SJ640, SJ641, SJ642, SJ643, SJ65., SJ66., SJ660, SJ66X, SJ67., SJ670, SJ671, SJ672, SJ673, SJ674, SJ67X, SJ6W., SJ6x., SJ6X., SJ6x0, SJ6x1, SJ6x2, SJ6x3, SJ6y., SJ6z., SJ7.., SJ70., SJ71., SJ7x., SJ7x0, SJ7y., SJ7z., SJ8.., SJ80., SJ81., SJ9.., SJ90., SJA.., SJA0., SJB.., SJB0., SJB1., SJB2., SJB3., SJW.., SJX.., SJz.., SK108, SK124, SK125, SK177, SK18., SK1A., SK1B., SK1C., SK1C0, SK1C1, SK1C2, SK1C3, SK1C4, SK1C5, SK1CW, SK1CX, SK1D., SK1D0, SK1D1, SK1E., SK1E0, SK1E1, SK1E2, SK1EX, SK1F., SK1F0, SK1F1, SK1FX, SK1W., SK1X., SRy1., SRy2., Syu07, Syu0K, Syu1A, Syu1B, Syu1C, Syu2C, Syu2D, Syu2E, Syu2F, Syu39, Syu3A, Syu3B, Syu47, Syu48, Syu49, Syu4A, Syu4B, Syu4C, Syu55, Syu56, Syu57, Syu58, Syu59, Syu5A, Syu5B, Syu5C, Syu67, Syu68, Syu69, Syu6A, Syu6B, Syu6C, Syu6D, Syu6E, Syu73, Syu74, Syu75, Syu76, Syu77, Syu85, Syu86, Syu87, Syu88, Syu89, Syu8A, Syu8E, Syu97, Syu98, Syu99, Syu9A, Syu9B, Syu9C, SyuAA, SyuB4, SyuB5, SyuB6, SyuBD, SyuBE, SyuBF, SyuBJ, SyuBL, SyuBM |
| 10 Injury to internal organs | S7..., S70.., S700., S701., S702., S703., S704., S705., S706., S707., S708., S70z., S71.., S710., S7100, S7101, S7102, S7103, S710y, S710z, S711., S7110, S7111, S7112, S7113, S711y, S711z, S712., S7120, S7121, S7122, S712z, S713., S7130, S7131, S7132, S713z, S714., S7140, S7141, S715., S71z., S72.., S720., S721., S722., S7220, S7221, S7222, S7223, S7224, S722z, S723., S7230, S7231, S7232, S7233, S7234, S723z, S724., S725., S7250, S7251, S726., S727., S72x., S72y., S72z., S73.., S730., S731., S732., S7320, S7321, S732z, S733., S7330, S7331, S733z, S734., S7340, S7341, S7342, S7343, S7344, S7345, S734x, S734y, S734z, S735., S7350, S7351, S7352, S7353, S7354, S7355, S735x, S735y, S735z, S736., S737., S738., S739., S73A., S73A0, S73A1, S73B., S73x., S73x0, S73x1, S73x2, S73x3, S73x4, S73x5, S73x6, S73xy, S73xz, S73y., S73y0, S73y1, S73y2, S73y3, S73y4, S73y5, S73y6, S73yy, S73yz, S73z., S74.., S740., S7400, S7401, S7402, S7403, S7404, S740y, S740z, S741., S7410, S7411, S7412, S7413, S7414, S741y, S741z, S74z., S75.., S750., S7500, S7501, S7502, S7503, S7504, S750y, S750z, S751., S7510, S7511, S7512, S7513, S7514, S751y, S751z, S75z., S76.., S760., S7600, S7601, S7602, S7603, S760z, S761., S7610, S7611, S7612, S7613, S761z, S76z., S77.., S770., S7700, S7701, S770z, S771., S7710, S7711, S771z, S772., S773., S774., S775., S776., S777., S778., S779., S77A., S77B., S77C., S77C0, S77C1, S77v., S77v0, S77v1, S77v2, S77v3, S77v4, S77vz, S77w., S77w0, S77w1, S77w2, S77w3, S77w4, S77wz, S77x., S77y., S77z., S78.., S780., S7800, S7801, S7802, S7803, S7804, S7805, S780z, S781., S7810, S7811, S7812, S7813, S7814, S781z, S78z., S79.., S790., S791., S79z., S7A.., S7B.., S7z.., SK1x6, SRy3., Syu2J, Syu2K, Syu3C, Syu3D, Syu3E, Syu3F |
| 11 Poisoning | SL..., SL0.., SL00., SL000, SL001, SL002, SL003, SL00z, SL01., SL010, SL011, SL012, SL013, SL01z, SL02., SL020, SL021, SL02z, SL03., SL030, SL031, SL032, SL03z, SL04., SL040, SL041, SL042, SL043, SL04z, SL05., SL050, SL051, SL052, SL053, SL05z, SL06., SL060, SL061, SL062, SL063, SL06z, SL07., SL070, SL071, SL072, SL073, SL074, SL07z, SL0y., SL0z., SL1.., SL10., SL100, SL101, SL102, SL10z, SL11., SL12., SL120, SL121, SL122, SL123, SL12z, SL13., SL130, SL131, SL13z, SL14., SL140, SL141, SL142, SL143, SL144, SL145, SL14z, SL15., SL150, SL15z, SL16., SL160, SL161, SL162, SL16z, SL17., SL170, SL17z, SL1x., SL1x0, SL1x1, SL1x2, SL1x3, SL1x4, SL1xz, SL1y., SL1y0, SL1y1, SL1yz, SL1z., SL2.., SL20., SL200, SL201, SL202, SL203, SL20z, SL21., SL210, SL211, SL212, SL213, SL21z, SL22., SL220, SL221, SL222, SL223, SL22z, SL23., SL230, SL231, SL232, SL233, SL234, SL235, SL236, SL237, SL23z, SL24., SL240, SL241, SL242, SL24z, SL25., SL250, SL25z, SL26., SL27., SL270, SL271, SL272, SL273, SL27z, SL28., SL280, SL281, SL282, SL28z, SL29., SL2y., SL2z., SL3.., SL30., SL300, SL301, SL302, SL303, SL304, SL30x, SL30z, SL31., SL310, SL311, SL312, SL313, SL314, SL315, SL316, SL317, SL31z, SL32., SL33., SL34., SL340, SL34z, SL35., SL350, SL351, SL35z, SL3y., SL3y0, SL3yz, SL3z., SL4.., SL40., SL400, SL401, SL40x, SL40z, SL41., SL410, SL41z, SL42., SL420, SL421, SL422, SL423, SL424, SL42z, SL43., SL44., SL440, SL441, SL442, SL443, SL44z, SL45., SL450, SL451, SL45z, SL46., SL47., SL470, SL471, SL472, SL473, SL47z, SL4y., SL4y0, SL4y1, SL4yz, SL4z., SL5.., SL50., SL500, SL501, SL502, SL503, SL504, SL505, SL506, SL507, SL50z, SL51., SL510, SL511, SL51z, SL52., SL520, SL521, SL522, SL52z, SL53., SL530, SL531, SL53z, SL54., SL540, SL541, SL542, SL543, SL544, SL54z, SL5x., SL5x0, SL5xz, SL5y., SL5y0, SL5y1, SL5y2, SL5yz, SL5z., SL6.., SL60., SL600, SL601, SL60z, SL61., SL610, SL61z, SL62., SL620, SL621, SL62z, SL6x., SL6x0, SL6x1, SL6xz, SL6y., SL6y0, SL6y1, SL6y2, SL6y3, SL6yz, SL6z., SL7.., SL70., SL700, SL701, SL702, SL703, SL704, SL705, SL70z, SL71., SL72., SL73., SL730, SL731, SL73z, SL74., SL75., SL76., SL7y., SL7z., SL8.., SL80., SL800, SL801, SL802, SL80z, SL81., SL82., SL820, SL821, SL822, SL82z, SL83., SL830, SL831, SL832, SL83x, SL83z, SL84., SL85., SL850, SL851, SL852, SL853, SL85z, SL86., SL87., SL8z., SL9.., SL90., SL900, SL901, SL902, SL903, SL90z, SL91., SL910, SL911, SL912, SL913, SL914, SL91z, SL92., SL920, SL921, SL922, SL92z, SL93., SL94., SL940, SL941, SL942, SL943, SL944, SL945, SL946, SL94z, SL95., SL950, SL951, SL95z, SL96., SL960, SL961, SL962, SL963, SL964, SL96z, SL97., SL970, SL971, SL972, SL97z, SL9y., SL9z., SLA.., SLA0., SLA00, SLA01, SLA0z, SLA1., SLA10, SLA11, SLA12, SLA1z, SLAy., SLAz., SLB.., SLB0., SLB00, SLB01, SLB02, SLB0z, SLB1., SLB10, SLB11, SLB12, SLB13, SLB14, SLB1z, SLB2., SLB20, SLB21, SLB2z, SLB3., SLB30, SLB31, SLB3z, SLBz., SLC.., SLC0., SLC00, SLC01, SLC02, SLC03, SLC04, SLC0z, SLC1., SLC10, SLC11, SLC12, SLC1z, SLC2., SLC20, SLC21, SLC2z, SLC3., SLC30, SLC3z, SLC4., SLC40, SLC41, SLC42, SLC4z, SLC5., SLC50, SLC51, SLC52, SLC5z, SLC6., SLC60, SLC61, SLC62, SLC63, SLC64, SLC6z, SLC7., SLC70, SLC71, SLC7z, SLC8., SLC80, SLC81, SLC8z, SLC9., SLCz., SLD.., SLD0., SLD00, SLD01, SLD02, SLD0z, SLD1., SLD10, SLD11, SLD12, SLD1z, SLD2., SLD20, SLD2z, SLD3., SLD30, SLD31, SLD3z, SLD4., SLD40, SLD41, SLD42, SLD4z, SLD5., SLD50, SLD51, SLD5z, SLD6., SLDy., SLDz., SLE.., SLE0., SLE00, SLE01, SLE02, SLE0z, SLE1., SLE10, SLE11, SLE1z, SLE2., SLE20, SLE2z, SLE3., SLE30, SLE31, SLE3z, SLE4., SLE40, SLE41, SLE4z, SLE5., SLE6., SLE7., SLE70, SLE71, SLE72, SLE7z, SLEz., SLF.., SLF0., SLF00, SLF01, SLF02, SLF0z, SLF1., SLF10, SLF11, SLF1z, SLF2., SLF3., SLF4., SLF40, SLF41, SLF4z, SLF5., SLF50, SLF51, SLF52, SLF5z, SLF6., SLF7., SLF70, SLF71, SLF7z, SLFy., SLFz., SLG.., SLG0., SLG1., SLG2., SLG3., SLG4., SLG5., SLG50, SLG5z, SLG6., SLG7., SLGx., SLGx0, SLGxz, SLGz., SLH.., SLH0., SLH00, SLH0z, SLH1., SLH2., SLH3., SLH4., SLHy., SLHy0, SLHy1, SLHyz, SLHz., SLJ.., SLJ0., SLJ1., SLJ10, SLJ11, SLJ1z, SLJ2., SLJ3., SLJ4., SLJ5., SLJ6., SLJx., SLJy., SLJz., SLK.., SLK0., SLK1., SLK2., SLK3., SLK4., SLK5., SLK6., SLK60, SLK6z, SLK7., SLK8., SLKz., SLX.., SLz.., SM..., SM0.., SM00., SM000, SM001, SM002, SM00z, SM01., SM010, SM011, SM01z, SM02., SM020, SM021, SM022, SM02z, SM03., SM030, SM031, SM032, SM03z, SM0y., SM0z., SM1.., SM10., SM11., SM12., SM13., SM14., SM15., SM1z., SM2.., SM20., SM21., SM22., SM23., SM230, SM231, SM232, SM23z, SM24., SM2y., SM2y0, SM2y1, SM2yz, SM2z., SM3.., SM30., SM300, SM301, SM30z, SM31., SM310, SM311, SM312, SM31z, SM32., SM320, SM321, SM322, SM32z, SM3z., SM4.., SM40., SM400, SM401, SM40z, SM41., SM410, SM411, SM41z, SM4y., SM4z., SM5.., SM50., SM51., SM52., SM53., SM54., SM55., SM56., SM57., SM58., SM5y., SM5y0, SM5y1, SM5y2, SM5y3, SM5yz, SM5z., SM6.., SM7.., SM70., SM700, SM701, SM70z, SM71., SM72., SM720, SM721, SM72z, SM73., SM74., SM75., SM750, SM751, SM752, SM75z, SM76., SM77., SM78., SM79., SM7A., SM7y., SM7y0, SM7y1, SM7y2, SM7yz, SM7z., SM8.., SM80., SM800, SM801, SM80W, SM80X, SM81., SM82., SM8y., SM8z., SM9.., SM90., SM900, SM901, SM90z, SM91., SM92., SM920, SM921, SM922, SM923, SM92z, SM93., SM930, SM931, SM932, SM933, SM934, SM935, SM93z, SM94., SM95., SM950, SM951, SM952, SM953, SM954, SM955, SM956, SM95z, SM96., SM97., SM98., SM9A., SM9B., SM9B0, SM9B1, SM9C., SM9X., SM9y., SM9z., SMB.., SMC.., SMX.., SMz.., SyuF., SyuF0, SyuF1, SyuF2, SyuF3, SyuF4, SyuF5, SyuF6, SyuF7, SyuF8, SyuF9, SyuFa, SyuFA, SyuFb, SyuFB, SyuFc, SyuFC, SyuFd, SyuFD, SyuFe, SyuFE, SyuFF, SyuFG, SyuFH, SyuFJ, SyuFK, SyuFL, SyuFM, SyuFN, SyuFP, SyuFQ, SyuFR, SyuFS, SyuFT, SyuFU, SyuFV, SyuFW, SyuFX, SyuFY, SyuFZ, SyuG., SyuG0, SyuG1, SyuG2, SyuG3, SyuG4, SyuG5, SyuG6, SyuG7, SyuG8, SyuG9, SyuGA, SyuGB, SyuGC, SyuGD, SyuGE, SyuGF, SyuGG, SyuGH, SyuGJ, SyuGK, SyuGL, SyuGM, T180., T1800, T1801, T1802, T1803, T1804, T1805, T1806, T1807, T180y, T180z, T250., T2500, T2501, T2502, T2503, T2504, T2505, T2506, T2507, T250y, T250z, T470., T4700, T4701, T4702, T4703, T4704, T4705, T4706, T470y, T470z, T545., T5450, T5451, T5452, T5453, T5454, T5455, T5456, T5457, T5458, T545z, T8..., T80.., T800., T801., T802., T8020, T8021, T8022, T8023, T802z, T803., T8030, T803z, T804., T8040, T8041, T8042, T804z, T805., T8050, T8051, T805z, T806., T8060, T8061, T8062, T8063, T8064, T806z, T807., T8070, T807z, T80y., T80y0, T80yz, T80z., T81.., T810., T811., T812., T813., T814., T815., T81z., T82.., T820., T821., T822., T8220, T8221, T8222, T822z, T823., T824., T825., T82y., T82z., T83.., T830., T8300, T8301, T8302, T8303, T830z, T831., T8310, T8311, T8312, T831z, T832., T8320, T8321, T8322, T8323, T8324, T8325, T832z, T83y., T83y0, T83y1, T83yz, T83z., T84.., T840., T8400, T8401, T8402, T840z, T841., T8410, T8411, T8412, T8413, T8414, T8415, T841z, T842., T8420, T8421, T842z, T843., T8430, T8431, T843z, T84z., T85.., T850., T8500, T8501, T8502, T8503, T8504, T850z, T851., T8510, T8511, T8512, T8513, T8514, T851z, T852., T8520, T8521, T8522, T8523, T852z, T853., T8530, T8531, T8532, T853z, T854., T8540, T8541, T8542, T8543, T854z, T855., T8550, T8551, T855z, T856., T8560, T8561, T856z, T85y., T85z., T86.., T87.., T88.., T880., T881., T882., T883., T884., T885., T886., T887., T8870, T8871, T8872, T8873, T8874, T887z, T88y., T88y0, T88yz, T88z., T8z.., T9..., T90.., T900., T901., T9010, T9011, T9012, T9013, T901z, T902., T9020, T9021, T902z, T903., T9030, T9031, T9032, T9033, T903z, T904., T90y., T90z., T91.., T910., T911., T912., T913., T9130, T913z, T914., T915., T916., T9160, T9161, T9162, T9163, T916z, T91z., T92.., T920., T9200, T9201, T9202, T920z, T921., T9210, T9211, T9212, T9213, T921z, T922., T923., T9230, T923z, T924., T9240, T924z, T92z., T93.., T930., T9300, T9301, T9302, T9303, T9304, T9305, T930z, T931., T9310, T9311, T9312, T9313, T9314, T9315, T9316, T9317, T9318, T931z, T932., T9320, T9321, T9322, T932z, T933., T934., T9340, T934z, T935., T9350, T9351, T9352, T9353, T9354, T9355, T935z, T936., T9360, T9361, T936z, T937., T9370, T9371, T9372, T9373, T9374, T937z, T938., T9380, T9381, T9382, T938z, T93z., T94.., T940., T9400, T940z, T941., T9410, T9411, T9412, T941z, T942., T9420, T9421, T942z, T94y., T94z., T95.., T950., T951., T952., T953., T9530, T9531, T953z, T954., T955., T9550, T955y, T955z, T95y., T95z., T96.., T960., T9600, T9601, T9602, T960z, T961., T9610, T9611, T9612, T961z, T962., T9620, T9621, T9622, T962z, T963., T9630, T9631, T9632, T963z, T964., T9640, T9641, T9642, T9643, T9644, T9645, T9646, T9647, T964z, T965., T9650, T9651, T965z, T966., T9660, T966y, T966z, T967., T96y., T96z., T97.., T970., T971., T972., T973., T97z., T98.., T980., T9801, T9802, T980z, T981., T9810, T9811, T9812, T9813, T9814, T981z, T982., T9820, T9821, T9822, T9823, T982z, T983., T9830, T9831, T9832, T9833, T983z, T98y., T98y0, T98y1, T98y2, T98yz, T98z., T99.., T990., T991., T992., T993., T9930, T9931, T9932, T993z, T99y., T99y0, T99y1, T99yz, T99z., T9z.., TD01., TD010, TD011, TD012, TD013, TD014, TD015, TD016, TD017, TD018, TD019, TD01A, TD01z, TD02., TD020, TD021, TD022, TD023, TD024, TD025, TD026, TD027, TD028, TD029, TD02A, TD02z, TD03., TD030, TD031, TD032, TD033, TD034, TD035, TD036, TD037, TD038, TD039, TD03A, TD03z, TD11., TD110, TD111, TD112, TD113, TD114, TD115, TD116, TD117, TD118, TD119, TD11A, TD11B, TD11z, TD12., TD120, TD121, TD122, TD123, TD124, TD125, TD126, TD127, TD128, TD129, TD12A, TD12B, TD12z, TD13., TD130, TD131, TD132, TD133, TD134, TD135, TD136, TD137, TD138, TD139, TD13A, TD13B, TD13z, TE5.., TE50., TE500, TE501, TE502, TE503, TE504, TE505, TE506, TE507, TE508, TE509, TE50A, TE50y, TE50z, TE51., TE510, TE511, TE512, TE51z, TE52., TE53., TE530, TE531, TE532, TE53z, TE54., TE540, TE541, TE54z, TE55., TE550, TE551, TE55z, TE56., TE560, TE561, TE562, TE563, TE564, TE565, TE56y, TE56z, TE57., TE5y., TE5z., TK0.., TK00., TK01., TK010, TK011, TK012, TK013, TK014, TK015, TK01z, TK02., TK03., TK04., TK05., TK06., TK07., TK08., TK0z., TK1.., TK10., TK11., TK1y., TK1z., TK2.., TK20., TK21., TK2y., TK2z., TL2.., TL20., TL21., TL22., TL2z., TM21., TM84., TN0.., TN00., TN01., TN010, TN011, TN012, TN013, TN014, TN015, TN01z, TN02., TN03., TN04., TN05., TN06., TN07., TN08., TN0z., TN1.., TN10., TN11., TN1y., TN1z., TN2.., TN20., TN21., TN2y., TN2z., U1A.., U1A0., U1A00, U1A01, U1A02, U1A03, U1A04, U1A05, U1A06, U1A07, U1A0y, U1A0z, U1A1., U1A10, U1A11, U1A12, U1A13, U1A14, U1A15, U1A16, U1A17, U1A1y, U1A1z, U1A2., U1A20, U1A21, U1A22, U1A23, U1A24, U1A25, U1A26, U1A27, U1A2y, U1A2z, U1A3., U1A30, U1A31, U1A32, U1A33, U1A34, U1A35, U1A36, U1A37, U1A3y, U1A3z, U1A4., U1A40, U1A41, U1A42, U1A43, U1A44, U1A45, U1A46, U1A47, U1A4y, U1A4z, U1A5., U1A50, U1A51, U1A52, U1A53, U1A54, U1A55, U1A56, U1A57, U1A5y, U1A5z, U1A6., U1A60, U1A61, U1A62, U1A63, U1A64, U1A65, U1A66, U1A67, U1A6y, U1A6z, U1A7., U1A70, U1A71, U1A72, U1A73, U1A74, U1A75, U1A76, U1A77, U1A7y, U1A7z, U1A8., U1A80, U1A81, U1A82, U1A83, U1A84, U1A85, U1A86, U1A87, U1A8y, U1A8z, U1A9., U1A90, U1A91, U1A92, U1A93, U1A94, U1A95, U1A96, U1A97, U1A9y, U1A9z, U1AA., U1AA0, U1AA1, U1AA2, U1AA3, U1AA4, U1AA5, U1AA6, U1AA7, U1AAy, U1AAz, U1AB., U1AB0, U1AB1, U1AB2, U1AB3, U1AB4, U1AB5, U1AB6, U1AB7, U1ABy, U1ABz, U1AC., U1AC0, U1AC1, U1AC2, U1AC3, U1AC4, U1AC5, U1AC6, U1AC7, U1ACy, U1ACz, U1AD., U1AD0, U1AD1, U1AD2, U1AD3, U1AD4, U1AD5, U1AD6, U1AD7, U1ADy, U1ADz, U1Ay., U1Ay0, U1Ay1, U1Ay2, U1Ay3, U1Ay4, U1Ay5, U1Ay6, U1Ay7, U1Ayy, U1Ayz, U20.., U200., U2000, U2001, U2002, U2003, U2004, U2005, U2006, U2007, U200y, U200z, U201., U2010, U2011, U2012, U2013, U2014, U2015, U2016, U2017, U201y, U201z, U202., U2020, U2021, U2022, U2023, U2024, U2025, U2026, U2027, U202y, U202z, U203., U2030, U2031, U2032, U2033, U2034, U2035, U2036, U2037, U203y, U203z, U204., U2040, U2041, U2042, U2043, U2044, U2045, U2046, U2047, U204y, U204z, U205., U2050, U2051, U2052, U2053, U2054, U2055, U2056, U2057, U205y, U205z, U206., U2060, U2061, U2062, U2063, U2064, U2065, U2066, U2067, U206y, U206z, U207., U2070, U2071, U2072, U2073, U2074, U2075, U2076, U2077, U207y, U207z, U208., U2080, U2081, U2082, U2083, U2084, U2085, U2086, U2087, U208y, U208z, U209., U2090, U2091, U2092, U2093, U2094, U2095, U2096, U2097, U209y, U209z, U20A., U20A0, U20A1, U20A2, U20A3, U20A4, U20A5, U20A6, U20A7, U20Ay, U20Az, U20B., U20B0, U20B1, U20B2, U20B3, U20B4, U20B5, U20B6, U20B7, U20By, U20Bz, U20C., U20C0, U20C1, U20C2, U20C3, U20C4, U20C5, U20C6, U20C7, U20Cy, U20Cz, U20y., U20y0, U20y1, U20y2, U20y3, U20y4, U20y5, U20y6, U20y7, U20yy, U20yz, U40.., U400., U4000, U4001, U4002, U4003, U4004, U4005, U4006, U4007, U400y, U400z, U401., U4010, U4011, U4012, U4013, U4014, U4015, U4016, U4017, U401y, U401z, U402., U4020, U4021, U4022, U4023, U4024, U4025, U4026, U4027, U402y, U402z, U403., U4030, U4031, U4032, U4033, U4034, U4035, U4036, U4037, U403y, U403z, U404., U4040, U4041, U4042, U4043, U4044, U4045, U4046, U4047, U404y, U404z, U405., U4050, U4051, U4052, U4053, U4054, U4055, U4056, U4057, U405y, U405z, U406., U4060, U4061, U4062, U4063, U4064, U4065, U4066, U4067, U406y, U406z, U407., U4070, U4071, U4072, U4073, U4074, U4075, U4076, U4077, U407y, U407z, U408., U4080, U4081, U4082, U4083, U4084, U4085, U4086, U4087, U408y, U408z, U409., U4090, U4091, U4092, U4093, U4094, U4095, U4096, U4097, U409y, U409z, U40A., U40A0, U40A1, U40A2, U40A3, U40A4, U40A5, U40A6, U40A7, U40Ay, U40Az, U40B., U40B0, U40B1, U40B2, U40B3, U40B4, U40B5, U40B6, U40B7, U40By, U40Bz, U40C., U40C0, U40C1, U40C2, U40C3, U40C4, U40C5, U40C6, U40C7, U40Cy, U40Cz, U40y., U40y0, U40y1, U40y2, U40y3, U40y4, U40y5, U40y6, U40y7, U40yy, U40yz |
| 98 Other | SD02., SD020, SD021, SD022, SD02z, SD03., SD030, SD031, SD032, SD03z, SD04., SD040, SD041, SD042, SD04z, SD05., SD050, SD051, SD052, SD05z, SD06., SD060, SD061, SD062, SD06z, SD07., SD070, SD071, SD072, SD07z, SD12., SD120, SD121, SD122, SD123, SD124, SD125, SD126, SD127, SD128, SD129, SD12A, SD12B, SD12C, SD12D, SD12z, SD13., SD130, SD131, SD132, SD133, SD134, SD135, SD136, SD137, SD138, SD139, SD13A, SD13B, SD13C, SD13D, SD13z, SD14., SD140, SD141, SD142, SD143, SD144, SD145, SD146, SD147, SD148, SD149, SD14A, SD14B, SD14C, SD14D, SD14z, SD15., SD150, SD151, SD152, SD153, SD154, SD155, SD156, SD157, SD158, SD159, SD15A, SD15B, SD15C, SD15D, SD15z, SD16., SD160, SD161, SD162, SD163, SD164, SD165, SD166, SD167, SD168, SD169, SD16A, SD16B, SD16C, SD16D, SD16z, SD17., SD170, SD171, SD172, SD173, SD174, SD175, SD176, SD177, SD178, SD179, SD17A, SD17B, SD17C, SD17D, SD17z, SD22., SD220, SD221, SD222, SD223, SD22z, SD23., SD230, SD231, SD232, SD233, SD23z, SD24., SD240, SD241, SD242, SD243, SD24z, SD25., SD250, SD251, SD252, SD253, SD25z, SD26., SD260, SD261, SD262, SD263, SD26z, SD27., SD270, SD271, SD272, SD273, SD27z, SD32., SD320, SD321, SD322, SD32z, SD33., SD330, SD331, SD332, SD33z, SD34., SD340, SD341, SD342, SD34z, SD35., SD350, SD351, SD352, SD35z, SD36., SD360, SD361, SD362, SD363, SD36z, SD37., SD370, SD371, SD372, SD37z, SD42., SD43., SD44., SD45., SD46., SD460, SD47., SD52., SD53., SD54., SD55., SD56., SD57., SD62., SD620, SD621, SD622, SD623, SD624, SD62z, SD63., SD630, SD631, SD632, SD633, SD634, SD63z, SD64., SD640, SD641, SD642, SD643, SD644, SD64z, SD65., SD650, SD651, SD652, SD653, SD654, SD65z, SD66., SD660, SD661, SD662, SD663, SD664, SD665, SD66z, SD67., SD670, SD671, SD672, SD673, SD674, SD67z, SD72., SD720, SD721, SD722, SD72z, SD73., SD730, SD731, SD73z, SD74., SD740, SD741, SD74z, SD75., SD750, SD751, SD75z, SD76., SD760, SD761, SD762, SD763, SD76z, SD77., SD770, SD771, SD77z, SD801, SD802, SD803, SD92., SD920, SD93., SD94., SD95., SD96., SD960, SD97., SD98., SD99., SF..., SF0.., SF00., SF000, SF001, SF00z, SF01., SF02., SF020, SF021, SF022, SF02z, SF03., SF0X., SF0z., SF1.., SF10., SF100, SF101, SF102, SF10z, SF11., SF110, SF111, SF112, SF11X, SF11z, SF12., SF13., SF1y., SF1z., SF2.., SF20., SF200, SF201, SF202, SF203, SF204, SF205, SF206, SF207, SF208, SF209, SF20A, SF20B, SF20C, SF20D, SF20y, SF20z, SF21., SF210, SF211, SF212, SF213, SF214, SF215, SF21z, SF22., SF220, SF221, SF222, SF223, SF224, SF225, SF226, SF227, SF228, SF229, SF22z, SF23., SF230, SF231, SF232, SF233, SF234, SF235, SF2y., SF2z., SF3.., SF30., SF300, SF301, SF302, SF303, SF304, SF305, SF30z, SF31., SF310, SF311, SF312, SF313, SF314, SF315, SF31z, SF32., SF320, SF321, SF322, SF323, SF324, SF325, SF32z, SF33., SF3y., SF3z., SF4.., SF40., SF4z., SFz.., SK..., SK0.., SK00., SK01., SK02., SK03., SK04., SK05., SK06., SK07., SK08., SK09., SK0y., SK0y0, SK0y1, SK0y2, SK0y3, SK0y4, SK0y5, SK0y6, SK0y7, SK0z., SK1.., SK10., SK100, SK101, SK102, SK103, SK104, SK105, SK106, SK107, SK109, SK10x, SK10y, SK10z, SK11., SK110, SK111, SK112, SK113, SK114, SK115, SK116, SK117, SK118, SK119, SK11z, SK12., SK120, SK121, SK122, SK123, SK12z, SK13., SK130, SK131, SK132, SK13z, SK14., SK15., SK150, SK151, SK152, SK153, SK15z, SK16., SK160, SK161, SK16z, SK17., SK170, SK171, SK172, SK173, SK176, SK19., SK190, SK191, SK192, SK1y., SK1z., SN41., SN410, SN412, SN413, SN41z, SN47., SN470, SN471, SN472, SN473, SN474, SN475, SN476, SN47z, SN48., SN480, SN481, SN482, SN48z, SR3.., SR30., SR31., SR32., SR4.., SR40., SR41., SR42., SR43., SR44., SRy.., Syu0B, Syu0D, Syu0F, Syu0G, Syu0H, Syu0J, Syu0L, Syu1D, Syu1E, Syu1F, Syu2G, Syu2H, Syu2L, Syu38, Syu3G, Syu3H, Syu3K, Syu4D, Syu5D, Syu5E, Syu5F, Syu6F, Syu6G, Syu6H, Syu6J, Syu6K, Syu6L, Syu78, Syu8B, Syu8C, Syu8F, Syu9D, Syu9E, Syu9F, Syu9G, SyuA3, SyuA5, SyuA6, SyuA7, SyuA9, SyuB1, SyuB2, SyuB3, SyuB7, SyuBG, SyuBH, SyuBK, T050., T0500, T0501, T0502, T0503, T050y, T050z, T40.., T400., T4000, T4001, T4002, T4003, T4004, T4005, T4006, T400y, T400z, T401., T4010, T4011, T4012, T4013, T4014, T4015, T4016, T401y, T401z, T402., T4020, T4021, T4022, T4023, T4024, T4025, T4026, T402y, T402z, T403., T4030, T4031, T4032, T4033, T4034, T4035, T4036, T403y, T403z, T404., T4040, T4041, T4042, T4043, T4044, T4045, T4046, T404y, T404z, T405., T4050, T4051, T4052, T4053, T4054, T4055, T4056, T405y, T405z, T406., T4060, T4061, T4062, T4063, T4064, T4065, T4066, T406y, T406z, T40z., T40z0, T40z1, T40z2, T40z3, T40z4, T40z5, T40z6, T40zy, T40zz, T411., T4110, T4111, T4112, T4113, T4114, T4115, T4116, T411y, T411z, T412., T4120, T4121, T4122, T4123, T4124, T4125, T4126, T412y, T412z, T42.., T420., T4200, T4201, T4202, T4203, T4204, T4205, T4206, T420y, T420z, T421., T4210, T4211, T4212, T4213, T4214, T4215, T4216, T421y, T421z, T422., T4220, T4221, T4222, T4223, T4224, T4225, T4226, T422y, T422z, T423., T4230, T4231, T4232, T4233, T4234, T4235, T4236, T423y, T423z, T42z., T42z0, T42z1, T42z2, T42z3, T42z4, T42z5, T42z6, T42zy, T42zz, T472., T4720, T4721, T4722, T4723, T4724, T4725, T4726, T472y, T472z, T473., T4730, T4731, T4732, T4733, T4734, T4735, T4736, T473y, T473z, T474., T4740, T4741, T4742, T4743, T4744, T4745, T4746, T474y, T474z, T475., T4750, T4751, T4752, T4753, T4754, T4755, T4756, T475y, T475z, TD04., TD040, TD041, TD042, TD043, TD044, TD045, TD046, TD047, TD048, TD049, TD04A, TD04z, TD14., TD140, TD141, TD142, TD143, TD144, TD145, TD146, TD147, TD148, TD149, TD14A, TD14B, TD14z, TE6.., TE60., TE61., TE62., TE63., TE630, TE631, TE632, TE633, TE63z, TE64., TE640, TE64z, TE6y., TE6y0, TE6y1, TE6y2, TE6y3, TE6y4, TE6y5, TE6y6, TE6y7, TE6y8, TE6yz, TE6z., TE7.., TF0.., TF00., TF01., TF010, TF011, TF012, TF01z, TF02., TF020, TF021, TF022, TF023, TF024, TF025, TF026, TF02z, TF03., TF030, TF031, TF032, TF033, TF034, TF035, TF03z, TF04., TF0y., TF0y0, TF0y1, TF0yz, TF0z., TF1.., TF10., TF100, TF101, TF102, TF10z, TF11., TF110, TF111, TF112, TF11z, TF12., TF120, TF121, TF122, TF12z, TF13., TF130, TF131, TF132, TF133, TF13z, TF1z., TF3.., TF30., TF300, TF301, TF30z, TF31., TF32., TF320, TF321, TF32z, TF33., TF330, TF33z, TF3y., TF3y0, TF3yz, TF3z., TF3z0, TF3z1, TF3z2, TF3zz, TG303, TG305, TG30A, TG9.., TG90., TG91., TG910, TG911, TG912, TG91z, TG92., TG920, TG921, TG922, TG923, TG92z, TG9y., TG9y0, TG9y1, TG9y2, TG9y3, TG9y4, TG9yz, TG9z., TG9z0, TG9z1, TG9z2, TG9zz, TGyy., TGyz2, TGyz6, TK3.., TK30., TK31., TK3y., TK3z., TK4.., TKx3., TKx4., TL3.., TL30., TL31., TL32., TL33., TL3z., TL4.., TM2.., TM20., TM22., TM2z., TM80., TM81., TM82., TM83., TN3.., TN30., TN31., TN3y., TN3z., TN4.., TN84., TP43., TP52., TP7.., TP70., TP71., TP72., TP720, TP721, TP722, TP72z, TP7y., TP7z., U090., U0900, U0901, U0902, U0903, U0904, U0905, U0906, U0907, U0908, U090z, U092., U0920, U0921, U0922, U0923, U0924, U0925, U0926, U0927, U0928, U092z, U11S., U11S0, U11S1, U11S2, U11S3, U11S4, U11S5, U11S6, U11S7, U11Sy, U11Sz, U127., U1270, U1271, U1272, U1273, U1274, U1275, U1276, U1277, U127y, U127z, U12A., U12A0, U12A1, U12A2, U12A3, U12A4, U12A5, U12A6, U12A7, U12Ay, U12Az, U13.., U130., U1300, U1301, U1302, U1303, U1304, U1305, U1306, U1307, U130y, U130z, U131., U1310, U1311, U1312, U1313, U1314, U1315, U1316, U1317, U131y, U131z, U132., U1320, U1321, U1322, U1323, U1324, U1325, U1326, U1327, U132y, U132z, U133., U1330, U1331, U1332, U1333, U1334, U1335, U1336, U1337, U133y, U133z, U134., U1340, U1341, U1342, U1343, U1344, U1345, U1346, U1347, U134y, U134z, U135., U1350, U1351, U1352, U1353, U1354, U1355, U1356, U1357, U135y, U135z, U13y., U13y0, U13y1, U13y2, U13y3, U13y4, U13y5, U13y6, U13y7, U13yy, U13yz, U13z., U13z0, U13z1, U13z2, U13z3, U13z4, U13z5, U13z6, U13z7, U13zy, U13zz, U14.., U140., U1400, U1401, U1402, U1403, U1404, U1405, U1406, U1407, U140y, U140z, U141., U1410, U1412, U1413, U1414, U1415, U1416, U1417, U1418, U141y, U141z, U142., U1420, U1421, U1422, U1423, U1424, U1425, U1426, U1427, U142y, U142z, U143., U1430, U1431, U1432, U1433, U1434, U1435, U1436, U1437, U143y, U143z, U144., U1440, U1441, U1442, U1443, U1444, U1445, U1446, U1447, U144y, U144z, U145., U1450, U1451, U1452, U1453, U1454, U1455, U1456, U1457, U145y, U145z, U146., U1460, U1461, U1462, U1463, U1464, U1465, U1466, U1467, U1468, U146z, U14y., U14y0, U14y1, U14y2, U14y3, U14y4, U14y5, U14y6, U14y7, U14yy, U14yz, U14z., U14z0, U14z1, U14z2, U14z3, U14z4, U14z5, U14z6, U14z7, U14zy, U14zz, U150., U1500, U1501, U1502, U1503, U1504, U1505, U1506, U1507, U150y, U150z, U151., U1510, U1511, U1512, U1513, U1514, U1515, U1516, U1517, U151y, U151z, U152., U1520, U1521, U1522, U1523, U1524, U1525, U1526, U1527, U152y, U152z, U21.., U210., U211., U212., U213., U214., U215., U216., U217., U21y., U21z., U22.., U220., U221., U222., U223., U224., U225., U226., U227., U22y., U22z., U33.., U330., U331., U332., U333., U334., U335., U336., U337., U33y., U33z., U36.., U360., U361., U362., U363., U364., U365., U366., U367., U36y., U36z., U37.., U370., U371., U372., U373., U374., U375., U376., U377., U37y., U37z., U41.., U410., U411., U412., U413., U414., U415., U416., U417., U41y., U41z., U42.., U420., U421., U422., U423., U424., U425., U426., U427., U42y., U42z., U502. |
| 99 Unknown | S...., SK133, SK140, SK154, SK174, SK175, SK17z, SKz.., SN552, SN553, SN555, SN560, SN561, SN562, SN563, SN564, SR..., SRz.., Sy..., Syu.., Syu0., Syu0C, Syu0E, Syu1., Syu19, Syu2., Syu2B, Syu3., Syu3L, Syu4., Syu4E, Syu5., Syu5G, Syu6., Syu6M, Syu7., Syu8., Syu9., SyuA., SyuB., SyuB8, SyuBN, Sz..., T...., T0..., T00.., T000., T0000, T0001, T0002, T0003, T000y, T000z, T001., T0010, T0011, T0012, T0013, T001y, T001z, T00z., T00z0, T00z1, T00z2, T00z3, T00zy, T00zz, T01.., T010., T0100, T0101, T0102, T0103, T010y, T010z, T011., T0110, T0111, T0112, T0113, T011y, T011z, T012., T0120, T0121, T0122, T0123, T012y, T012z, T013., T0130, T0131, T0132, T0133, T013y, T013z, T014., T0140, T0141, T0142, T0143, T014y, T014z, T015., T0150, T0151, T0152, T0153, T015y, T015z, T01x., T01x0, T01x1, T01x2, T01x3, T01xy, T01xz, T01y., T01y0, T01y1, T01y2, T01y3, T01yy, T01yz, T01z., T01z0, T01z1, T01z2, T01z3, T01zy, T01zz, T02.., T020., T0200, T0201, T0202, T0203, T020y, T020z, T021., T0210, T0211, T0212, T0213, T021y, T021z, T02z., T02z0, T02z1, T02z2, T02z3, T02zy, T02zz, T03.., T030., T0300, T0301, T0302, T0303, T030y, T030z, T031., T0310, T0311, T0312, T0313, T031y, T031z, T032., T0320, T0321, T0322, T0323, T032y, T032z, T03z., T03z0, T03z1, T03z2, T03z3, T03zy, T03zz, T04.., T040., T0400, T0401, T040y, T040z, T041., T0410, T0411, T0412, T0413, T041y, T041z, T042., T0420, T0421, T042y, T042z, T04z., T04z0, T04z1, T04z2, T04z3, T04zy, T04zz, T05.., T051., T0510, T0511, T0512, T0513, T051y, T051z, T052., T0520, T0521, T0522, T0523, T052y, T052z, T053., T0530, T0531, T0532, T0533, T053y, T053z, T05z., T05z0, T05z1, T05z2, T05z3, T05zy, T05zz, T0x.., T0x0., T0x00, T0x01, T0x0y, T0x0z, T0x1., T0x10, T0x11, T0x12, T0x13, T0x1y, T0x1z, T0x2., T0x20, T0x21, T0x22, T0x23, T0x2y, T0x2z, T0x3., T0x30, T0x31, T0x32, T0x33, T0x3y, T0x3z, T0xz., T0xz0, T0xz1, T0xz2, T0xz3, T0xzy, T0xzz, T0y.., T0y0., T0y00, T0y01, T0y02, T0y03, T0y0y, T0y0z, T0y1., T0y10, T0y11, T0y12, T0y13, T0y1y, T0y1z, T0yz., T0yz0, T0yz1, T0yz2, T0yz3, T0yzy, T0yzz, T0z.., T1..., T10.., T100., T1000, T1001, T1002, T1003, T1004, T1005, T1006, T1007, T100y, T100z, T10z., T10z0, T10z1, T10z2, T10z3, T10z4, T10z5, T10z6, T10z7, T10zy, T10zz, T11.., T110., T1100, T1101, T1102, T1103, T1104, T1105, T1106, T1107, T110y, T110z, T11z., T11z0, T11z1, T11z2, T11z3, T11z4, T11z5, T11z6, T11z7, T11zy, T11zz, T12.., T120., T1200, T1201, T1202, T1203, T1204, T1205, T1206, T1207, T120y, T120z, T121., T1210, T1211, T1212, T1213, T1214, T1215, T1216, T1217, T121y, T121z, T122., T1220, T1221, T1222, T1223, T1224, T1225, T1226, T1227, T122y, T122z, T123., T1230, T1231, T1232, T1233, T1234, T1235, T1236, T1237, T123y, T123z, T124., T1240, T1241, T1242, T1243, T1244, T1245, T1246, T1247, T124y, T124z, T12z., T12z0, T12z1, T12z2, T12z3, T12z4, T12z5, T12z6, T12z7, T12zy, T12zz, T13.., T130., T1300, T1301, T1302, T1303, T1304, T1305, T1306, T1307, T130y, T130z, T131., T1310, T1311, T1312, T1313, T1314, T1315, T1316, T1317, T131y, T131z, T132., T1320, T1321, T1322, T1323, T1324, T1325, T1326, T1327, T132y, T132z, T133., T1330, T1331, T1332, T1333, T1334, T1335, T1336, T1337, T133y, T133z, T13z., T13z0, T13z1, T13z2, T13z3, T13z4, T13z5, T13z6, T13z7, T13zy, T13zz, T14.., T140., T1400, T1401, T1402, T1403, T1404, T1405, T1406, T1407, T140y, T140z, T141., T1410, T1411, T1412, T1413, T1414, T1415, T1416, T1417, T141y, T141z, T142., T1420, T1421, T1422, T1423, T1424, T1425, T1426, T1427, T142y, T142z, T14z., T14z0, T14z1, T14z2, T14z3, T14z4, T14z5, T14z6, T14z7, T14zy, T14zz, T15.., T150., T1500, T1501, T1502, T1503, T1504, T1505, T1506, T1507, T150y, T150z, T151., T1510, T1511, T1512, T1513, T1514, T1515, T1516, T1517, T151y, T151z, T152., T1520, T1521, T1522, T1523, T1524, T1525, T1526, T1527, T152y, T152z, T153., T1530, T1531, T1532, T1533, T1534, T1535, T1536, T1537, T153y, T153z, T154., T1540, T1541, T1542, T1543, T1544, T1545, T1546, T1547, T154y, T154z, T155., T1550, T1551, T1552, T1553, T1554, T1555, T1556, T1557, T155y, T155z, T156., T1560, T1561, T1562, T1563, T1564, T1565, T1566, T1567, T156y, T156z, T15z., T15z0, T15z1, T15z2, T15z3, T15z4, T15z5, T15z6, T15z7, T15zy, T15zz, T16.., T160., T1600, T1601, T1602, T1603, T1604, T1605, T1606, T1607, T160y, T160z, T161., T1610, T1611, T1612, T1613, T1614, T1615, T1616, T1617, T161y, T161z, T162., T1620, T1621, T1622, T1623, T1624, T1625, T1626, T1627, T162y, T162z, T163., T1630, T1631, T1632, T1633, T1634, T1635, T1636, T1637, T163y, T163z, T164., T1640, T1641, T1642, T1643, T1644, T1645, T1646, T1647, T164y, T164z, T165., T1650, T1651, T1652, T1653, T1654, T1655, T1656, T1657, T165y, T165z, T166., T1660, T1661, T1662, T1663, T1664, T1665, T1666, T1667, T166y, T166z, T16z., T16z0, T16z1, T16z2, T16z3, T16z4, T16z5, T16z6, T16z7, T16zy, T16zz, T17.., T170., T1700, T1701, T1702, T1703, T1704, T1705, T1706, T1707, T170y, T170z, T171., T1710, T1711, T1712, T1713, T1714, T1715, T1716, T1717, T171y, T171z, T172., T1720, T1721, T1722, T1723, T1724, T1725, T1726, T1727, T172y, T172z, T173., T1730, T1731, T1732, T1733, T1734, T1735, T1736, T1737, T173y, T173z, T17y., T17y0, T17y1, T17y2, T17y3, T17y4, T17y5, T17y6, T17y7, T17yy, T17yz, T17z., T17z0, T17z1, T17z2, T17z3, T17z4, T17z5, T17z6, T17z7, T17zy, T17zz, T18.., T181., T1810, T1811, T1812, T1813, T1814, T1815, T1816, T1817, T181y, T181z, T182., T1820, T1821, T1822, T1823, T1824, T1825, T1826, T1827, T182y, T182z, T183., T1830, T1831, T1832, T1833, T1834, T1835, T1836, T1837, T183y, T183z, T184., T1840, T1841, T1842, T1843, T1844, T1845, T1846, T1847, T184y, T184z, T185., T1850, T1851, T1852, T1853, T1854, T1855, T1856, T1857, T185y, T185z, T186., T1860, T1861, T1862, T1863, T1864, T1865, T1866, T1867, T186y, T186z, T187., T1870, T1871, T1872, T1873, T1874, T1875, T1876, T1877, T187y, T187z, T188., T1880, T1881, T1882, T1883, T1884, T1885, T1886, T1887, T188y, T188z, T189., T1890, T1891, T1892, T1893, T1894, T1895, T1896, T1897, T189y, T189z, T18z., T18z0, T18z1, T18z2, T18z3, T18z4, T18z5, T18z6, T18z7, T18zy, T18zz, T19.., T19z., T19z0, T19z1, T19z2, T19z3, T19z4, T19z5, T19z6, T19z7, T19zy, T19zz, T1z.., T2..., T20.., T200., T2000, T2001, T2002, T2003, T2004, T2005, T2006, T2007, T200y, T200z, T201., T2010, T2011, T2012, T2013, T2014, T2015, T2016, T2017, T201y, T201z, T202., T2020, T2021, T2022, T2023, T2024, T2025, T2026, T2027, T202y, T202z, T203., T2030, T2031, T2032, T2033, T2034, T2035, T2036, T2037, T203y, T203z, T204., T2040, T2041, T2042, T2043, T2044, T2045, T2046, T2047, T204y, T204z, T205., T2050, T2051, T2052, T2053, T2054, T2055, T2056, T2057, T205y, T205z, T20z., T20z0, T20z1, T20z2, T20z3, T20z4, T20z5, T20z6, T20z7, T20zy, T20zz, T21.., T210., T2100, T2101, T2102, T2103, T2104, T2105, T2106, T2107, T210y, T210z, T211., T2110, T2111, T2112, T2113, T2114, T2115, T2116, T2117, T211y, T211z, T212., T2120, T2121, T2122, T2123, T2124, T2125, T2126, T2127, T212y, T212z, T213., T2130, T2131, T2132, T2133, T2134, T2135, T2136, T2137, T213y, T213z, T214., T2140, T2141, T2142, T2143, T2144, T2145, T2146, T2147, T214y, T214z, T215., T2150, T2151, T2152, T2153, T2154, T2155, T2156, T2157, T215y, T215z, T216., T2160, T2161, T2162, T2163, T2164, T2165, T2166, T2167, T216y, T216z, T217., T2170, T2171, T2172, T2173, T2174, T2175, T2176, T2177, T217y, T217z, T218., T2180, T2181, T2182, T2183, T2184, T2185, T2186, T2187, T218y, T218z, T21z., T21z0, T21z1, T21z2, T21z3, T21z4, T21z5, T21z6, T21z7, T21zy, T21zz, T22.., T220., T2200, T2201, T2202, T2203, T2204, T2205, T2206, T2207, T220y, T220z, T221., T2210, T2211, T2212, T2213, T2214, T2215, T2216, T2217, T221y, T221z, T222., T2220, T2221, T2222, T2223, T2224, T2225, T2226, T2227, T222y, T222z, T223., T2230, T2231, T2232, T2233, T2234, T2235, T2236, T2237, T223y, T223z, T224., T2240, T2241, T2242, T2243, T2244, T2245, T2246, T2247, T224y, T224z, T22z., T22z0, T22z1, T22z2, T22z3, T22z4, T22z5, T22z6, T22z7, T22zy, T22zz, T23.., T23z., T23z0, T23z1, T23z2, T23z3, T23z4, T23z5, T23z6, T23z7, T23zy, T23zz, T24.., T240., T2400, T2401, T2402, T2403, T2404, T2405, T2406, T2407, T240y, T240z, T241., T2410, T2411, T2412, T2413, T2414, T2415, T2416, T2417, T241y, T241z, T242., T2420, T2421, T2422, T2423, T2424, T2425, T2426, T2427, T242y, T242z, T24z., T24z0, T24z1, T24z2, T24z3, T24z4, T24z5, T24z6, T24z7, T24zy, T24zz, T25.., T251., T2510, T2511, T2512, T2513, T2514, T2515, T2516, T2517, T251y, T251z, T252., T2520, T2521, T2522, T2523, T2524, T2525, T2526, T2527, T252y, T252z, T253., T2530, T2531, T2532, T2533, T2534, T2535, T2536, T2537, T253y, T253z, T254., T2540, T2541, T2542, T2543, T2544, T2545, T2546, T2547, T254y, T254z, T255., T2550, T2551, T2552, T2553, T2554, T2555, T2556, T2557, T255y, T255z, T256., T2560, T2561, T2562, T2563, T2564, T2565, T2566, T2567, T256y, T256z, T257., T2570, T2571, T2572, T2573, T2574, T2575, T2576, T2577, T257y, T257z, T258., T2580, T2581, T2582, T2583, T2584, T2585, T2586, T2587, T258y, T258z, T259., T2590, T2591, T2592, T2593, T2594, T2595, T2596, T2597, T259y, T259z, T25A., T25A0, T25A1, T25A2, T25A3, T25A4, T25A5, T25A6, T25A7, T25Ay, T25Az, T25B., T25B0, T25B1, T25B2, T25B3, T25B4, T25B5, T25B6, T25B7, T25By, T25Bz, T25z., T25z0, T25z1, T25z2, T25z3, T25z4, T25z5, T25z6, T25z7, T25zy, T25zz, T2z.., T3..., T30.., T300., T3000, T3001, T3002, T3003, T3004, T300y, T300z, T301., T3010, T3011, T3012, T3013, T3014, T301y, T301z, T302., T3020, T3021, T3022, T3023, T3024, T302y, T302z, T303., T3030, T3031, T3032, T3033, T3034, T303y, T303z, T304., T3040, T3041, T3042, T3043, T3044, T304y, T304z, T305., T3050, T3051, T3052, T3053, T3054, T305y, T305z, T306., T3060, T3061, T3062, T3063, T3064, T306y, T306z, T307., T3070, T3071, T3072, T3073, T3074, T307y, T307z, T308., T3080, T3081, T3082, T3083, T3084, T308y, T308z, T30z., T30z0, T30z1, T30z2, T30z3, T30z4, T30zy, T30zz, T31.., T310., T3100, T3101, T3102, T3103, T310y, T310z, T311., T3110, T3111, T3112, T3113, T311y, T311z, T312., T3120, T3121, T3122, T3123, T312y, T312z, T313., T3130, T3131, T3132, T3133, T313y, T313z, T314., T3140, T3141, T3142, T3143, T314y, T314z, T315., T3150, T3151, T3152, T3153, T315y, T315z, T316., T3160, T3161, T3162, T3163, T316y, T316z, T317., T3170, T3171, T3172, T3173, T317y, T317z, T318., T3180, T3181, T3182, T3183, T318y, T318z, T319., T3190, T3191, T3192, T3193, T319y, T319z, T31z., T31z0, T31z1, T31z2, T31z3, T31zy, T31zz, T32.., T320., T3200, T3201, T3202, T320y, T320z, T321., T3210, T3211, T3212, T321y, T321z, T322., T3220, T3221, T3222, T322y, T322z, T323., T3230, T3231, T3232, T323y, T323z, T324., T3240, T3241, T3242, T324y, T324z, T325., T3250, T3251, T3252, T325y, T325z, T326., T3260, T3261, T3262, T326y, T326z, T327., T3270, T3271, T3272, T327y, T327z, T328., T3280, T3281, T3282, T328y, T328z, T32z., T32z0, T32z1, T32z2, T32zy, T32zz, T33.., T330., T3300, T3301, T330y, T330z, T331., T3310, T3311, T331y, T331z, T332., T3320, T3321, T332y, T332z, T333., T3330, T3331, T333y, T333z, T334., T3340, T3341, T334y, T334z, T335., T3350, T3351, T335y, T335z, T336., T3360, T3361, T336y, T336z, T337., T3370, T3371, T337y, T337z, T338., T3380, T3381, T338y, T338z, T339., T3390, T3391, T339y, T339z, T33A., T33A0, T33A1, T33Ay, T33Az, T33B., T33B0, T33B1, T33By, T33Bz, T33C., T33C0, T33C1, T33Cy, T33Cz, T33x., T33x0, T33x1, T33xy, T33xz, T33y., T33y0, T33y1, T33yy, T33yz, T33z., T33z0, T33z1, T33zy, T33zz, T3z.., T4..., T41.., T413., T4130, T4131, T4132, T4133, T4134, T4135, T4136, T413y, T413z, T414., T4140, T4141, T4142, T4143, T4144, T4145, T4146, T414y, T414z, T415., T4150, T4151, T4152, T4153, T4154, T4155, T4156, T415y, T415z, T416., T4160, T4161, T4162, T4163, T4164, T4165, T4166, T416y, T416z, T41z., T41z0, T41z1, T41z2, T41z3, T41z4, T41z5, T41z6, T41zy, T41zz, T43.., T430., T4300, T4301, T4302, T4303, T4304, T4305, T4306, T430y, T430z, T431., T4310, T4311, T4312, T4313, T4314, T4315, T4316, T431y, T431z, T43z., T43z0, T43z1, T43z2, T43z3, T43z4, T43z5, T43z6, T43zy, T43zz, T44.., T440., T4400, T4401, T4402, T4403, T4404, T4405, T4406, T440y, T440z, T44z., T44z0, T44z1, T44z2, T44z3, T44z4, T44z5, T44z6, T44zy, T44zz, T45.., T450., T4500, T4501, T4502, T4503, T4504, T4505, T4506, T450y, T450z, T451., T4510, T4511, T4512, T4513, T4514, T4515, T4516, T451y, T451z, T452., T4520, T4521, T4522, T4523, T4524, T4525, T4526, T452y, T452z, T453., T4530, T4531, T4532, T4533, T4534, T4535, T4536, T453y, T453z, T454., T4540, T4541, T4542, T4543, T4544, T4545, T4546, T454y, T454z, T45z., T45z0, T45z1, T45z2, T45z3, T45z4, T45z5, T45z6, T45zy, T45zz, T46.., T460., T4600, T4601, T4602, T4603, T4604, T4605, T4606, T460y, T460z, T461., T4610, T4611, T4612, T4613, T4614, T4615, T4616, T461y, T461z, T46z., T46z0, T46z1, T46z2, T46z3, T46z4, T46z5, T46z6, T46zy, T46zz, T47.., T471., T4710, T4711, T4712, T4713, T4714, T4715, T4716, T471y, T471z, T476., T4760, T4761, T4762, T4763, T4764, T4765, T4766, T476y, T476z, T477., T4770, T4771, T4772, T4773, T4774, T4775, T4776, T477y, T477z, T47z., T47z0, T47z1, T47z2, T47z3, T47z4, T47z5, T47z6, T47zy, T47zz, T4z.., T5..., T50.., T500., T5000, T5001, T5002, T5003, T5004, T5005, T5006, T5007, T5008, T500z, T501., T5010, T5011, T5012, T5013, T5014, T5015, T5016, T5017, T5018, T501z, T502., T5020, T5021, T5022, T5023, T5024, T5025, T5026, T5027, T5028, T502z, T503., T5030, T5031, T5032, T5033, T5034, T5035, T5036, T5037, T5038, T503z, T504., T5040, T5041, T5042, T5043, T5044, T5045, T5046, T5047, T5048, T504z, T505., T5050, T5051, T5052, T5053, T5054, T5055, T5056, T5057, T5058, T505z, T506., T5060, T5061, T5062, T5063, T5064, T5065, T5066, T5067, T5068, T506z, T507., T5070, T5071, T5072, T5073, T5074, T5075, T5076, T5077, T5078, T507z, T508., T5080, T5081, T5082, T5083, T5084, T5085, T5086, T5087, T5088, T508z, T509., T5090, T5091, T5092, T5093, T5094, T5095, T5096, T5097, T5098, T509z, T50y., T50y0, T50y1, T50y2, T50y3, T50y4, T50y5, T50y6, T50y7, T50y8, T50yz, T50z., T50z0, T50z1, T50z2, T50z3, T50z4, T50z5, T50z6, T50z7, T50z8, T50zz, T51.., T510., T5100, T5101, T5102, T5103, T5104, T5105, T5106, T5107, T5108, T510z, T511., T5110, T5111, T5112, T5113, T5114, T5115, T5116, T5117, T5118, T511z, T512., T5120, T5121, T5122, T5123, T5124, T5125, T5126, T5127, T5128, T512z, T513., T5130, T5131, T5132, T5133, T5134, T5135, T5136, T5137, T5138, T513z, T514., T5140, T5141, T5142, T5143, T5144, T5145, T5146, T5147, T5148, T514z, T51z., T51z0, T51z1, T51z2, T51z3, T51z4, T51z5, T51z6, T51z7, T51z8, T51zz, T52.., T520., T5200, T5201, T5202, T520z, T521., T5210, T5211, T5212, T521z, T522., T5220, T5221, T5222, T522z, T523., T5230, T5231, T5232, T523z, T524., T5240, T5241, T5242, T524z, T52z., T52z0, T52z1, T52z2, T52zz, T53.., T530., T5300, T5301, T5302, T5303, T5304, T5305, T5306, T5307, T5308, T530z, T531., T5310, T5311, T5312, T5313, T5314, T5315, T5316, T5317, T5318, T531z, T532., T5320, T5321, T5322, T5323, T5324, T5325, T5326, T5327, T5328, T532z, T533., T5330, T5331, T5332, T5333, T5334, T5335, T5336, T5337, T5338, T533z, T534., T5340, T5341, T5342, T5343, T5344, T5345, T5346, T5347, T5348, T534z, T53z., T53z0, T53z1, T53z2, T53z3, T53z4, T53z5, T53z6, T53z7, T53z8, T53zz, T54.., T540., T5400, T5401, T5402, T5403, T5404, T5405, T5406, T5407, T5408, T540z, T541., T5410, T5411, T5412, T5413, T5414, T5415, T5416, T5417, T5418, T541z, T542., T5420, T5421, T5422, T5423, T5424, T5425, T5426, T5427, T5428, T542z, T543., T5430, T5431, T5432, T5433, T5434, T5435, T5436, T5437, T5438, T543z, T544., T5440, T5441, T5442, T5443, T5444, T5445, T5446, T5447, T5448, T544z, T546., T5460, T5461, T5462, T5463, T5464, T5465, T5466, T5467, T5468, T546z, T54z., T54z0, T54z1, T54z2, T54z3, T54z4, T54z5, T54z6, T54z7, T54z8, T54zz, T55.., T550., T5500, T5501, T550z, T55z., T55z0, T55z1, T55zz, T5z.., T6..., T60.., T600., T601., T602., T603., T604., T605., T606., T607., T608., T609., T60A., T60B., T60C., T60D., T60E., T60F., T60G., T60z., T61.., T610., T611., T612., T613., T614., T615., T616., T61z., T62.., T620., T621., T62z., T6z.., T7..., T70.., T700., T701., T702., T703., T704., T705., T706., T707., T708., T709., T70A., T70B., T70C., T70z., T71.., T710., T711., T71z., T72.., T720., T721., T722., T72z., T73.., T730., T731., T732., T733., T734., T735., T736., T737., T738., T739., T73A., T73B., T73C., T73D., T73z., T74.., T740., T741., T742., T743., T744., T745., T746., T747., T748., T749., T74A., T74B., T74C., T74D., T74E., T74F., T74G., T74H., T74J., T74K., T74L., T74M., T74N., T74P., T74Q., T74R., T74S., T74T., T74U., T74z., T75.., T750., T751., T75z., T76.., T760., T761., T762., T763., T764., T765., T766., T767., T768., T769., T76A., T76B., T76C., T76D., T76E., T76F., T76G., T76H., T76J., T76K., T76L., T76M., T76N., T76P., T76Q., T76R., T76S., T76T., T76U., T76V., T76z., T77.., T770., T771., T772., T773., T774., T775., T776., T777., T77z., T7y.., T7y0., T7y1., T7y2., T7y3., T7y4., T7y5., T7y6., T7y7., T7y8., T7y9., T7yA., T7yB., T7yC., T7yD., T7yE., T7yF., T7yG., T7yH., T7yJ., T7yK., T7yL., T7yM., T7yN., T7yP., T7yQ., T7yz., T7z.., TC..., TC0.., TC00., TC000, TC001, TC00z, TC01., TC010, TC011, TC01z, TC02., TC020, TC021, TC02z, TC0z., TC1.., TC10., TC11., TC1z., TC2.., TC20., TC21., TC22., TC23., TC24., TC25., TC26., TC27., TC28., TC29., TC2z., TC3.., TC30., TC300, TC301, TC302, TC303, TC304, TC305, TC30z, TC31., TC32., TC320, TC321, TC32z, TC3y., TC3y0, TC3y1, TC3y2, TC3y3, TC3y4, TC3y5, TC3y6, TC3yz, TC3z., TC4.., TC40., TC41., TC42., TC420, TC421, TC42z, TC4y., TC4y0, TC4y1, TC4y2, TC4y3, TC4yz, TC4z., TC5.., TC50., TC51., TC52., TC53., TC5z., TC6.., TC60., TC600, TC60y, TC60z, TC6y., TC6y0, TC6y1, TC6y2, TC6yz, TC6z., TC7.., TCy.., TCy0., TCyz., TCz.., TD..., TD0.., TD00., TD000, TD001, TD002, TD003, TD004, TD005, TD006, TD007, TD008, TD009, TD00A, TD00z, TD06., TD060, TD061, TD062, TD063, TD064, TD065, TD066, TD067, TD068, TD069, TD06A, TD06z, TD07., TD070, TD071, TD072, TD073, TD074, TD075, TD076, TD077, TD078, TD079, TD07A, TD07z, TD08., TD080, TD081, TD082, TD083, TD084, TD085, TD086, TD087, TD088, TD089, TD08A, TD08z, TD09., TD090, TD091, TD092, TD093, TD094, TD095, TD096, TD097, TD098, TD099, TD09A, TD09z, TD0y., TD0y0, TD0y1, TD0y2, TD0y3, TD0y4, TD0y5, TD0y6, TD0y7, TD0y8, TD0y9, TD0yA, TD0yz, TD0z., TD1.., TD10., TD100, TD101, TD102, TD103, TD104, TD105, TD106, TD107, TD108, TD109, TD10A, TD10B, TD10z, TD16., TD160, TD161, TD162, TD163, TD164, TD165, TD166, TD167, TD168, TD169, TD16A, TD16B, TD16z, TD17., TD170, TD171, TD172, TD173, TD174, TD175, TD176, TD177, TD178, TD179, TD17A, TD17B, TD17z, TD18., TD180, TD181, TD182, TD183, TD184, TD185, TD186, TD187, TD188, TD189, TD18A, TD18B, TD18z, TD19., TD190, TD191, TD192, TD193, TD194, TD195, TD196, TD197, TD198, TD199, TD19A, TD19B, TD19z, TD1y., TD1y0, TD1y1, TD1y2, TD1y3, TD1y4, TD1y5, TD1y6, TD1y7, TD1y8, TD1y9, TD1yA, TD1yB, TD1yz, TD1z., TD2.., TD20., TD21., TD22., TD23., TD24., TD25., TD26., TD27., TD2z., TD5.., TD50., TD51., TD52., TD53., TD54., TD55., TD56., TD57., TD570, TD571, TD572, TD573, TD5z., TD6.., TD60., TD61., TD62., TD63., TD64., TD65., TD66., TD67., TD6z., TD7.., TD70., TD71., TD72., TD7z., TDy.., TDy0., TDyy., TDyy0, TDyy1, TDyy2, TDyy3, TDyy4, TDyy5, TDyy6, TDyy7, TDyy8, TDyy9, TDyyA, TDyyz, TDyz., TDz.., TDz1., TE8.., TE80., TE81., TE82., TE83., TE84., TE85., TE86., TE87., TE88., TE89., TE8A., TE8z., TE9.., TE90., TE91., TE92., TE93., TE94., TE95., TE96., TE97., TE9z., TEz.., TF..., TFz.., TG..., TG0.., TG00., TG01., TG02., TG03., TG04., TG05., TG06., TG0z., TG1.., TG10., TG100, TG101, TG102, TG103, TG104, TG105, TG106, TG107, TG10y, TG10z, TG11., TG110, TG111, TG112, TG11z, TG12., TG13., TG130, TG131, TG132, TG133, TG134, TG13z, TG14., TG140, TG141, TG142, TG143, TG144, TG14z, TG15., TG150, TG151, TG152, TG153, TG154, TG15z, TG16., TG160, TG161, TG162, TG163, TG164, TG16z, TG17., TG170, TG171, TG172, TG173, TG174, TG17z, TG1z., TG1z0, TG1zz, TG2.., TG20., TG21., TG22., TG23., TG24., TG25., TG26., TG2z., TG3.., TG30., TG301, TG302, TG306, TG307, TG308, TG309, TG30B, TG30C, TG30D, TG30E, TG30F, TG30G, TG30H, TG30z, TG31., TG310, TG311, TG312, TG313, TG314, TG315, TG316, TG317, TG31z, TG32., TG320, TG321, TG322, TG323, TG324, TG32z, TG33., TG330, TG331, TG332, TG333, TG334, TG335, TG336, TG337, TG338, TG33z, TG34., TG340, TG341, TG342, TG343, TG344, TG345, TG346, TG347, TG348, TG34z, TG35., TG350, TG351, TG352, TG353, TG354, TG355, TG356, TG357, TG35z, TG36., TG360, TG361, TG362, TG363, TG36z, TG37., TG370, TG371, TG372, TG373, TG374, TG375, TG376, TG37z, TG38., TG380, TG381, TG382, TG3y., TG3y0, TG3y1, TG3y2, TG3y3, TG3y4, TG3y5, TG3y6, TG3y7, TG3yz, TG3z., TG4.., TG40., TG41., TG410, TG411, TG412, TG413, TG414, TG415, TG41z, TG42., TG420, TG421, TG422, TG423, TG424, TG425, TG42z, TG43., TG430, TG431, TG432, TG43z, TG44., TG440, TG441, TG442, TG443, TG444, TG445, TG446, TG447, TG448, TG449, TG44A, TG44B, TG44C, TG44D, TG44E, TG44z, TG4y., TG4y0, TG4y1, TG4y2, TG4y3, TG4y4, TG4y5, TG4y6, TG4y7, TG4y8, TG4yz, TG4z., TG5.., TG50., TG51., TG510, TG511, TG51z, TG5y., TG5y0, TG5y1, TG5y2, TG5yz, TG5z., TG6.., TG60., TG600, TG601, TG60z, TG61., TG62., TG63., TG630, TG631, TG63z, TG6y., TG6y0, TG6yz, TG6z., TG6z0, TG6z1, TG6zz, TG7.., TG70., TG71., TG710, TG711, TG712, TG713, TG71z, TG72., TG720, TG721, TG722, TG723, TG724, TG725, TG726, TG72y, TG72z, TG7y., TG7y0, TG7y1, TG7y2, TG7y3, TG7y4, TG7y5, TG7y6, TG7y7, TG7yz, TG7z., TGyz., TGyz0, TGyz1, TGyz3, TGyz4, TGyz5, TGyzz, TGz.., TK..., TK5.., TK50., TK51., TK52., TK53., TK54., TK55., TK5z., TK6.., TK60., TK601, TK61., TK6z., TK7.., TK70., TK71., TK72., TK7z., TKx.., TKx0., TKx00, TKx01, TKx0z, TKx5., TKx6., TKxy., TKxz., TKz.., TL..., TL0.., TL00., TL01., TL0z., TL5.., TL50., TL500, TL501, TL50z, TL51., TL52., TL53., TL54., TL55., TL56., TL57., TL5x., TL5y., TL5y0, TL5y1, TL5y2, TL5yy, TL5yz, TL5z., TL6.., TL60., TL61., TL62., TL63., TL64., TL6z., TL7.., TL70., TL7y., TL7z., TL9.., TLx.., TLx0., TLx00, TLx1., TLx2., TLx20, TLx21, TLx2z, TLxy., TLxy0, TLxyz, TLxz., TLxz0, TLxz1, TLxz2, TLxz3, TLxz4, TLxzz, TLz.., TM..., TM0.., TM00., TM01., TM02., TM03., TM0z., TM1.., TM10., TM11., TM12., TM13., TM1z., TM3.., TM30., TM31., TM3z., TM4.., TM40., TM4x., TM4y., TM4z., TM5.., TM50., TM51., TM5z., TM6.., TM8.., TM85., TM8z., TMz.., TN..., TN5.., TN50., TN51., TN52., TN53., TN54., TN55., TN5z., TN6.., TN60., TN61., TN6z., TN7.., TN70., TN71., TN72., TN7z., TN8.., TN80., TN800, TN801, TN80z, TN85., TN86., TN8y., TN8z., TNz.., TP..., TP0.., TP00., TP0z., TP1.., TP10., TP11., TP12., TP120, TP121, TP122, TP123, TP124, TP12z, TP13., TP1z., TP1z0, TP1z1, TP1z2, TP1z3, TP1z4, TP1z5, TP1z6, TP1z7, TP1zz, TP2.., TP20., TP21., TP22., TP23., TP24., TP2z., TP3.., TP30., TP31., TP32., TP33., TP34., TP35., TP36., TP3z., TP4.., TP40., TP41., TP42., TP4z., TP5.., TP50., TP5z., TP6.., TP60., TP61., TP62., TP63., TP6z., TP8.., TPz.., Tz..., U0..., U00.., U000., U0000, U0001, U000z, U001., U0010, U0011, U001z, U002., U0020, U0021, U002z, U003., U0030, U0031, U003z, U004., U0040, U0041, U004z, U005., U0050, U0051, U005z, U00z., U00z0, U00z1, U00z2, U00z3, U00zz, U01.., U010., U0100, U0101, U0102, U0103, U0104, U0105, U010z, U011., U0110, U0111, U0112, U0113, U0114, U0115, U011z, U012., U0120, U0121, U0122, U0123, U0124, U0125, U012z, U013., U0130, U0131, U0132, U0133, U0134, U0135, U013z, U014., U0140, U0141, U0142, U0143, U0144, U0145, U014z, U015., U0150, U0151, U0152, U0153, U0154, U0155, U015z, U016., U0160, U0161, U0162, U0163, U0164, U0165, U016z, U017., U0170, U0171, U0172, U0173, U0174, U0175, U017z, U018., U0180, U0181, U0182, U0183, U0184, U0185, U018z, U01z., U01z0, U01z1, U01z2, U01z3, U01z4, U01z5, U01z6, U01z8, U01zz, U02.., U020., U0200, U0201, U0202, U0203, U0204, U0205, U020z, U021., U0210, U0211, U0212, U0213, U0214, U0215, U021z, U022., U0220, U0221, U0222, U0223, U0224, U0225, U022z, U023., U0230, U0231, U0232, U0233, U0234, U0235, U023z, U024., U0240, U0241, U0242, U0243, U0244, U0245, U024z, U025., U0250, U0251, U0252, U0253, U0254, U0255, U025z, U026., U0260, U0261, U0262, U0263, U0264, U0265, U026z, U027., U0270, U0271, U0272, U0273, U0274, U0275, U027z, U028., U0280, U0281, U0282, U0283, U0284, U0285, U028z, U02z., U02z0, U02z1, U02z2, U02z3, U02z4, U02z5, U02z6, U02z8, U02zz, U03.., U030., U0300, U0301, U0302, U0303, U0304, U0305, U0306, U0307, U030z, U031., U0310, U0311, U0312, U0313, U0314, U0315, U0316, U0317, U031z, U032., U0320, U0321, U0322, U0323, U0324, U0325, U0326, U0327, U032z, U033., U0330, U0331, U0332, U0333, U0334, U0335, U0336, U0337, U033z, U034., U0340, U0341, U0342, U0343, U0344, U0345, U0346, U0347, U034z, U035., U0350, U0351, U0352, U0353, U0354, U0355, U0356, U0357, U035z, U036., U0360, U0361, U0362, U0363, U0364, U0365, U0366, U0367, U036z, U037., U0370, U0371, U0372, U0373, U0374, U0375, U0376, U0377, U037z, U038., U0380, U0381, U0382, U0383, U0384, U0385, U0386, U0387, U038z, U03z., U03z0, U03z1, U03z2, U03z3, U03z4, U03z5, U03z6, U03z8, U03zz, U04.., U040., U0400, U0401, U0402, U0403, U0404, U0405, U0406, U0407, U040z, U041., U0410, U0411, U0412, U0413, U0414, U0415, U0416, U0417, U041z, U042., U0420, U0421, U0422, U0423, U0424, U0425, U0426, U0427, U042z, U043., U0430, U0431, U0432, U0433, U0434, U0435, U0436, U0437, U043z, U044., U0440, U0441, U0442, U0443, U0444, U0445, U0446, U0447, U044z, U045., U0450, U0451, U0452, U0453, U0454, U0455, U0456, U0457, U045z, U046., U0460, U0461, U0462, U0463, U0464, U0465, U0466, U0467, U046z, U047., U0470, U0471, U0472, U0473, U0474, U0475, U0476, U0477, U047z, U048., U0480, U0481, U0482, U0483, U0484, U0485, U0486, U0487, U048z, U04z., U04z0, U04z1, U04z2, U04z3, U04z4, U04z5, U04z6, U04z8, U04zz, U05.., U050., U0500, U0501, U0502, U0503, U0504, U0505, U0506, U0507, U050z, U051., U0510, U0511, U0512, U0513, U0514, U0515, U0516, U0517, U051z, U052., U0520, U0521, U0522, U0523, U0524, U0525, U0526, U0527, U052z, U053., U0530, U0531, U0532, U0533, U0534, U0535, U0536, U0537, U053z, U054., U0540, U0541, U0542, U0543, U0544, U0545, U0546, U0547, U054z, U055., U0550, U0551, U0552, U0553, U0554, U0555, U0556, U0557, U055z, U056., U0560, U0561, U0562, U0563, U0564, U0565, U0566, U0567, U056z, U057., U0570, U0571, U0572, U0573, U0574, U0575, U0576, U0577, U057z, U058., U0580, U0581, U0582, U0583, U0584, U0585, U0586, U0587, U058z, U05z., U05z0, U05z1, U05z2, U05z3, U05z4, U05z5, U05z6, U05z8, U05zz, U06.., U060., U0600, U0601, U0602, U0603, U0604, U0605, U0606, U0607, U060z, U061., U0610, U0611, U0612, U0613, U0614, U0615, U0616, U0617, U061z, U062., U0620, U0621, U0622, U0623, U0624, U0625, U0626, U0627, U062z, U063., U0630, U0631, U0632, U0633, U0634, U0635, U0636, U0637, U063z, U064., U0640, U0641, U0642, U0643, U0644, U0645, U0646, U0647, U064z, U065., U0650, U0651, U0652, U0653, U0654, U0655, U0656, U0657, U065z, U066., U0660, U0661, U0662, U0663, U0664, U0665, U0666, U0667, U066z, U067., U0670, U0671, U0672, U0673, U0674, U0675, U0676, U0677, U067z, U068., U0680, U0681, U0682, U0683, U0684, U0685, U0686, U0687, U068z, U06z., U06z0, U06z1, U06z2, U06z3, U06z4, U06z5, U06z6, U06z8, U06zz, U07.., U070., U0700, U0701, U0702, U0703, U0704, U0705, U0706, U0707, U070z, U071., U0710, U0711, U0712, U0713, U0714, U0715, U0716, U0717, U071z, U072., U0720, U0721, U0722, U0723, U0724, U0725, U0726, U0727, U072z, U073., U0730, U0731, U0732, U0733, U0734, U0735, U0736, U0737, U073z, U074., U0740, U0741, U0742, U0743, U0744, U0745, U0746, U0747, U074z, U075., U0750, U0751, U0752, U0753, U0754, U0755, U0756, U0757, U075z, U076., U0760, U0761, U0762, U0763, U0764, U0765, U0766, U0767, U076z, U077., U0770, U0771, U0772, U0773, U0774, U0775, U0776, U0777, U077z, U078., U0780, U0781, U0782, U0783, U0784, U0785, U0786, U0787, U078z, U07z., U07z0, U07z1, U07z2, U07z3, U07z4, U07z5, U07z6, U07z8, U07zz, U08.., U080., U0800, U0801, U0802, U0803, U0804, U0805, U0806, U0807, U0808, U080z, U081., U0810, U0811, U0812, U0813, U0814, U0815, U0816, U0817, U0818, U081z, U082., U0820, U0821, U0822, U0823, U0824, U0825, U0826, U0827, U0828, U082z, U083., U0830, U0831, U0832, U0833, U0834, U0835, U0836, U0837, U083z, U084., U0840, U0841, U0842, U0843, U0844, U0845, U0846, U0847, U084z, U085., U0850, U0851, U0852, U0853, U0854, U0855, U0856, U0857, U085z, U086., U0860, U0861, U0862, U0863, U0864, U0865, U0866, U0867, U086z, U087., U0870, U0871, U0872, U0873, U0874, U0875, U0876, U0877, U0878, U087z, U088., U0880, U0881, U0882, U0883, U0884, U0885, U0886, U0887, U0888, U088z, U08z., U08z0, U08z1, U08z2, U08z3, U08zz, U09.., U091., U0910, U0911, U0912, U0913, U0914, U0915, U0916, U0917, U0918, U091z, U093., U0930, U0931, U0932, U0933, U0934, U0935, U0936, U0937, U0938, U093z, U09z., U09z0, U09z1, U09z2, U09z3, U09z4, U09z5, U09z6, U09z7, U09z8, U09zz, U0A.., U0A0., U0A00, U0A01, U0A02, U0A03, U0A04, U0A0y, U0A0z, U0A1., U0A10, U0A11, U0A12, U0A1y, U0A1z, U0Ay., U0Ay0, U0Ay1, U0Ay2, U0Ay3, U0Ayy, U0z.., U0zy., U0zz., U1..., U10.., U100., U1000, U1001, U1002, U1003, U1004, U1005, U1006, U1007, U100y, U100z, U101., U1010, U1011, U1012, U1013, U1014, U1015, U1016, U1017, U101y, U101z, U102., U1020, U1021, U1022, U1023, U1024, U1025, U1026, U1027, U102y, U102z, U103., U1030, U1031, U1032, U1033, U1034, U1035, U1036, U1037, U103y, U103z, U104., U1040, U1041, U1042, U1043, U1044, U1045, U1046, U1047, U104y, U104z, U105., U1050, U1051, U1052, U1053, U1054, U1055, U1056, U1057, U105y, U105z, U106., U1060, U1061, U1062, U1063, U1064, U1065, U1066, U1067, U106y, U106z, U107., U1070, U1071, U1072, U1073, U1074, U1075, U1076, U1077, U107y, U107z, U108., U1080, U1081, U1082, U1083, U1084, U1085, U1086, U1087, U108y, U108z, U109., U1090, U1091, U1092, U1093, U1094, U1095, U1096, U1097, U109y, U109z, U10A., U10A0, U10A1, U10A2, U10A3, U10A4, U10A5, U10A6, U10A7, U10Ay, U10Az, U10B., U10B0, U10B1, U10B2, U10B3, U10B4, U10B5, U10B6, U10B7, U10By, U10Bz, U10C., U10C0, U10C1, U10C2, U10C3, U10C4, U10C5, U10C6, U10C7, U10Cy, U10Cz, U10D., U10D0, U10D1, U10D2, U10D3, U10D4, U10D5, U10D6, U10D7, U10Dy, U10Dz, U10E., U10E0, U10E1, U10E2, U10E3, U10E4, U10E5, U10E6, U10E7, U10Ey, U10Ez, U10F., U10F0, U10F1, U10F2, U10F3, U10F4, U10F5, U10F6, U10F7, U10Fy, U10Fz, U10G., U10G0, U10G1, U10G2, U10G3, U10G4, U10G5, U10G6, U10G7, U10Gy, U10Gz, U10H., U10H0, U10H1, U10H2, U10H3, U10H4, U10H5, U10H6, U10H7, U10Hy, U10Hz, U10J., U10J0, U10J1, U10J2, U10J3, U10J4, U10J5, U10J6, U10J7, U10Jy, U10Jz, U10z., U10z0, U10z1, U10z2, U10z3, U10z4, U10z5, U10z6, U10z7, U10zy, U10zz, U11.., U110., U1100, U1101, U1102, U1103, U1104, U1105, U1106, U1107, U110y, U110z, U111., U1110, U1111, U1112, U1113, U1114, U1115, U1116, U1117, U111y, U111z, U112., U1120, U1121, U1122, U1123, U1124, U1125, U1126, U1127, U112y, U112z, U113., U1130, U1131, U1132, U1133, U1134, U1135, U1136, U1137, U113y, U113z, U114., U1140, U1141, U1142, U1143, U1144, U1145, U1146, U1147, U114y, U114z, U115., U1150, U1151, U1152, U1153, U1154, U1155, U1156, U1157, U115y, U115z, U116., U1160, U1161, U1162, U1163, U1164, U1165, U1166, U1167, U116y, U116z, U117., U1170, U1171, U1172, U1173, U1174, U1175, U1176, U1177, U1178, U117z, U118., U1180, U1181, U1182, U1183, U1184, U1185, U1186, U1187, U118y, U118z, U119., U1190, U1191, U1192, U1193, U1194, U1195, U1196, U1197, U119y, U119z, U11A., U11A0, U11A1, U11A2, U11A3, U11A4, U11A5, U11A6, U11A7, U11Ay, U11Az, U11B., U11B0, U11B1, U11B2, U11B3, U11B4, U11B5, U11B6, U11B7, U11By, U11Bz, U11C., U11C0, U11C1, U11C2, U11C3, U11C4, U11C5, U11C6, U11C7, U11Cy, U11Cz, U11D., U11D0, U11D1, U11D2, U11D3, U11D4, U11D5, U11D6, U11D7, U11Dy, U11Dz, U11E., U11E0, U11E1, U11E2, U11E3, U11E4, U11E5, U11E6, U11E7, U11E8, U11E9, U11F., U11F0, U11F1, U11F2, U11F3, U11F4, U11F5, U11F6, U11F7, U11Fy, U11Fz, U11G., U11G0, U11G1, U11G2, U11G3, U11G4, U11G5, U11G6, U11G7, U11Gy, U11Gz, U11H., U11H0, U11H1, U11H2, U11H3, U11H4, U11H5, U11H6, U11H7, U11Hy, U11Hz, U11J., U11J0, U11J1, U11J2, U11J3, U11J4, U11J5, U11J6, U11J7, U11Jy, U11Jz, U11K., U11K0, U11K1, U11K2, U11K3, U11K4, U11K5, U11K6, U11K7, U11Ky, U11Kz, U11L., U11L0, U11L1, U11L2, U11L3, U11L4, U11L5, U11L6, U11L7, U11Ly, U11Lz, U11M., U11M0, U11M1, U11M2, U11M3, U11M4, U11M5, U11M6, U11M7, U11My, U11Mz, U11y., U11y0, U11y1, U11y2, U11y3, U11y4, U11y5, U11y6, U11y7, U11yy, U11yz, U12.., U120., U1200, U1201, U1202, U1203, U1204, U1205, U1206, U1207, U120y, U120z, U121., U1210, U1211, U1212, U1213, U1214, U1215, U1216, U1217, U121y, U121z, U122., U1220, U1221, U1222, U1223, U1224, U1225, U1226, U1227, U122y, U122z, U123., U1230, U1231, U1232, U1233, U1234, U1235, U1236, U1237, U123y, U123z, U124., U1240, U1241, U1242, U1243, U1244, U1245, U1246, U1247, U124y, U124z, U125., U1250, U1251, U1252, U1253, U1254, U1255, U1256, U1257, U125y, U125z, U126., U1260, U1261, U1262, U1263, U1264, U1265, U1266, U1267, U126y, U126z, U128., U1280, U1281, U1282, U1283, U1284, U1285, U1286, U1287, U128y, U128z, U129., U1290, U1291, U1292, U1293, U1294, U1295, U1296, U1297, U129y, U129z, U12y., U12y0, U12y1, U12y2, U12y3, U12y4, U12y5, U12y6, U12y7, U12yy, U12yz, U15.., U16.., U160., U1600, U1601, U1602, U1603, U1604, U1605, U1606, U1607, U160y, U160z, U161., U1610, U1611, U1612, U1613, U1614, U1615, U1616, U1617, U161y, U161z, U162., U1620, U1621, U1622, U1623, U1624, U1625, U1626, U1627, U162y, U162z, U163., U1630, U1631, U1632, U1633, U1635, U1636, U1637, U1638, U163y, U163z, U164., U1640, U1641, U1642, U1643, U1644, U1645, U1646, U1647, U164y, U164z, U16y., U16y0, U16y1, U16y2, U16y3, U16y4, U16y5, U16y6, U16y7, U16yy, U16yz, U16z., U16z0, U16z1, U16z2, U16z3, U16z4, U16z5, U16z6, U16z7, U16zy, U16zz, U18.., U180., U1800, U1801, U1802, U1803, U1804, U1805, U1806, U1807, U180y, U180z, U181., U1810, U1811, U1812, U1813, U1814, U1815, U1816, U1817, U181y, U181z, U182., U1820, U1821, U1822, U1823, U1824, U1825, U1826, U1827, U182y, U182z, U183., U1830, U1831, U1832, U1833, U1834, U1835, U1836, U1837, U183y, U183z, U184., U1840, U1841, U1842, U1843, U1844, U1845, U1846, U1847, U184y, U184z, U185., U1850, U1851, U1852, U1853, U1854, U1855, U1856, U1857, U185y, U185z, U186., U1860, U1861, U1862, U1863, U1864, U1865, U1866, U1867, U186y, U186z, U187., U1870, U1871, U1872, U1873, U1874, U1875, U1876, U1877, U187y, U187z, U18y., U18y0, U18y1, U18y2, U18y3, U18y4, U18y5, U18y6, U18y7, U18yy, U18yz, U18z., U18z0, U18z1, U18z2, U18z3, U18z4, U18z5, U18z6, U18z7, U18zy, U18zz, U194., U1940, U1941, U1942, U1943, U1944, U1945, U1946, U1947, U194y, U194z, U195., U1950, U1951, U1952, U1953, U1954, U1955, U1956, U1957, U195y, U195z, U196., U1960, U1961, U1962, U1963, U1964, U1965, U1966, U1967, U196y, U196z, U197., U1970, U1971, U1972, U1973, U1974, U1975, U1976, U1977, U197y, U197z, U198., U1980, U1981, U1982, U1983, U1984, U1985, U1986, U1987, U198y, U198z, U1C.., U1Cy., U1Cy0, U1Cy1, U1Cy2, U1Cy3, U1Cy4, U1Cy5, U1Cy6, U1Cy7, U1Cyy, U1Cyz, U1Cz., U1Cz0, U1Cz1, U1Cz2, U1Cz3, U1Cz4, U1Cz5, U1Cz6, U1Cz7, U1Czy, U1Czz, U2..., U23.., U230., U231., U232., U233., U234., U235., U236., U237., U23y., U23z., U24.., U240., U241., U242., U243., U244., U245., U246., U247., U24y., U24z., U25.., U250., U251., U252., U253., U254., U255., U256., U257., U25y., U25z., U26.., U260., U261., U262., U263., U264., U265., U266., U267., U26y., U26z., U27.., U270., U271., U272., U273., U274., U275., U276., U277., U27y., U27z., U29.., U290., U291., U292., U293., U294., U295., U296., U297., U29y., U29z., U2A.., U2A0., U2A1., U2A2., U2A3., U2A4., U2A5., U2A6., U2A7., U2Ay., U2Az., U2B.., U2B0., U2B1., U2B2., U2B3., U2B4., U2B5., U2B6., U2B7., U2By., U2Bz., U2C.., U2C0., U2C1., U2C2., U2C3., U2C4., U2C5., U2C6., U2C7., U2Cy., U2Cz., U2D.., U2D0., U2D1., U2D2., U2D3., U2D4., U2D5., U2D6., U2D7., U2Dy., U2Dz., U2E.., U2y.., U2y0., U2y1., U2y2., U2y3., U2y4., U2y5., U2y6., U2y7., U2yy., U2yz., U2z.., U2z0., U2z1., U2z2., U2z3., U2z4., U2z5., U2z6., U2z7., U2zy., U2zz., U3..., U30.., U300., U301., U302., U303., U304., U305., U306., U307., U30y., U30z., U32.., U320., U321., U322., U323., U324., U325., U326., U327., U32y., U32z., U34.., U340., U341., U342., U343., U344., U345., U346., U347., U34y., U34z., U35.., U350., U351., U352., U353., U354., U355., U356., U357., U35y., U35z., U38.., U380., U381., U382., U383., U384., U385., U386., U387., U38y., U38z., U39.., U390., U391., U392., U393., U394., U395., U396., U397., U39y., U39z., U3A.., U3A0., U3A1., U3A2., U3A3., U3A4., U3A5., U3A6., U3A7., U3Ay., U3Az., U3B.., U3B0., U3B1., U3B2., U3B3., U3B4., U3B5., U3B6., U3B7., U3By., U3Bz., U3C.., U3C0., U3C1., U3C2., U3C3., U3C4., U3C5., U3C6., U3C7., U3Cy., U3Cz., U3E.., U3E0., U3E1., U3E2., U3E3., U3E4., U3E5., U3E6., U3E7., U3Ey., U3Ez., U3F.., U3F0., U3F1., U3F2., U3F3., U3F4., U3F5., U3F6., U3F7., U3Fy., U3Fz., U3G.., U3G0., U3G1., U3G2., U3G3., U3G4., U3G5., U3G6., U3G7., U3Gy., U3Gz., U3H.., U3H0., U3H1., U3H2., U3H3., U3H4., U3H5., U3H6., U3H7., U3Hy., U3Hz., U3J.., U3J0., U3J1., U3J2., U3J3., U3J4., U3J5., U3J6., U3J7., U3Jy., U3Jz., U3K.., U3K0., U3K1., U3K2., U3K3., U3K4., U3K5., U3K6., U3K7., U3Ky., U3Kz., U3y.., U3y0., U3y1., U3y2., U3y3., U3y4., U3y5., U3y6., U3y7., U3yy., U3yz., U3z.., U3z0., U3z1., U3z2., U3z3., U3z4., U3z5., U3z6., U3z7., U3zy., U3zz., U4..., U43.., U430., U431., U432., U433., U434., U435., U436., U437., U43y., U43z., U44.., U440., U441., U442., U443., U444., U445., U446., U447., U44y., U44z., U45.., U450., U451., U452., U453., U454., U455., U456., U457., U45y., U45z., U46.., U460., U461., U462., U463., U464., U465., U466., U467., U46y., U46z., U47.., U470., U471., U472., U473., U474., U475., U476., U477., U47y., U47z., U49.., U490., U491., U492., U493., U494., U495., U496., U497., U49y., U49z., U4A.., U4A0., U4A1., U4A2., U4A3., U4A4., U4A5., U4A6., U4A7., U4Ay., U4Az., U4B.., U4B0., U4B1., U4B2., U4B3., U4B4., U4B5., U4B6., U4B7., U4By., U4Bz., U4C.., U4C0., U4C1., U4C2., U4C3., U4C4., U4C5., U4C6., U4C7., U4Cy., U4Cz., U4D.., U4D0., U4D1., U4D2., U4D3., U4D4., U4D5., U4D6., U4D7., U4Dy., U4Dz., U4y.., U4y0., U4y1., U4y2., U4y3., U4y4., U4y5., U4y6., U4y7., U4yy., U4yz., U4z.., U4z0., U4z1., U4z2., U4z3., U4z4., U4z5., U4z6., U4z7., U4zy., U4zz., U5..., U50.., U500., U501., U503., U504., U505., U50y., U50z., U51.., U510., U511., U512., U513., U514., U515., U516., U517., U518., U51z. |
| **Injury Part of Body** | |
| 1 Head/Skull | S0..., S00.., S000., S0000, S0001, S0002, S0003, S0004, S0005, S0006, S000z, S001., S0010, S0011, S0012, S0013, S0014, S0015, S0016, S001z, S002., S0020, S0021, S0022, S0023, S0024, S0025, S0026, S002z, S003., S0030, S0031, S0032, S0033, S0034, S0035, S0036, S003z, S00z., S01.., S010., S0100, S0101, S0102, S0103, S0104, S0105, S0106, S010z, S011., S0110, S0111, S0112, S0113, S0114, S0115, S0116, S011z, S012., S0120, S0121, S0122, S0123, S0124, S0125, S0126, S012z, S013., S0130, S0131, S0132, S0133, S0134, S0135, S0136, S013z, S01z., S02.., S020., S021., S022., S0220, S0221, S0222, S0223, S0224, S0225, S0226, S0227, S0228, S022x, S022z, S023., S0230, S0231, S0232, S0233, S0234, S0235, S0236, S0237, S0238, S023x, S023z, S024., S0240, S0241, S024z, S025., S0250, S0251, S025z, S026., S027., S028., S0280, S0281, S0282, S0283, S02A., S02B., S02C., S02x., S02x0, S02x1, S02x2, S02xz, S02y., S02y0, S02y1, S02y2, S02yz, S02z., S03.., S030., S0300, S0301, S0302, S0303, S0304, S0305, S0306, S030z, S031., S0310, S0311, S0312, S0313, S0314, S0315, S0316, S031z, S032., S0320, S0321, S0322, S0323, S0324, S0325, S0326, S032z, S033., S0330, S0331, S0332, S0333, S0334, S0335, S0336, S033z, S03z., S04.., S040., S0400, S0401, S0402, S0403, S0404, S0405, S0406, S040z, S041., S0410, S0411, S0412, S0413, S0414, S0415, S0416, S041z, S042., S0420, S0421, S0422, S0423, S0424, S0425, S0426, S042z, S043., S0430, S0431, S0432, S0433, S0434, S0435, S0436, S043z, S044., S04z., S0z.., S6..., S60.., S600., S601., S602., S603., S604., S605., S60z., S61.., S610., S6100, S6101, S6102, S6103, S6104, S6105, S6106, S610z, S611., S6110, S6111, S6112, S6113, S6114, S6115, S6116, S611z, S612., S6120, S6121, S6122, S6123, S6124, S6125, S6126, S612z, S613., S6130, S6131, S6132, S6133, S6134, S6135, S6136, S613z, S614., S6140, S6141, S6142, S6143, S6144, S6145, S6146, S614z, S615., S6150, S6151, S6152, S6153, S6154, S6155, S6156, S615z, S616., S6160, S6161, S6162, S6163, S6164, S6165, S6166, S616z, S617., S6170, S6171, S6172, S6173, S6174, S6175, S6176, S617z, S61x., S61x0, S61x1, S61x2, S61x3, S61x4, S61x5, S61x6, S61xz, S61y., S61y0, S61y1, S61y2, S61y3, S61y4, S61y5, S61y6, S61yz, S61z., S62.., S620., S6200, S6201, S6202, S6203, S6204, S6205, S6206, S620z, S621., S6210, S6211, S6212, S6213, S6214, S6215, S6216, S621z, S622., S6220, S6221, S6222, S6223, S6224, S6225, S6226, S622z, S623., S6230, S6231, S6232, S6233, S6234, S6235, S6236, S623z, S624., S6240, S6241, S6242, S6243, S6244, S6245, S6246, S624z, S625., S6250, S6251, S6252, S6253, S6254, S6255, S6256, S625z, S626., S627., S628., S629., S6290, S6291, S62A., S62A0, S62A1, S62z., S63.., S630., S6300, S6301, S6302, S6303, S6304, S6305, S6306, S630z, S631., S6310, S6311, S6312, S6313, S6314, S6315, S6316, S631z, S63z., S64.., S640., S6400, S6401, S6402, S6403, S6404, S6405, S6406, S640z, S641., S6410, S6411, S6412, S6413, S6414, S6415, S6416, S641z, S642., S6420, S6421, S643., S644., S645., S6450, S6451, S646., S6460, S64z., S6z.., S83.., S830., S8301, S831., S83x., S83y., S83z., S8W.., S8X.., SD0.., SD00., SD002, SD00z, SD01., SD012, SD01z, SD02., SD022, SD02z, SD03., SD032, SD03z, SD04., SD042, SD04z, SD05., SD052, SD05z, SD06., SD062, SD06z, SD07., SD072, SD07z, SD08., SD0y., SD0y2, SD0z2, SD0zz, SE09., SF01., SF03., SF0X., SH106, SH116, SH126, SH12G, SH136, SH146, SH156, SJ0.., SJ00., SJ01., SJ02., SJ03., SJ0z., SJ1.., SJ10., SJ11., SJ12., SJ13., SK109, SK18., SK1x0, Syu0., Syu01, Syu02, Syu03, Syu04, Syu05, Syu06, Syu07, Syu0D, Syu0E, Syu0F, Syu0G, Syu0H, Syu0J, Syu0K, Syu0L |
| 2 Face (excl. eye) | S40.., S400., S401., S402., S403., S40z., S49G., S5E0., S5P0., S5P00, S5P0z, S5y0., S5y1., S5y10, S5y11, S5y1z, S82.., S820., S8200, S8201, S8202, S820w, S820z, S821., S8210, S8211, S8212, S821z, S822., S82v., S82v0, S82v1, S82v2, S82v3, S82vz, S82w., S82w0, S82w1, S82w2, S82w3, S82wz, S82x., S82y., S82z., S832., S8320, S8321, S8322, S8323, S832x, S832z, S833., S8330, S8331, S8332, S8333, S833x, S833z, S834., S8340, S8341, S8342, S8343, S8344, S8345, S8346, S834x, S834z, S835., S8350, S8351, S8352, S8353, S8354, S8355, S835x, S835z, S836., S8360, S8361, S8362, S8363, S8364, S8365, S8366, S8367, S836x, S836z, S837., S8370, S8371, S8372, S8373, S8374, S8375, S8376, S837x, S837z, SD000, SD010, SD020, SD030, SD040, SD050, SD060, SD070, SD0y0, SD0z0, SE00., SE01., SE02., SE03., SE04., SE05., SE06., SF00., SF000, SF001, SF00z, SG1.., SG10., SG11., SG1z., SG2.., SG20., SG21., SG22., SG2z., SH101, SH103, SH104, SH105, SH107, SH108, SH111, SH113, SH114, SH115, SH117, SH118, SH121, SH123, SH124, SH125, SH127, SH128, SH12B, SH12D, SH12E, SH12F, SH12H, SH12J, SH131, SH133, SH134, SH135, SH137, SH138, SH141, SH143, SH144, SH145, SH147, SH148, SH151, SH153, SH154, SH155, SH157, SH158, SK100, SK101, SK102, SK103, SK104, SK105, SK10x |
| 3 Eye | S80.., S800., S801., S802., S803., S804., S80y., S80z., S81.., S810., S811., S812., S813., S814., S815., S816., S817., S818., S81z., SD8.., SD80., SD800, SD801, SD802, SD803, SD80z, SD81., SD810, SD811, SD81z, SD82., SD8z., SE1.., SE10., SE11., SE12., SE13., SE14., SE1z., SG0.., SG00., SG01., SG02., SG020, SG03., SG0y., SG0y0, SG0z., SH0.., SH00., SH01., SH02., SH03., SH04., SH05., SH050, SH0x., SH0y., SH0z., SH102, SH112, SH122, SH12C, SH132, SH142, SH152, Syu00, Syu08, Syu09, Syu0A, Syu0B, Syu0C, Syu0M, SyuC0, SyuD0, SyuD1, SyuDC, TF4.. |
| 4 Neck | S100., S1000, S1001, S1002, S1003, S1004, S1005, S1006, S1007, S1008, S1009, S100A, S100B, S100C, S100D, S100E, S100F, S100G, S100H, S100J, S100K, S100L, S100M, S100N, S100x, S100z, S101., S1010, S1011, S1012, S1013, S1014, S1015, S1016, S1017, S1018, S1019, S101A, S101B, S101C, S101D, S101E, S101F, S101G, S101H, S101J, S101K, S101L, S101M, S101N, S101x, S101z, S10A., S10A0, S10A1, S10A2, S110., S1100, S1101, S1102, S1103, S1104, S1105, S1106, S1107, S1108, S1109, S110A, S110B, S110z, S111., S1110, S1111, S1112, S1113, S1114, S1115, S1116, S1117, S1118, S1119, S111A, S111B, S111z, S490., S4900, S4901, S4902, S4903, S4904, S4905, S4906, S4907, S4908, S4909, S490A, S490B, S490C, S490D, S490x, S490z, S491., S4910, S4911, S4912, S4913, S4914, S4915, S4916, S4917, S4918, S4919, S491A, S491B, S491C, S491D, S491x, S491z, S4965, S4975, S498., S4980, S4981, S4982, S4983, S4984, S4985, S4986, S4987, S4988, S4989, S498A, S498B, S498C, S498D, S498x, S498z, S499., S4990, S4991, S4992, S4993, S4994, S4995, S4996, S4997, S4998, S4999, S499A, S499B, S499C, S499D, S499x, S499z, S49E5, S49F5, S570., S5700, S5701, S5702, S5703, S5704, S570z, S5E1., S5N0., S5P1., S5P10, S5P11, S5P12, S5P1z, S5y2., S5y20, S5y21, S5y22, S5y23, S5y2z, S84.., S840., S8400, S8401, S8402, S840z, S841., S8410, S8411, S8412, S841z, S842., S843., S844., S845., S84x., S84x0, S84x1, S84x2, S84xz, S84y., S84y0, S84y1, S84yz, S84z., SB02., SB03., SD001, SD011, SD021, SD031, SD041, SD051, SD061, SD071, SD0y1, SD0z1, SDX.., SE07., SE08., SF02., SF020, SF021, SF022, SF02z, SG3.., SG30., SG300, SG30z, SG31., SG3z., SH109, SH119, SH129, SH12K, SH139, SH149, SH159, SJ20., SJ200, SJ201, SJ202, SJ203, SJ204, SJ205, SJ206, SJ207, SJ208, SJ209, SJ20A, SJ20B, SJ20z, SJ30., SJ300, SJ301, SJ302, SJ303, SJ304, SJ305, SJ306, SJ307, SJ40., SJ414, SJ71., SJ8.., SJ80., SJ81., SK106, SK107, SK108, SK10y, SK192, SK1x1, SK1x2, SK1x3, Syu1., Syu10, Syu11, Syu12, Syu13, Syu14, Syu15, Syu16, Syu17, Syu18, Syu19, Syu1A, Syu1B, Syu1C, Syu1D, Syu1E, Syu1F |
| 5 Thoracic / lumber spine | S102., S1020, S1021, S1022, S1023, S1024, S1025, S1026, S102y, S102z, S103., S1030, S1031, S1032, S1033, S1034, S1035, S1036, S104., S1040, S1041, S1042, S1043, S1044, S1045, S1046, S105., S1050, S1051, S1052, S1053, S1054, S1055, S1056, S10B0, S112., S1120, S1121, S1122, S1123, S1124, S1125, S1126, S1127, S1128, S1129, S112A, S112B, S112z, S113., S1130, S1131, S1132, S1133, S1134, S1135, S1136, S1137, S1138, S1139, S113A, S113B, S113z, S114., S1140, S1141, S1142, S1143, S1144, S1145, S115., S1150, S1151, S1152, S1153, S1154, S1155, S115z, S15.., S150., S1500, S1501, S492., S4920, S4921, S4922, S4923, S4924, S4925, S4926, S4927, S4928, S4929, S492A, S492B, S492C, S492z, S493., S4930, S4931, S4932, S4933, S4934, S4935, S4936, S4937, S4938, S4939, S493A, S493B, S493C, S493z, S49A., S49A0, S49A1, S49A2, S49A3, S49A4, S49A5, S49A6, S49A7, S49A8, S49A9, S49AA, S49AB, S49AC, S49Az, S49B., S49B0, S49B1, S49B2, S49B3, S49B4, S49B5, S49B6, S49B7, S49B8, S49B9, S49BA, S49BB, S49BC, S49Bz, S571., S572., S5N1., S5N2., SJ21., SJ210, SJ211, SJ212, SJ213, SJ214, SJ215, SJ216, SJ217, SJ218, SJ219, SJ21A, SJ21B, SJ21z, SJ22., SJ220, SJ221, SJ222, SJ223, SJ224, SJ225, SJ23., SJ24., SJ240, SJ241, SJ31., SJ310, SJ311, SJ312, SJ313, SJ314, SJ315, SJ316, SJ317, SJ318, SJ319, SJ31A, SJ31B, SJ32., SJ320, SJ321, SJ322, SJ323, SJ324, SJ33., SJ330, SJ331, SJ332, SJ333, SJ334, SJ35., SJ60., SJ600, SJ601, SJA0., SK190, SK191, Syu2B, Syu38 |
| 6 Chest wall | S120., S1200, S1201, S1202, S1203, S1204, S1205, S1206, S1207, S1208, S1209, S120A, S120z, S121., S1210, S1211, S1212, S1213, S1214, S1215, S1216, S1217, S1218, S1219, S121z, S122., S123., S124., S1240, S1241, S127., S1270, S1271, S128., S12X., S12X0, S12X1, S12y., S12y0, S12y1, S4960, S4962, S4963, S4964, S4966, S4967, S4970, S4972, S4973, S4974, S4976, S4977, S49E0, S49E2, S49E3, S49E4, S49E6, S49E7, S49F0, S49F2, S49F3, S49F4, S49F6, S49F7, S4J00, S4J02, S4J03, S4J10, S4J12, S4J13, S4J20, S4J22, S4J23, S4J30, S4J32, S4J33, S5E2., S5E20, S5E21, S5E2z, S5E3., S5E30, S5E31, S5E32, S5E3z, S5P2., S5P20, S5P21, S5P2z, S5P3., S5P30, S5P31, S5P32, S5P3z, S5y3., S5y30, S5y31, S5y32, S5y3z, S5y4., S5y40, S5y41, S5y42, S5y43, S5y4z, S5yX., S85.., S850., S8500, S851., S852., S853., S855., S85X., S85z., S86.., S860., S8600, S861., S86z., S890., S891., SD100, SD101, SD102, SD110, SD111, SD112, SD120, SD121, SD122, SD130, SD131, SD132, SD140, SD141, SD142, SD150, SD151, SD152, SD160, SD161, SD162, SD170, SD171, SD172, SD18., SD1y0, SD1y1, SD1y2, SD1z0, SD1z1, SD1z2, SE20., SE21., SE230, SE232, SF112, SF12., SH201, SH202, SH211, SH212, SH221, SH222, SH228, SH229, SH231, SH232, SH241, SH242, SH251, SH252, SJ42., SJ43., SJ59., SJ590, SJ591, SJ5A., SJ5A0, SJ5A1, SJ9.., SJ90., SJX.., SK110, SK111, SK112, SK1A., SK1x4, Syu2., Syu20, Syu21, Syu22, Syu23, Syu24, Syu25, Syu26, Syu27, Syu28, Syu29, Syu2A, Syu2C, Syu2D, Syu2E, Syu2F, Syu2L |
| 7 Abdominal wall | S892., S8920, S8921, S8922, S8923, S892z, S893., S8930, S8931, S8932, S8933, S893z, S894., S8940, S8941, S8942, S8943, S8944, S8945, S894z, S895., S8950, S8951, S8952, S8953, S8954, S895z, SD103, SD107, SD113, SD117, SD123, SD127, SD133, SD137, SD143, SD147, SD153, SD157, SD163, SD167, SD173, SD177, SD1y3, SD1y7, SD1z3, SD1z7, SE22., SE220, SE221, SE223, SE22z, SH203, SH213, SH223, SH22A, SH233, SH243, SH253, SJ44., SJ45., SK115, SK116 |
| 8 Internal organs | S70.., S700., S701., S702., S703., S704., S705., S706., S707., S708., S70z., S71.., S710., S7100, S7101, S7102, S7103, S710y, S710z, S711., S7110, S7111, S7112, S7113, S711y, S711z, S712., S7120, S7121, S7122, S712z, S713., S7130, S7131, S7132, S713z, S714., S7140, S7141, S715., S71z., S72.., S720., S721., S722., S7220, S7221, S7222, S7223, S7224, S722z, S723., S7230, S7231, S7232, S7233, S7234, S723z, S724., S725., S7250, S7251, S726., S727., S72x., S72y., S72z., S73.., S730., S731., S732., S7320, S7321, S732z, S733., S7330, S7331, S733z, S734., S7340, S7341, S7342, S7343, S7344, S7345, S734x, S734y, S734z, S735., S7350, S7351, S7352, S7353, S7354, S7355, S735x, S735y, S735z, S736., S737., S738., S739., S73A., S73A0, S73A1, S73B., S73x., S73x0, S73x1, S73x2, S73x3, S73x4, S73x5, S73x6, S73xy, S73xz, S73y., S73y0, S73y1, S73y2, S73y3, S73y4, S73y5, S73y6, S73yy, S73yz, S73z., S74.., S740., S7400, S7401, S7402, S7403, S7404, S740y, S740z, S741., S7410, S7411, S7412, S7413, S7414, S741y, S741z, S74z., S75.., S750., S7500, S7501, S7502, S7503, S7504, S750y, S750z, S751., S7510, S7511, S7512, S7513, S7514, S751y, S751z, S75z., S76.., S760., S7600, S7601, S7602, S7603, S760z, S761., S7610, S7611, S7612, S7613, S761z, S76z., S77.., S770., S7700, S7701, S770z, S771., S7710, S7711, S771z, S772., S773., S774., S775., S776., S777., S778., S779., S77A., S77B., S77C., S77C0, S77C1, S77v., S77v0, S77v1, S77v2, S77v3, S77v4, S77vz, S77w., S77w0, S77w1, S77w2, S77w3, S77w4, S77wz, S77x., S77y., S77z., S78.., S780., S7800, S7801, S7802, S7803, S7804, S7805, S780z, S781., S7810, S7811, S7812, S7813, S7814, S781z, S78z., S79.., S790., S791., S79z., S7A.., S7B.., S7z.., SB1.., SB10., SB11., SB12., SB120, SB121, SB13., SB14., SB15., SB150, SB151, SB16., SB160, SB161, SB162, SB16z, SB1y., SB1y0, SB1y1, SB1y2, SB1y3, SB1y4, SB1y5, SB1yx, SB1yy, SB1yz, SB1z., SB2.., SB20., SB21., SB210, SB211, SB21z, SB22., SB220, SB221, SB222, SB223, SB224, SB225, SB226, SB227, SB22z, SB23., SB230, SB231, SB232, SB233, SB234, SB235, SB23z, SB24., SB240, SB241, SB242, SB243, SB24z, SB25., SB250, SB251, SB252, SB253, SB254, SB255, SB256, SB25z, SB2y., SB2y0, SB2y1, SB2yx, SB2yz, SB2z., SG4.., SG40., SG41., SG42., SG43., SG4z., SG5.., SG50., SG51., SG52., SG5z., SG6.., SG60., SG61., SG62., SG63., SG64., SG65., SG66., SG67., SG68., SG6z., SG7.., SG70., SG71., SG7z., SG8.., SG9.., SG90., SG900, SG901, SG90z, SG91., SG92., SG920, SG921, SG92z, SG93., SG9z., SH7.., SH70., SH700, SH701, SH702, SH703, SH704, SH70z, SH71., SH710, SH711, SH712, SH713, SH714, SH71X, SH71z, SH72., SH720, SH73., SH730, SH731, SH732, SH733, SH73z, SH74., SH740, SH741, SH74z, SH7y., SH7y0, SH7z., SJ410, SJ411, SJ412, SJ413, SK05., SK1x6, SRy3., Syu2G, Syu2H, Syu2J, Syu2K, Syu3C, Syu3D, Syu3E, Syu3F, SyuC1, SyuC2, SyuC3, SyuD2, SyuD3, SyuD4, SyuD5, SyuD6, SyuD7, SyuDD, SyuDE |
| 9 Pelvis | S106., S1060, S1061, S107., S1070, S1071, S108., S109., S10B1, S10B2, S10B3, S10B4, S10B5, S116., S1160, S1161, S1162, S1163, S116z, S117., S1170, S1171, S1172, S1173, S117z, S118., S1180, S1181, S1182, S1183, S118z, S119., S1190, S1191, S1192, S1193, S119z, S13.., S130., S1300, S1301, S1302, S1303, S1304, S1305, S1306, S130y, S130z, S131., S1310, S1311, S1312, S1313, S1314, S1315, S1316, S131y, S131z, S132., S1320, S1321, S1322, S132y, S132z, S133., S1330, S1331, S1332, S133y, S133z, S134., S1340, S1341, S1342, S1343, S1344, S1345, S1346, S1347, S1348, S134z, S135., S1350, S1351, S1352, S1353, S1354, S1355, S1356, S1357, S1358, S135y, S135z, S136., S1360, S1361, S137., S1370, S1371, S138., S13y., S13z., S4941, S4942, S4951, S4952, S4961, S4971, S49C1, S49C2, S49D1, S49D2, S49E1, S49F1, S49X0, S4J01, S4J11, S4J21, S4J31, S56.., S560., S561., S5610, S5611, S562., S563., S564., S56y., S56z., S573., S5730, S5731, S573z, S574., S5M.., S5M0., S5M1., S5M2., S5M3., S5M30, S5M31, S5M3z, S5M4., S5M5., S5My., S5Mz., S5N3., S5y5., S5y50, S5y51, S5y52, S5y53, S5y54, S5y55, S5y56, S5y57, S5y5z, S87.., S870., S8700, S871., S87z., S88.., S880., S8800, S8801, S881., S882., S8820, S8821, S8822, S882z, S883., S8830, S8831, S883z, S884., S8840, S8841, S8842, S884z, S885., S8850, S8851, S8852, S885z, S886., S887., S88x., S88y., S88z., S89v0, S89v1, S89w0, S89w1, SD105, SD106, SD108, SD109, SD10A, SD10B, SD10C, SD10D, SD115, SD116, SD118, SD119, SD11A, SD11B, SD11C, SD11D, SD125, SD126, SD128, SD129, SD12A, SD12B, SD12C, SD12D, SD135, SD136, SD138, SD139, SD13A, SD13B, SD13C, SD13D, SD145, SD146, SD148, SD149, SD14A, SD14B, SD14C, SD14D, SD155, SD156, SD158, SD159, SD15A, SD15B, SD15C, SD15D, SD165, SD166, SD168, SD169, SD16A, SD16B, SD16C, SD16D, SD175, SD176, SD178, SD179, SD17A, SD17B, SD17C, SD17D, SD1y5, SD1y6, SD1y8, SD1y9, SD1yA, SD1yB, SD1yC, SD1yD, SD1z5, SD1z6, SD1z8, SD1z9, SD1zA, SD1zB, SD1zC, SD1zD, SE222, SE231, SE233, SE234, SE24., SE240, SE241, SE242, SE243, SE244, SE24z, SE25., SF10., SF100, SF101, SF102, SF10z, SF111, SF13., SH205, SH206, SH215, SH216, SH225, SH226, SH22C, SH22D, SH235, SH236, SH245, SH246, SH255, SH256, SJ350, SJ351, SJ6.., SJ6x0, SJ6x1, SJ6x2, SJ6x3, SJ6y., SJ6z., SK0y3, SK113, SK117, SK118, SK119, Syu32, TL01. |
| 10 Upper arm/ Shoulder | S20.., S200., S2000, S2001, S2002, S2003, S200z, S201., S2010, S2011, S2012, S2013, S201z, S20z., S21.., S210., S2100, S2101, S2102, S2103, S2104, S2105, S2106, S210z, S211., S2110, S2111, S2112, S2113, S2114, S2115, S2116, S211z, S21z., S22.., S220., S2200, S2201, S2202, S2203, S2204, S2205, S2206, S2207, S220z, S221., S2210, S2211, S2212, S2213, S2214, S2215, S2216, S2217, S221z, S222., S2220, S2221, S222z, S223., S2230, S2231, S223z, S224., S2241, S224x, S224z, S225., S2251, S225x, S225z, S226., S227., S228., S22z., S292., S2920, S2921, S41.., S410., S4100, S4101, S4102, S4103, S4104, S4105, S410y, S410z, S411., S4110, S4111, S4112, S4113, S4114, S4115, S411y, S411z, S412., S4120, S4121, S412z, S413., S4130, S4131, S413z, S41z., S4A.., S4A0., S4A00, S4A01, S4A1., S4A10, S4A11, S4A2., S4A20, S4A21, S4A3., S4A30, S4A31, S50.., S500., S501., S502., S503., S504., S505., S506., S507., S5070, S5071, S508., S509., S50A., S50w., S50x., S50X., S50y., S50z., S58.., S580., S581., S58z., S5F.., S5F0., S5F1., S5Fz., S5Q.., S5Q0., S5Q1., S5Q2., S5Q3., S5Q4., S5Q5., S5Q6., S5Qz., S90.., S900., S9000, S9001, S9002, S9003, S9004, S900x, S900z, S901., S9010, S9011, S9012, S9013, S901x, S901z, S902., S9020, S9021, S9022, S9023, S9024, S9025, S9026, S9027, S9028, S9029, S902x, S902z, S903., S9030, S9031, S9032, S9033, S904., S905., S906., S90z., S972., S9720, S9721, S9722, S973., SB30., SB300, SB301, SB302, SB303, SB304, SB30X, SB30z, SB31., SB310, SB311, SD2.., SD20., SD200, SD201, SD202, SD203, SD204, SD205, SD20z, SD21., SD210, SD211, SD212, SD213, SD21z, SD22., SD220, SD221, SD222, SD223, SD22z, SD23., SD230, SD231, SD232, SD233, SD23z, SD24., SD240, SD241, SD242, SD243, SD24z, SD25., SD250, SD251, SD252, SD253, SD25z, SD26., SD260, SD261, SD262, SD263, SD26z, SD27., SD270, SD271, SD272, SD273, SD27z, SD28., SD2y., SD2y0, SD2y1, SD2y2, SD2y3, SD2yz, SD2z., SD2z0, SD2z1, SD2z2, SD2z3, SD2zz, SE30., SE300, SE301, SE302, SE303, SE304, SE30y, SE30z, SF20., SF200, SF201, SF202, SF203, SF204, SF205, SF206, SF207, SF208, SF209, SF20A, SF20B, SF20C, SF20D, SF20y, SF20z, SH303, SH304, SH305, SH306, SH313, SH314, SH315, SH316, SH323, SH324, SH325, SH326, SH32A, SH32B, SH32C, SH32D, SH333, SH334, SH335, SH336, SH343, SH344, SH345, SH346, SH353, SH354, SH355, SH356, SJ34., SJ340, SJ341, SJ342, SJ343, SJ344, SJ345, SJ5.., SJ50., SJ500, SJ501, SJ54., SJ540, SJ541, SJ57., SJ570, SJ571, SJ58., SJ580, SJ581, SJ5x., SJ5y., SJ5z., SJB.., SJB0., SJB1., SJB2., SJB3., SK0y0, SK12., SK120, SK121, SK122, SK123, SK124, SK125, SK12z, Syu4., Syu40, Syu41, Syu42, Syu43, Syu44, Syu45, Syu46, Syu47, Syu48, Syu49, Syu4A, Syu4B, Syu4C, Syu4D, Syu4E |
| 11 Elbow | S2240, S2242, S2243, S2244, S2245, S2246, S2247, S2248, S2249, S2250, S2252, S2253, S2254, S2255, S2256, S2257, S2258, S2259, S2301, S2302, S2306, S2307, S230B, S2311, S2312, S2316, S2317, S231B, S42.., S420., S4200, S4201, S4202, S4203, S4204, S4205, S4206, S420y, S420z, S421., S4210, S4211, S4212, S4213, S4214, S4215, S4216, S421y, S421z, S422., S4220, S4221, S423., S4230, S4231, S424., S42z., S4B.., S4B0., S4B00, S4B01, S4B1., S4B10, S4B11, S4B2., S4B20, S4B21, S4B3., S4B30, S4B31, S510., S511., S512., S513., S51w., S51y., S59.., S590., S591., S59z., S5G.., S5G0., S5G1., S5G2., S5G3., S5Gy., S5Gz., S9101, S9111, S9121, S9131, SD300, SD310, SD320, SD330, SD340, SD350, SD360, SD370, SD3y0, SD3z0, SE311, SF211, SF213, SF215, SH302, SH312, SH322, SH329, SH332, SH342, SH352, SK130 |
| 12 Lower arm | S23.., S230., S2300, S2303, S2304, S2305, S2308, S2309, S230A, S230z, S231., S2310, S2313, S2314, S2315, S2318, S2319, S231A, S231z, S232., S2320, S2321, S2322, S2323, S232z, S233., S2330, S2331, S2332, S2333, S233z, S234., S2340, S2341, S2342, S2345, S2346, S2347, S2348, S234D, S234G, S234z, S235., S2350, S2351, S2352, S2355, S2356, S2357, S2358, S235D, S235z, S236., S237., S238., S239., S23A., S23B., S23C., S23x., S23x0, S23x1, S23x2, S23x3, S23xz, S23y., S23y0, S23y1, S23y2, S23y3, S23yz, S23z., S293., S51x., S51z., S52.., S524., S52z., S5A.., S5Az., S5H.., S5Hz., S5R.., S5Rz., S91.., S910., S9100, S910z, S911., S9110, S911z, S912., S9120, S912z, S913., S9130, S914., S915., S91z., S9202, S970., S9700, S9701, S9702, S970X, S971., S97X., SB32., SB320, SB321, SB322, SB33., SB330, SB331, SB332, SB36., SB360, SB3W., SB3X., SB3x0, SD3.., SD30., SD301, SD30z, SD31., SD311, SD31z, SD32., SD321, SD32z, SD33., SD331, SD33z, SD34., SD341, SD34z, SD35., SD351, SD35z, SD36., SD361, SD363, SD36z, SD37., SD371, SD37z, SD38., SD39., SD3y., SD3y1, SD3yz, SD3z., SD3z1, SD3zz, SE31., SE310, SE31z, SE32., SE32z, SF21., SF210, SF212, SF214, SF21z, SF22., SF22z, SH301, SH311, SH321, SH328, SH331, SH341, SH351, SH4.., SH40., SH40x, SH40z, SH41., SH41x, SH41z, SH42., SH42x, SH42z, SH43., SH43x, SH43z, SH44., SH44x, SH44z, SH45., SH45x, SH45z, SH46., SH460, SH461, SH462, SH4z., SJ52., SJ520, SJ521, SJ522, SJ523, SJ524, SJ525, SJ526, SJ527, SJ528, SJ53., SJ530, SJ531, SJ532, SJ533, SJ534, SJ5B., SJ5B0, SJ5B1, SJ5C., SJ5C0, SJ5C1, SJ5X., SJ5y0, SJ5y1, SJW.., SK0y1, SK13., SK131, SK13z, SK1C., SK1C0, SK1C1, SK1C2, SK1C3, SK1C4, SK1C5, SK1CW, SK1CX, SK1x7, Syu5., Syu50, Syu51, Syu52, Syu53, Syu54, Syu55, Syu56, Syu57, Syu58, Syu59, Syu5A, Syu5B, Syu5C, Syu5D, Syu5E, Syu5F, Syu5G, Syu6., Syu60, Syu61, Syu62, Syu65, Syu66, Syu67, Syu68, Syu69, Syu6A, Syu6B, Syu6C, Syu6D, Syu6E, Syu6F, Syu6H, Syu6J, Syu6K, Syu6L, Syu6M |
| 13 Wrist | S2343, S2344, S2349, S234A, S234B, S234C, S234E, S234F, S2353, S2354, S2359, S235A, S235B, S235C, S235E, S235F, S43.., S430., S4300, S4301, S4302, S4303, S4304, S4305, S4306, S4307, S4308, S430y, S430z, S431., S4310, S4311, S4312, S4313, S4314, S4315, S4316, S4317, S4318, S431y, S431z, S432., S4320, S4321, S4322, S4323, S4324, S4325, S4326, S432y, S433., S4330, S4331, S4332, S4333, S4334, S4335, S4336, S433y, S43z., S4C.., S4C0., S4C00, S4C01, S4C02, S4C03, S4C04, S4C05, S4C06, S4C0y, S4C1., S4C10, S4C11, S4C12, S4C13, S4C14, S4C15, S4C16, S4C1y, S4C2., S4C20, S4C21, S4C22, S4C23, S4C24, S4C25, S4C26, S4C2y, S4C3., S4C30, S4C31, S4C32, S4C33, S4C34, S4C35, S4C36, S4C3y, S520., S5200, S5201, S5202, S5203, S5204, S5205, S5206, S5207, S5208, S5209, S520A, S520B, S520C, S520D, S520E, S520F, S520G, S520H, S520J, S520K, S520L, S520M, S520z, S5240, S5241, S5A0., S5A00, S5A01, S5A02, S5A03, S5A04, S5A05, S5A06, S5A07, S5A08, S5A09, S5A0A, S5A0B, S5A0C, S5A0D, S5A0E, S5A0F, S5A0G, S5A0H, S5A0J, S5A0z, S5H0., S5H00, S5H01, S5H0z, S5R0., S5R1., S9102, S9103, S9104, S9112, S9122, S9123, S9124, S9125, S9126, S9132, S9133, SD302, SD303, SD312, SD322, SD332, SD342, SD352, SD362, SD372, SD3y2, SD3z2, SE321, SE324, SE325, SF221, SF222, SF223, SF226, SF227, SH407, SH417, SH427, SH42F, SH437, SH447, SH457, SK132, SK133, TK601 |
| 14 Hand | S24.., S240., S2400, S2401, S2402, S2403, S2404, S2405, S2406, S2407, S2408, S2409, S240A, S240B, S240C, S240D, S240E, S240F, S240y, S240z, S241., S2410, S2411, S2412, S2413, S2414, S2415, S2416, S2417, S2418, S2419, S241A, S241B, S241C, S241D, S241E, S241F, S241y, S241z, S242., S2420, S2421, S2422, S2423, S24z., S25.., S250., S2500, S2501, S2502, S2503, S2504, S2505, S2506, S2507, S2508, S2509, S250A, S250B, S250C, S250x, S250z, S251., S2510, S2511, S2512, S2513, S2514, S2515, S2516, S2517, S2518, S2519, S251A, S251B, S251C, S251x, S251z, S252., S253., S27.., S270., S271., S27z., S2B.., S521., S5210, S5211, S5212, S5214, S521z, S92.., S920., S9200, S9201, S921., S922., S9220, S9221, S9222, S9223, S923., S9230, S9231, S924., S925., S92z., S9703, SB34., SB340, SB341, SB342, SB343, SD4.., SD40., SD400, SD401, SD41., SD42., SD43., SD44., SD45., SD46., SD460, SD47., SD4y., SD4z., SE320, SE322, SE323, SF220, SF224, SF225, SF228, SF229, SH400, SH405, SH406, SH410, SH415, SH416, SH420, SH425, SH426, SH428, SH42D, SH42E, SH430, SH435, SH436, SH440, SH445, SH446, SH450, SH455, SH456, SK06., SK0y2, SK14., SK140, SR40., Syu63, Syu64 |
| 15 Fingers | S26.., S260., S2600, S2601, S2602, S2603, S2604, S2605, S2606, S2607, S2608, S2609, S260A, S260B, S260C, S260D, S260E, S260F, S260G, S260H, S260J, S260K, S260L, S260M, S260N, S260P, S260Q, S260R, S260S, S260T, S260U, S260V, S260W, S260x, S260z, S261., S2610, S2611, S2612, S2613, S2614, S2615, S2616, S2617, S2618, S2619, S261A, S261B, S261C, S261D, S261E, S261F, S261G, S261H, S261J, S261K, S261L, S261M, S261N, S261P, S261Q, S261R, S261S, S261T, S261U, S261V, S261W, S261x, S261z, S262., S263., S264., S26z., S44.., S440., S4400, S4401, S4402, S4403, S4404, S4405, S4406, S440z, S441., S4410, S4411, S4412, S4413, S4414, S4415, S4416, S441z, S442., S4420, S4421, S4422, S4423, S4424, S4425, S443., S4430, S4431, S4432, S4433, S4434, S4435, S44z., S4D.., S4D0., S4D00, S4D01, S4D02, S4D03, S4D04, S4D05, S4D06, S4D1., S4D10, S4D11, S4D12, S4D13, S4D14, S4D15, S4D16, S4D2., S4D20, S4D21, S4D22, S4D23, S4D24, S4D25, S4D26, S4D3., S4D30, S4D31, S4D32, S4D33, S4D34, S4D35, S4D36, S5213, S522., S5220, S5221, S5222, S5223, S5224, S5225, S5226, S5227, S523., S5230, S5231, S5232, S5233, S5234, S5235, S5236, S5237, S5238, S5239, S523A, S523B, S523C, S523D, S523E, S523F, S525., S5250, S5251, S526., S5260, S5261, S5262, S5A1., S5A10, S5A11, S5A12, S5A13, S5A1z, S5A2., S5A20, S5A21, S5A22, S5A23, S5A24, S5A25, S5A2z, S5H1., S5H10, S5H11, S5H12, S5H13, S5H1z, S5H2., S5H20, S5H21, S5H22, S5H23, S5H24, S5H25, S5H2z, S5S.., S5S0., S5S1., S5S2., S5Sz., S5T.., S5T0., S5T1., S5T2., S5T3., S5Tz., S93.., S930., S9300, S9301, S9302, S931., S932., S9320, S9321, S9322, S9323, S9324, S9325, S9326, S9327, S9328, S9329, S932A, S932B, S932C, S932D, S933., S9330, S9331, S9332, S9333, S9334, S9335, S934., S935., S936., S937., S938., S93z., S95.., S950., S9500, S9501, S9502, S9503, S9504, S9505, S9506, S9507, S9508, S951., S95z., S96.., S960., S9600, S9601, S9602, S9603, S9604, S9605, S9606, S9607, S9608, S9609, S960A, S961., S96z., SB35., SB350, SB351, SB352, SB353, SB354, SB355, SB3y0, SD5.., SD50., SD500, SD501, SD502, SD51., SD52., SD53., SD54., SD55., SD56., SD57., SD5y., SD5z., SE33., SE330, SE331, SE332, SE333, SE33z, SF23., SF230, SF231, SF232, SF233, SF234, SF235, SH401, SH402, SH403, SH404, SH411, SH412, SH413, SH414, SH421, SH422, SH423, SH424, SH429, SH42A, SH42B, SH42C, SH431, SH432, SH433, SH434, SH441, SH442, SH443, SH444, SH451, SH452, SH453, SH454, SJ56., SJ560, SJ561, SJ562, SJ563, SJ564, SJ565, SJ566, SK15., SK150, SK151, SK152, SK153, SK154, SK15z, Syu6G |
| 16 Hip | S30.., S300., S3000, S3001, S3002, S3003, S3004, S3005, S3006, S3007, S3008, S3009, S300A, S300y, S300z, S301., S3010, S3011, S3012, S3013, S3014, S3015, S3016, S3017, S3018, S3019, S301A, S301y, S301z, S302., S3020, S3021, S3024, S302z, S303., S3030, S3031, S3033, S3034, S303z, S304., S30y., S30z., S45.., S450., S4500, S4501, S4502, S4503, S450z, S451., S4510, S4511, S4512, S4513, S451z, S452., S4520, S4521, S4522, S453., S4530, S4531, S4532, S45z., S4E.., S4E0., S4E1., S4E2., S4E3., S530., S531., S532., S53w., S53y., S5B.., S5B0., S5By., S5Bz., S5J.., S5J0., S5Jy., S5Jz., SA000, SA010, SA020, SA030, SD600, SD610, SD620, SD630, SD640, SD650, SD660, SD670, SD6y0, SD6z0, SE400, SF301, SF304, SF305, SK160 |
| 17 Upper leg | S3022, S3023, S3032, S305., S30w., S30x., S31.., S310., S3100, S3101, S310z, S311., S3110, S3111, S311z, S312., S3120, S3121, S3123, S312z, S313., S3130, S3133, S313z, S314., S315., S31z., S3x2., S53.., S533., S535., S53x., S53z., S5U.., S5U0., S5U1., S5U2., S5Uz., SA0.., SA00., SA001, SA00z, SA01., SA011, SA01z, SA02., SA021, SA022, SA023, SA024, SA025, SA02z, SA03., SA031, SA04., SA05., SA0z., SA72., SA720, SA721, SA722, SA73., SA78., SA9.., SB40., SB400, SB401, SB41., SB410, SB411, SB42., SB420, SB421, SB4X., SB4x0, SD601, SD611, SD621, SD631, SD641, SD651, SD661, SD671, SD68., SD6y1, SD6z1, SE40., SE401, SE40z, SF30., SF300, SF302, SF303, SF30z, SH506, SH516, SH526, SH52D, SH536, SH546, SH556, SJ61., SJ610, SJ611, SJ642, SJ643, SJ65., SJ6X., SK0y4, SK16., SK161, SK16z, SK1D., SK1D0, SK1D1, Syu7., Syu70, Syu71, Syu72, Syu73, Syu74, Syu75, Syu76, Syu77, Syu78 |
| 18 Knee | S3122, S3124, S3125, S3126, S312x, S3131, S3132, S3134, S3135, S3136, S313x, S32.., S320., S3200, S3201, S3202, S3203, S3204, S321., S3210, S3211, S3212, S3213, S3214, S32z., S3303, S3304, S3305, S3306, S3307, S3313, S3314, S3315, S3316, S3317, S331A, S46.., S460., S4600, S4601, S4602, S4603, S4604, S4605, S461., S4610, S4611, S4612, S4613, S4614, S4615, S462., S463., S4630, S4631, S464., S4640, S4641, S465., S4650, S4651, S4652, S4653, S4654, S4655, S4656, S465z, S466., S4660, S4661, S4662, S4663, S4664, S4665, S4666, S466z, S467., S4670, S4671, S468., S4680, S4681, S469., S4690, S4691, S4692, S4693, S4694, S4695, S4696, S46A., S46A0, S46A1, S46A2, S46A3, S46A4, S46A5, S46A6, S46B., S46C., S46D., S46z., S4F.., S4F0., S4F1., S4F2., S4F3., S4F4., S4F5., S4F6., S4F7., S534., S540., S5400, S5401, S541., S5410, S5411, S542., S5421, S5422, S543., S545., S54w., S54y., S5C.., S5C0., S5C1., S5C2., S5C3., S5Cy., S5Cz., S5K.., S5K0., S5K1., S5K2., S5K3., S5K4., S5Ky., S5Kz., SA100, SA110, SA120, SA123, SA125, SA130, SB44., SB440, SB441, SB442, SB443, SB444, SB445, SB446, SB44z, SD602, SD612, SD622, SD632, SD642, SD652, SD662, SD665, SD672, SD6y2, SD6z2, SE411, SF311, SF313, SF315, SH505, SH515, SH525, SH52C, SH535, SH545, SH555, SK170, SK176, SK177, Syu84 |
| 19 Lower leg | S33.., S330., S3300, S3301, S3302, S3308, S3309, S330z, S331., S3310, S3311, S3312, S3318, S3319, S331z, S332., S3320, S3321, S3322, S332z, S333., S3330, S3331, S3332, S333z, S334., S3340, S335., S3350, S336., S3360, S337., S338., S339., S3390, S3391, S33A., S33B., S33C., S33x., S33x0, S33x1, S33x2, S33xz, S33y., S33y0, S33y1, S33y2, S33yz, S33z., S3X.., S3x3., S544., S54x1, S55.., S55z., S5D.., S5Dz., S5L.., S5Lz., S5V1., SA111, SA121, SA131, SA14., SA15., SA16., SA70., SA701, SA702, SA71., SA7X., SB45., SB450, SB451, SB452, SB453, SB454, SB455, SB456, SB457, SB458, SB45z, SB47., SB470, SB471, SB472, SB473, SB48., SB481, SB482, SB483, SB4W., SD603, SD613, SD623, SD633, SD643, SD653, SD663, SD673, SD69., SD6A., SD6y3, SD6z3, SE41., SE410, SE41z, SE42., SE42z, SF31., SF310, SF312, SF314, SF31z, SF32., SF32z, SH504, SH514, SH524, SH52B, SH534, SH544, SH554, SH57., SH570, SH571, SH572, SJ62., SJ620, SJ621, SJ63., SJ630, SJ631, SJ632, SJ633, SJ634, SJ635, SJ66., SJ660, SJ66X, SJ67., SJ670, SJ671, SJ672, SJ673, SJ674, SJ67X, SK174, SK175, SK1E., SK1E0, SK1E1, SK1E2, SK1EX, SK1F., SK1F0, SK1F1, SK1FX, SK1x8, Syu8., Syu80, Syu81, Syu82, Syu83, Syu85, Syu86, Syu87, Syu88, Syu89, Syu8A, Syu8B, Syu8C, Syu8D, Syu8E, Syu8F, Syu9., Syu91, Syu93, Syu97, Syu98, Syu99, Syu9A, Syu9B, Syu9C, Syu9D, Syu9G |
| 20 Ankle | S3341, S3351, S34.., S340., S341., S342., S3420, S3421, S343., S3430, S3431, S344., S3440, S3441, S345., S3450, S3451, S346., S3460, S3461, S347., S3470, S3471, S348., S349., S34x., S34y., S34z., S47.., S470., S471., S472., S473., S47z., S4G.., S4G0., S4G1., S4G2., S4G3., S550., S5500, S5501, S5502, S5503, S5504, S5505, S5506, S550z, S5D0., S5D00, S5D01, S5D0z, S5L0., S5L00, S5L01, S5L02, S5L03, S5L0z, S5V0., SA102, SA112, SA122, SA124, SA126, SA132, SD604, SD614, SD624, SD634, SD644, SD654, SD664, SD674, SD6y4, SD6z4, SE421, SF321, SF323, SF325, SH503, SH513, SH523, SH52A, SH533, SH543, SH553, SK172 |
| 21 Foot | S35.., S350., S3500, S3501, S351., S3510, S3511, S352., S3520, S3521, S3522, S3523, S3524, S3525, S3526, S3527, S3528, S3529, S352A, S352B, S352C, S352D, S352E, S352F, S352G, S352H, S352J, S352z, S353., S3530, S3531, S3532, S3533, S3534, S3535, S3536, S3537, S3538, S3539, S353A, S353B, S353C, S353D, S353E, S353F, S353G, S353H, S353J, S353z, S354., S355., S356., S35z., S3x4., S48.., S480., S4800, S4801, S4802, S4803, S4804, S4805, S4807, S4809, S480A, S480z, S481., S4810, S4811, S4812, S4813, S4814, S4815, S4817, S4819, S481A, S481z, S482., S4820, S4821, S4822, S4823, S4824, S4826, S4828, S4829, S483., S4830, S4831, S4832, S4833, S4834, S4836, S4838, S4839, S48z., S4H.., S4H0., S4H00, S4H01, S4H02, S4H03, S4H05, S4H1., S4H10, S4H11, S4H12, S4H13, S4H15, S4H2., S4H20, S4H21, S4H22, S4H23, S4H25, S4H3., S4H30, S4H31, S4H32, S4H33, S4H35, S551., S5510, S5511, S5512, S5514, S5515, S5516, S551z, S5D1., S5D10, S5D11, S5D12, S5D1z, S5L1., S5L10, S5L11, S5L12, S5L1z, S5V2., S5V3., SA2.., SA20., SA201, SA202, SA203, SA21., SA22., SA220, SA221, SA222, SA223, SA23., SA230, SA231, SA232, SA24., SA25., SA2z., SA6.., SA60., SA600, SA61., SA62., SA63., SA6z., SB46., SB460, SB461, SB480, SB4y0, SB4y1, SD7.., SD70., SD700, SD70z, SD71., SD710, SD71z, SD72., SD720, SD722, SD72z, SD73., SD730, SD73z, SD74., SD740, SD74z, SD75., SD750, SD75z, SD76., SD760, SD762, SD763, SD76z, SD77., SD770, SD77z, SD7y., SD7y0, SD7yz, SD7z., SD7z0, SD7zz, SE420, SF320, SF322, SF324, SH502, SH512, SH522, SH529, SH532, SH542, SH552, SK0y6, SK173, SR41., Syu90, Syu92, Syu94, Syu95, Syu96, Syu9F |
| 22 Toes | S36.., S360., S3600, S3601, S3602, S3603, S361., S3610, S3611, S3612, S3613, S362., S3620, S3621, S363., S36z., S4806, S4808, S4816, S4818, S4825, S4827, S4835, S4837, S4H04, S4H06, S4H14, S4H16, S4H24, S4H26, S4H34, S4H36, S5513, S5D13, S5L13, SA3.., SA30., SA31., SA32., SA33., SA330, SA331, SA34., SA3z., SA5.., SA50., SA500, SA501, SA502, SA51., SA5z., SD701, SD711, SD721, SD731, SD741, SD751, SD761, SD771, SD7y1, SD7y2, SD7z1, SE43., SF33., SH501, SH511, SH521, SH528, SH531, SH541, SH551, Syu9E |
| 23 Multiple body parts | S29.., S290., S291., S29z., S3y.., S3y0., S3y1., S3yz., S49.., S7..., S8..., S8z.., S96X., SAA.., SD9.., SDA.., SDC.., SDC1., SE4.., SE4y., SF4.., SF40., SH6.., SH60., SH61., SH62., SH620, SH621, SH63., SH64., SH65., SH66., SH660, SH661, SH663, SH6z., SJ2x., SJ3x., SJA.., SK1xA, SQ..., SR..., SR1.., SR15., SR150, SR151, SR16., SR160, SR161, SR1z., SR1z0, SR1z1, SR2.., SR24., SR3.., SR31., SR4.., SR42., SR43., SR44., SRy.., SRy1., SRy2., SRz.., SRz0., SyuA., SyuA0, SyuA1, SyuA2, SyuA3, SyuA4, SyuA5, SyuA6, SyuA7, SyuA8, SyuA9, SyuAA, SyuD8, SyuD9 |
| 24 Head/face/neck | S8A.., SB0.., SB00., SB000, SB001, SB002, SB003, SB00z, SB01., SB0y., SB0y0, SB0y1, SB0yz, SB0z., SD0yz, SD0z., SDC0., SE0.., SE0z., SF0.., SF0z., SH1.., SH10., SH100, SH10x, SH10z, SH11., SH110, SH11x, SH11z, SH12., SH120, SH12A, SH12x, SH12z, SH13., SH130, SH13A, SH13x, SH13z, SH14., SH140, SH14x, SH14z, SH15., SH150, SH15x, SH15z, SH16., SH160, SH161, SH1z., SJ14., SJ15., SJ17., SJ18., SJ1y0, SJ1y1, SJ70., SK10., SK10z, SR10., SR100, SR101, SR20., SR30., SRy0., TGyz2, TM81. |
| 25 Arm | S2..., S28.., S280., S281., S28z., S294., S2940, S2941, S2A.., S2z.., S51.., S9..., S94.., S940., S941., S942., S94z., S97.., S974., S975., S976., S977., S97z., S9z.., SB3.., SB3x., SB3y., SB3z., SE3.., SE3y., SE3z., SF2.., SF2y., SF2z., SH3.., SH30., SH300, SH30x, SH30z, SH31., SH310, SH31x, SH31z, SH32., SH320, SH327, SH32x, SH32z, SH33., SH330, SH33x, SH33z, SH34., SH340, SH34x, SH34z, SH35., SH350, SH35x, SH35z, SH36., SH360, SH361, SH362, SH3z., SJ51., SJ510, SJ511, SJ512, SJ513, SJ514, SJ515, SJ55., SR12., SR120, SR121, SR22., SyuB2, SyuBK, SyuBL, SyuBM, SyuBN |
| 26 Leg | S3..., S37.., S370., S371., S3x.., S3x0., S3x1., S3xz., S54.., S54x., S54z., S5V.., S5Vz., SA..., SA1.., SA10., SA101, SA10z, SA11., SA11z, SA12., SA12z, SA13., SA1z., SA4.., SA40., SA41., SA42., SA4z., SA7.., SA74., SA75., SA76., SA77., SA7z., SAz.., SB4.., SB43., SB430, SB431, SB43z, SB4x., SB4y., SB4z., SD6.., SD60., SD60z, SD61., SD61z, SD62., SD62z, SD63., SD63z, SD64., SD64z, SD65., SD65z, SD66., SD66z, SD67., SD67z, SD6y., SD6yz, SD6z., SD6zz, SE44., SE45., SF3.., SF3y., SF3z., SH5.., SH50., SH500, SH50x, SH50z, SH51., SH510, SH51x, SH51z, SH52., SH520, SH527, SH52x, SH52z, SH53., SH530, SH53x, SH53z, SH54., SH540, SH54x, SH54z, SH55., SH550, SH55x, SH55z, SH56., SH560, SH561, SH562, SH5z., SJ64., SJ640, SJ641, SJ6W., SJ6x., SK0y5, SK17., SK171, SK17z, SK1X., SR13., SR14., SR140, SR141, SR23., SyuB3, SyuB4, SyuB5, SyuB6, SyuB7, SyuB8 |
| 27 Trunk | S1..., S10B., S10B6, S14.., S140., S141., S14z., S49X., S57.., S57X., S57z., S57z0, S5N.., S5Nz., S896., S89v., S89vz, S89w., S89wz, SD1.., SD10., SD104, SD10z, SD11., SD114, SD11z, SD12., SD124, SD12z, SD13., SD134, SD13z, SD14., SD144, SD14z, SD15., SD154, SD15z, SD16., SD164, SD16z, SD17., SD174, SD17z, SD1y., SD1y4, SD1yz, SD1z., SD1z4, SD1zz, SE2.., SE23., SE23z, SE2y., SE2z., SF1.., SF11., SF110, SF11X, SF11z, SF1y., SF1z., SH2.., SH20., SH200, SH204, SH20x, SH20z, SH21., SH210, SH214, SH217, SH21x, SH21z, SH22., SH220, SH224, SH227, SH22B, SH22E, SH22x, SH22z, SH23., SH230, SH234, SH237, SH23x, SH23z, SH24., SH240, SH244, SH24x, SH24z, SH25., SH250, SH254, SH25x, SH25z, SH26., SH2z., SJ4.., SJ415, SJ4y., SJ4z., SK11., SK114, SK11z, SK1B., SK1W., SK1x5, SK1x9, SR11., SR21., SR32., Syu3., Syu30, Syu31, Syu33, Syu34, Syu35, Syu36, Syu39, Syu3A, Syu3B, Syu3G, Syu3H, Syu3K, Syu3L, Syu3M, SyuB0, SyuB1, SyuBJ |
| 98 Other | S10.., S10x., S10y., S10z., S11.., S11x., S11y., S11z., S12.., S125., S1250, S1251, S1252, S1253, S125z, S126., S1260, S1261, S1262, S1263, S126z, S12z., S1z.., S494., S4940, S494z, S495., S4950, S495z, S496., S49C., S49C0, S49Cz, S49D., S49D0, S49Dz, S49E., SJ2z., SJ3.., SJ41., SJ41z, SJ7x., SJ7x0, SJ7y., SJ7z., SK19., SL..., SL0.., SL00., SL000, SL001, SL002, SL003, SL00z, SL01., SL010, SL011, SL012, SL013, SL01z, SL02., SL020, SL021, SL02z, SL03., SL030, SL031, SL032, SL03z, SL04., SL040, SL041, SL042, SL043, SL04z, SL05., SL050, SL051, SL052, SL053, SL05z, SL06., SL060, SL061, SL062, SL063, SL06z, SL07., SL070, SL071, SL072, SL073, SL074, SL07z, SL0y., SL0z., SL1.., SL10., SL100, SL101, SL102, SL10z, SL11., SL12., SL120, SL121, SL122, SL123, SL12z, SL13., SL130, SL131, SL13z, SL14., SL140, SL141, SL142, SL143, SL144, SL145, SL14z, SL15., SL150, SL15z, SL16., SL160, SL161, SL162, SL16z, SL17., SL170, SL17z, SL1x., SL1x0, SL1x1, SL1x2, SL1x3, SL1x4, SL1xz, SL1y., SL1y0, SL1y1, SL1yz, SL1z., SL2.., SL20., SL200, SL201, SL202, SL203, SL20z, SL21., SL210, SL211, SL212, SL213, SL21z, SL22., SL220, SL221, SL222, SL223, SL22z, SL23., SL230, SL231, SL232, SL233, SL234, SL235, SL236, SL237, SL23z, SL24., SL240, SL241, SL242, SL24z, SL25., SL250, SL25z, SL26., SL27., SL270, SL271, SL272, SL273, SL27z, SL28., SL280, SL281, SL282, SL28z, SL29., SL2y., SL2z., SL3.., SL30., SL300, SL301, SL302, SL303, SL304, SL30x, SL30z, SL31., SL310, SL311, SL312, SL313, SL314, SL315, SL316, SL317, SL31z, SL32., SL33., SL34., SL340, SL34z, SL35., SL350, SL351, SL35z, SL3y., SL3y0, SL3yz, SL3z., SL4.., SL40., SL400, SL401, SL40x, SL40z, SL41., SL410, SL41z, SL42., SL420, SL421, SL422, SL423, SL424, SL42z, SL43., SL44., SL440, SL441, SL442, SL443, SL44z, SL45., SL450, SL451, SL45z, SL46., SL47., SL470, SL471, SL472, SL473, SL47z, SL4y., SL4y0, SL4y1, SL4yz, SL4z., SL5.., SL50., SL500, SL501, SL502, SL503, SL504, SL505, SL506, SL507, SL50z, SL51., SL510, SL511, SL51z, SL52., SL520, SL521, SL522, SL52z, SL53., SL530, SL531, SL53z, SL54., SL540, SL541, SL542, SL543, SL544, SL54z, SL5x., SL5x0, SL5xz, SL5y., SL5y0, SL5y1, SL5y2, SL5yz, SL5z., SL6.., SL60., SL600, SL601, SL60z, SL61., SL610, SL61z, SL62., SL620, SL621, SL62z, SL6x., SL6x0, SL6x1, SL6xz, SL6y., SL6y0, SL6y1, SL6y2, SL6y3, SL6yz, SL6z., SL7.., SL70., SL700, SL701, SL702, SL703, SL704, SL705, SL70z, SL71., SL72., SL73., SL730, SL731, SL73z, SL74., SL75., SL76., SL7y., SL7z., SL8.., SL80., SL800, SL801, SL802, SL80z, SL81., SL82., SL820, SL821, SL822, SL82z, SL83., SL830, SL831, SL832, SL83x, SL83z, SL84., SL85., SL850, SL851, SL852, SL853, SL85z, SL86., SL87., SL8z., SL9.., SL90., SL900, SL901, SL902, SL903, SL90z, SL91., SL910, SL911, SL912, SL913, SL914, SL91z, SL92., SL920, SL921, SL922, SL92z, SL93., SL94., SL940, SL941, SL942, SL943, SL944, SL945, SL946, SL94z, SL95., SL950, SL951, SL95z, SL96., SL960, SL961, SL962, SL963, SL964, SL96z, SL97., SL970, SL971, SL972, SL97z, SL9y., SL9z., SLA.., SLA0., SLA00, SLA01, SLA0z, SLA1., SLA10, SLA11, SLA12, SLA1z, SLAy., SLAz., SLB.., SLB0., SLB00, SLB01, SLB02, SLB0z, SLB1., SLB10, SLB11, SLB12, SLB13, SLB14, SLB1z, SLB2., SLB20, SLB21, SLB2z, SLB3., SLB30, SLB31, SLB3z, SLBz., SLC.., SLC0., SLC00, SLC01, SLC02, SLC03, SLC04, SLC0z, SLC1., SLC10, SLC11, SLC12, SLC1z, SLC2., SLC20, SLC21, SLC2z, SLC3., SLC30, SLC3z, SLC4., SLC40, SLC41, SLC42, SLC4z, SLC5., SLC50, SLC51, SLC52, SLC5z, SLC6., SLC60, SLC61, SLC62, SLC63, SLC64, SLC6z, SLC7., SLC70, SLC71, SLC7z, SLC8., SLC80, SLC81, SLC8z, SLC9., SLCz., SLD.., SLD0., SLD00, SLD01, SLD02, SLD0z, SLD1., SLD10, SLD11, SLD12, SLD1z, SLD2., SLD20, SLD2z, SLD3., SLD30, SLD31, SLD3z, SLD4., SLD40, SLD41, SLD42, SLD4z, SLD5., SLD50, SLD51, SLD5z, SLD6., SLDy., SLDz., SLE.., SLE0., SLE00, SLE01, SLE02, SLE0z, SLE1., SLE10, SLE11, SLE1z, SLE2., SLE20, SLE2z, SLE3., SLE30, SLE31, SLE3z, SLE4., SLE40, SLE41, SLE4z, SLE5., SLE6., SLE7., SLE70, SLE71, SLE72, SLE7z, SLEz., SLF.., SLF0., SLF00, SLF01, SLF02, SLF0z, SLF1., SLF10, SLF11, SLF1z, SLF2., SLF3., SLF4., SLF40, SLF41, SLF4z, SLF5., SLF50, SLF51, SLF52, SLF5z, SLF6., SLF7., SLF70, SLF71, SLF7z, SLFy., SLFz., SLG.., SLG0., SLG1., SLG2., SLG3., SLG4., SLG5., SLG50, SLG5z, SLG6., SLG7., SLGx., SLGx0, SLGxz, SLGz., SLH.., SLH0., SLH00, SLH0z, SLH1., SLH2., SLH3., SLH4., SLHy., SLHy0, SLHy1, SLHyz, SLHz., SLJ.., SLJ0., SLJ1., SLJ10, SLJ11, SLJ1z, SLJ2., SLJ3., SLJ4., SLJ5., SLJ6., SLJx., SLJy., SLJz., SLK.., SLK0., SLK1., SLK2., SLK3., SLK4., SLK5., SLK6., SLK60, SLK6z, SLK7., SLK8., SLKz., SLX.., SLz.., SM..., SM0.., SM00., SM000, SM001, SM002, SM00z, SM01., SM010, SM011, SM01z, SM02., SM020, SM021, SM022, SM02z, SM03., SM030, SM031, SM032, SM03z, SM0y., SM0z., SM1.., SM10., SM11., SM12., SM13., SM14., SM15., SM1z., SM2.., SM20., SM21., SM22., SM23., SM230, SM231, SM232, SM23z, SM24., SM2y., SM2y0, SM2y1, SM2yz, SM2z., SM3.., SM30., SM300, SM301, SM30z, SM31., SM310, SM311, SM312, SM31z, SM32., SM320, SM321, SM322, SM32z, SM3z., SM4.., SM40., SM400, SM401, SM40z, SM41., SM410, SM411, SM41z, SM4y., SM4z., SM5.., SM50., SM51., SM52., SM53., SM54., SM55., SM56., SM57., SM58., SM5y., SM5y0, SM5y1, SM5y2, SM5y3, SM5yz, SM5z., SM6.., SM7.., SM70., SM700, SM701, SM70z, SM71., SM72., SM720, SM721, SM72z, SM73., SM74., SM75., SM750, SM751, SM752, SM75z, SM76., SM77., SM78., SM79., SM7A., SM7y., SM7y0, SM7y1, SM7y2, SM7yz, SM7z., SM8.., SM80., SM800, SM801, SM80W, SM80X, SM81., SM82., SM8y., SM8z., SM9.., SM90., SM900, SM901, SM90z, SM91., SM92., SM920, SM921, SM922, SM923, SM92z, SM93., SM930, SM931, SM932, SM933, SM934, SM935, SM93z, SM94., SM95., SM950, SM951, SM952, SM953, SM954, SM955, SM956, SM95z, SM96., SM97., SM98., SM9A., SM9B., SM9B0, SM9B1, SM9C., SM9X., SM9y., SM9z., SMB.., SMC.., SMX.., SMz.., SN41., SN410, SN412, SN413, SN41z, SN47., SN470, SN471, SN472, SN473, SN474, SN475, SN476, SN47z, SyuF., SyuF0, SyuF1, SyuF2, SyuF3, SyuF4, SyuF5, SyuF6, SyuF7, SyuF8, SyuF9, SyuFa, SyuFA, SyuFb, SyuFB, SyuFc, SyuFC, SyuFd, SyuFD, SyuFe, SyuFE, SyuFF, SyuFG, SyuFH, SyuFJ, SyuFK, SyuFL, SyuFM, SyuFN, SyuFP, SyuFQ, SyuFR, SyuFS, SyuFT, SyuFU, SyuFV, SyuFW, SyuFX, SyuFY, SyuFZ, SyuG., SyuG0, SyuG1, SyuG2, SyuG3, SyuG4, SyuG5, SyuG6, SyuG7, SyuG8, SyuG9, SyuGA, SyuGB, SyuGC, SyuGD, SyuGE, SyuGF, SyuGG, SyuGH, SyuGJ, SyuGK, SyuGL, SyuGM, T180., T1800, T1801, T1802, T1803, T1804, T1805, T1806, T1807, T180y, T180z, T250., T2500, T2501, T2502, T2503, T2504, T2505, T2506, T2507, T250y, T250z, T40.., T400., T4000, T4001, T4002, T4003, T4004, T4005, T4006, T400y, T400z, T401., T4010, T4011, T4012, T4013, T4014, T4015, T4016, T401y, T401z, T402., T4020, T4021, T4022, T4023, T4024, T4025, T4026, T402y, T402z, T403., T4030, T4031, T4032, T4033, T4034, T4035, T4036, T403y, T403z, T404., T4040, T4041, T4042, T4043, T4044, T4045, T4046, T404y, T404z, T405., T4050, T4051, T4052, T4053, T4054, T4055, T4056, T405y, T405z, T406., T4060, T4061, T4062, T4063, T4064, T4065, T4066, T406y, T406z, T40z., T40z0, T40z1, T40z2, T40z3, T40z4, T40z5, T40z6, T40zy, T40zz, T42.., T420., T4200, T4201, T4202, T4203, T4204, T4205, T4206, T420y, T420z, T421., T4210, T4211, T4212, T4213, T4214, T4215, T4216, T421y, T421z, T422., T4220, T4221, T4222, T4223, T4224, T4225, T4226, T422y, T422z, T423., T4230, T4231, T4232, T4233, T4234, T4235, T4236, T423y, T423z, T42z., T42z0, T42z1, T42z2, T42z3, T42z4, T42z5, T42z6, T42zy, T42zz, T470., T4700, T4701, T4702, T4703, T4704, T4705, T4706, T470y, T470z, T545., T5450, T5451, T5452, T5453, T5454, T5455, T5456, T5457, T5458, T545z, T8..., T80.., T800., T801., T802., T8020, T8021, T8022, T8023, T802z, T803., T8030, T803z, T804., T8040, T8041, T8042, T804z, T805., T8050, T8051, T805z, T806., T8060, T8061, T8062, T8063, T8064, T806z, T807., T8070, T807z, T80y., T80y0, T80yz, T80z., T81.., T810., T811., T812., T813., T814., T815., T81z., T82.., T820., T821., T822., T8220, T8221, T8222, T822z, T823., T824., T825., T82y., T82z., T83.., T830., T8300, T8301, T8302, T8303, T830z, T831., T8310, T8311, T8312, T831z, T832., T8320, T8321, T8322, T8323, T8324, T8325, T832z, T83y., T83y0, T83y1, T83yz, T83z., T84.., T840., T8400, T8401, T8402, T840z, T841., T8410, T8411, T8412, T8413, T8414, T8415, T841z, T842., T8420, T8421, T842z, T843., T8430, T8431, T843z, T84z., T85.., T850., T8500, T8501, T8502, T8503, T8504, T850z, T851., T8510, T8511, T8512, T8513, T8514, T851z, T852., T8520, T8521, T8522, T8523, T852z, T853., T8530, T8531, T8532, T853z, T854., T8540, T8541, T8542, T8543, T854z, T855., T8550, T8551, T855z, T856., T8560, T8561, T856z, T85y., T85z., T86.., T87.., T88.., T880., T881., T882., T883., T884., T885., T886., T887., T8870, T8871, T8872, T8873, T8874, T887z, T88y., T88y0, T88yz, T88z., T8z.., T9..., T90.., T900., T901., T9010, T9011, T9012, T9013, T901z, T902., T9020, T9021, T902z, T903., T9030, T9031, T9032, T9033, T903z, T904., T90y., T90z., T91.., T910., T911., T912., T913., T9130, T913z, T914., T915., T916., T9160, T9161, T9162, T9163, T916z, T91z., T92.., T920., T9200, T9201, T9202, T920z, T921., T9210, T9211, T9212, T9213, T921z, T922., T923., T9230, T923z, T924., T9240, T924z, T92z., T93.., T930., T9300, T9301, T9302, T9303, T9304, T9305, T930z, T931., T9310, T9311, T9312, T9313, T9314, T9315, T9316, T9317, T9318, T931z, T932., T9320, T9321, T9322, T932z, T933., T934., T9340, T934z, T935., T9350, T9351, T9352, T9353, T9354, T9355, T935z, T936., T9360, T9361, T936z, T937., T9370, T9371, T9372, T9373, T9374, T937z, T938., T9380, T9381, T9382, T938z, T93z., T94.., T940., T9400, T940z, T941., T9410, T9411, T9412, T941z, T942., T9420, T9421, T942z, T94y., T94z., T95.., T950., T951., T952., T953., T9530, T9531, T953z, T954., T955., T9550, T955y, T955z, T95y., T95z., T96.., T960., T9600, T9601, T9602, T960z, T961., T9610, T9611, T9612, T961z, T962., T9620, T9621, T9622, T962z, T963., T9630, T9631, T9632, T963z, T964., T9640, T9641, T9642, T9643, T9644, T9645, T9646, T9647, T964z, T965., T9650, T9651, T965z, T966., T9660, T966y, T966z, T967., T96y., T96z., T97.., T970., T971., T972., T973., T97z., T98.., T980., T9801, T9802, T980z, T981., T9810, T9811, T9812, T9813, T9814, T981z, T982., T9820, T9821, T9822, T9823, T982z, T983., T9830, T9831, T9832, T9833, T983z, T98y., T98y0, T98y1, T98y2, T98yz, T98z., T99.., T990., T991., T992., T993., T9930, T9931, T9932, T993z, T99y., T99y0, T99y1, T99yz, T99z., T9z.., TD01., TD010, TD011, TD012, TD013, TD014, TD015, TD016, TD017, TD018, TD019, TD01A, TD01z, TD02., TD020, TD021, TD022, TD023, TD024, TD025, TD026, TD027, TD028, TD029, TD02A, TD02z, TD03., TD030, TD031, TD032, TD033, TD034, TD035, TD036, TD037, TD038, TD039, TD03A, TD03z, TD04., TD040, TD041, TD042, TD043, TD044, TD045, TD046, TD047, TD048, TD049, TD04A, TD04z, TD11., TD110, TD111, TD112, TD113, TD114, TD115, TD116, TD117, TD118, TD119, TD11A, TD11B, TD11z, TD12., TD120, TD121, TD122, TD123, TD124, TD125, TD126, TD127, TD128, TD129, TD12A, TD12B, TD12z, TD13., TD130, TD131, TD132, TD133, TD134, TD135, TD136, TD137, TD138, TD139, TD13A, TD13B, TD13z, TD14., TD140, TD141, TD142, TD143, TD144, TD145, TD146, TD147, TD148, TD149, TD14A, TD14B, TD14z, TE5.., TE50., TE500, TE501, TE502, TE503, TE504, TE505, TE506, TE507, TE508, TE509, TE50A, TE50y, TE50z, TE51., TE510, TE511, TE512, TE51z, TE52., TE53., TE530, TE531, TE532, TE53z, TE54., TE540, TE541, TE54z, TE55., TE550, TE551, TE55z, TE56., TE560, TE561, TE562, TE563, TE564, TE565, TE56y, TE56z, TE57., TE5y., TE5z., TF0.., TF00., TF01., TF010, TF011, TF012, TF01z, TF02., TF020, TF021, TF022, TF023, TF024, TF025, TF026, TF02z, TF03., TF030, TF031, TF032, TF033, TF034, TF035, TF03z, TF04., TF0y., TF0y0, TF0y1, TF0yz, TF0z., TF1.., TF10., TF100, TF101, TF102, TF10z, TF11., TF110, TF111, TF112, TF11z, TF12., TF120, TF121, TF122, TF12z, TF13., TF130, TF131, TF132, TF133, TF13z, TF1z., TF2.., TF20., TF200, TF201, TF20z, TF21., TF22., TF220, TF221, TF222, TF22z, TF2z., TF3.., TF30., TF300, TF301, TF30z, TF31., TF32., TF320, TF321, TF32z, TF33., TF330, TF33z, TF3y., TF3y0, TF3yz, TF3z., TF3z0, TF3z1, TF3z2, TF3zz, TG305, TG30A, TK0.., TK00., TK01., TK010, TK011, TK012, TK013, TK014, TK015, TK01z, TK02., TK03., TK04., TK05., TK06., TK07., TK08., TK0z., TK1.., TK10., TK11., TK1y., TK1z., TK2.., TK20., TK21., TK2y., TK2z., TK3.., TK30., TK31., TK3y., TK3z., TK4.., TL2.., TL20., TL21., TL22., TL2z., TL3.., TL30., TL31., TL32., TL33., TL3z., TL4.., TM2.., TM20., TM21., TM22., TM2z., TM80., TM83., TM84., TN0.., TN00., TN01., TN010, TN011, TN012, TN013, TN014, TN015, TN01z, TN02., TN03., TN04., TN05., TN06., TN07., TN08., TN0z., TN1.., TN10., TN11., TN1y., TN1z., TN2.., TN20., TN21., TN2y., TN2z., TN3.., TN30., TN31., TN3y., TN3z., TN4.., TP52., TP720, TP721, U090., U0900, U0901, U0902, U0903, U0904, U0905, U0906, U0907, U0908, U090z, U092., U0920, U0921, U0922, U0923, U0924, U0925, U0926, U0927, U0928, U092z, U13.., U130., U1300, U1301, U1302, U1303, U1304, U1305, U1306, U1307, U130y, U130z, U131., U1310, U1311, U1312, U1313, U1314, U1315, U1316, U1317, U131y, U131z, U132., U1320, U1321, U1322, U1323, U1324, U1325, U1326, U1327, U132y, U132z, U133., U1330, U1331, U1332, U1333, U1334, U1335, U1336, U1337, U133y, U133z, U134., U1340, U1341, U1342, U1343, U1344, U1345, U1346, U1347, U134y, U134z, U135., U1350, U1351, U1352, U1353, U1354, U1355, U1356, U1357, U135y, U135z, U13y., U13y0, U13y1, U13y2, U13y3, U13y4, U13y5, U13y6, U13y7, U13yy, U13yz, U13z., U13z0, U13z1, U13z2, U13z3, U13z4, U13z5, U13z6, U13z7, U13zy, U13zz, U14.., U140., U1400, U1401, U1402, U1403, U1404, U1405, U1406, U1407, U140y, U140z, U141., U1410, U1412, U1413, U1414, U1415, U1416, U1417, U1418, U141y, U141z, U142., U1420, U1421, U1422, U1423, U1424, U1425, U1426, U1427, U142y, U142z, U143., U1430, U1431, U1432, U1433, U1434, U1435, U1436, U1437, U143y, U143z, U144., U1440, U1441, U1442, U1443, U1444, U1445, U1446, U1447, U144y, U144z, U145., U1450, U1451, U1452, U1453, U1454, U1455, U1456, U1457, U145y, U145z, U146., U1460, U1461, U1462, U1463, U1464, U1465, U1466, U1467, U1468, U146z, U14y., U14y0, U14y1, U14y2, U14y3, U14y4, U14y5, U14y6, U14y7, U14yy, U14yz, U14z., U14z0, U14z1, U14z2, U14z3, U14z4, U14z5, U14z6, U14z7, U14zy, U14zz, U1A.., U1A0., U1A00, U1A01, U1A02, U1A03, U1A04, U1A05, U1A06, U1A07, U1A0y, U1A0z, U1A1., U1A10, U1A11, U1A12, U1A13, U1A14, U1A15, U1A16, U1A17, U1A1y, U1A1z, U1A2., U1A20, U1A21, U1A22, U1A23, U1A24, U1A25, U1A26, U1A27, U1A2y, U1A2z, U1A3., U1A30, U1A31, U1A32, U1A33, U1A34, U1A35, U1A36, U1A37, U1A3y, U1A3z, U1A4., U1A40, U1A41, U1A42, U1A43, U1A44, U1A45, U1A46, U1A47, U1A4y, U1A4z, U1A5., U1A50, U1A51, U1A52, U1A53, U1A54, U1A55, U1A56, U1A57, U1A5y, U1A5z, U1A6., U1A60, U1A61, U1A62, U1A63, U1A64, U1A65, U1A66, U1A67, U1A6y, U1A6z, U1A7., U1A70, U1A71, U1A72, U1A73, U1A74, U1A75, U1A76, U1A77, U1A7y, U1A7z, U1A8., U1A80, U1A81, U1A82, U1A83, U1A84, U1A85, U1A86, U1A87, U1A8y, U1A8z, U1A9., U1A90, U1A91, U1A92, U1A93, U1A94, U1A95, U1A96, U1A97, U1A9y, U1A9z, U1AA., U1AA0, U1AA1, U1AA2, U1AA3, U1AA4, U1AA5, U1AA6, U1AA7, U1AAy, U1AAz, U1AB., U1AB0, U1AB1, U1AB2, U1AB3, U1AB4, U1AB5, U1AB6, U1AB7, U1ABy, U1ABz, U1AC., U1AC0, U1AC1, U1AC2, U1AC3, U1AC4, U1AC5, U1AC6, U1AC7, U1ACy, U1ACz, U1AD., U1AD0, U1AD1, U1AD2, U1AD3, U1AD4, U1AD5, U1AD6, U1AD7, U1ADy, U1ADz, U1Ay., U1Ay0, U1Ay1, U1Ay2, U1Ay3, U1Ay4, U1Ay5, U1Ay6, U1Ay7, U1Ayy, U1Ayz, U20.., U200., U2000, U2001, U2002, U2003, U2004, U2005, U2006, U2007, U200y, U200z, U201., U2010, U2011, U2012, U2013, U2014, U2015, U2016, U2017, U201y, U201z, U202., U2020, U2021, U2022, U2023, U2024, U2025, U2026, U2027, U202y, U202z, U203., U2030, U2031, U2032, U2033, U2034, U2035, U2036, U2037, U203y, U203z, U204., U2040, U2041, U2042, U2043, U2044, U2045, U2046, U2047, U204y, U204z, U205., U2050, U2051, U2052, U2053, U2054, U2055, U2056, U2057, U205y, U205z, U206., U2060, U2061, U2062, U2063, U2064, U2065, U2066, U2067, U206y, U206z, U207., U2070, U2071, U2072, U2073, U2074, U2075, U2076, U2077, U207y, U207z, U208., U2080, U2081, U2082, U2083, U2084, U2085, U2086, U2087, U208y, U208z, U209., U2090, U2091, U2092, U2093, U2094, U2095, U2096, U2097, U209y, U209z, U20A., U20A0, U20A1, U20A2, U20A3, U20A4, U20A5, U20A6, U20A7, U20Ay, U20Az, U20B., U20B0, U20B1, U20B2, U20B3, U20B4, U20B5, U20B6, U20B7, U20By, U20Bz, U20C., U20C0, U20C1, U20C2, U20C3, U20C4, U20C5, U20C6, U20C7, U20Cy, U20Cz, U20y., U20y0, U20y1, U20y2, U20y3, U20y4, U20y5, U20y6, U20y7, U20yy, U20yz, U21.., U210., U211., U212., U213., U214., U215., U216., U217., U21y., U21z., U22.., U220., U221., U222., U223., U224., U225., U226., U227., U22y., U22z., U30.., U300., U301., U302., U303., U304., U305., U306., U307., U30y., U30z., U33.., U330., U331., U332., U333., U334., U335., U336., U337., U33y., U33z., U36.., U360., U361., U362., U363., U364., U365., U366., U367., U36y., U36z., U37.., U370., U371., U372., U373., U374., U375., U376., U377., U37y., U37z., U40.., U400., U4000, U4001, U4002, U4003, U4004, U4005, U4006, U4007, U400y, U400z, U401., U4010, U4011, U4012, U4013, U4014, U4015, U4016, U4017, U401y, U401z, U402., U4020, U4021, U4022, U4023, U4024, U4025, U4026, U4027, U402y, U402z, U403., U4030, U4031, U4032, U4033, U4034, U4035, U4036, U4037, U403y, U403z, U404., U4040, U4041, U4042, U4043, U4044, U4045, U4046, U4047, U404y, U404z, U405., U4050, U4051, U4052, U4053, U4054, U4055, U4056, U4057, U405y, U405z, U406., U4060, U4061, U4062, U4063, U4064, U4065, U4066, U4067, U406y, U406z, U407., U4070, U4071, U4072, U4073, U4074, U4075, U4076, U4077, U407y, U407z, U408., U4080, U4081, U4082, U4083, U4084, U4085, U4086, U4087, U408y, U408z, U409., U4090, U4091, U4092, U4093, U4094, U4095, U4096, U4097, U409y, U409z, U40A., U40A0, U40A1, U40A2, U40A3, U40A4, U40A5, U40A6, U40A7, U40Ay, U40Az, U40B., U40B0, U40B1, U40B2, U40B3, U40B4, U40B5, U40B6, U40B7, U40By, U40Bz, U40C., U40C0, U40C1, U40C2, U40C3, U40C4, U40C5, U40C6, U40C7, U40Cy, U40Cz, U40y., U40y0, U40y1, U40y2, U40y3, U40y4, U40y5, U40y6, U40y7, U40yy, U40yz, U41.., U410., U411., U412., U413., U414., U415., U416., U417., U41y., U41z., U42.., U420., U421., U422., U423., U424., U425., U426., U427., U42y., U42z. |
| 99 Unkown | S...., S3z.., S3z0., S3z00, S3z1., S3z2., S3zz., S4..., S496z, S497., S497z, S49Ez, S49F., S49Fz, S49x., S49y., S49z., S4J.., S4J0., S4J1., S4J2., S4J3., S4z.., S5..., S5E.., S5Ez., S5P.., S5Pz., S5W.., S5y.., S5yy., S5yz., S5yz1, S5z.., S89.., S89x., S89y., S89z., SA8.., SB..., SBz.., SD..., SD90., SD91., SD92., SD920, SD93., SD94., SD95., SD96., SD960, SD97., SD98., SD99., SD9y., SD9z., SDz.., SE..., SE46., SE4z., SEz.., SF..., SF4z., SFz.., SG..., SGz.., SH..., SH6z0, SH6z1, SH6z2, SH8.., SH80., SH800, SH801, SH80z, SH81., SH810, SH811, SH812, SH813, SH814, SH81z, SH82., SH820, SH821, SH822, SH823, SH82z, SH83., SH830, SH831, SH832, SH833, SH834, SH83z, SH84., SH840, SH841, SH842, SH843, SH844, SH845, SH84z, SH85., SH850, SH851, SH852, SH853, SH854, SH855, SH856, SH85z, SH86., SH860, SH861, SH862, SH863, SH864, SH865, SH866, SH867, SH86z, SH87., SH870, SH871, SH872, SH873, SH874, SH875, SH876, SH877, SH878, SH87z, SH88., SH880, SH881, SH882, SH883, SH884, SH885, SH886, SH887, SH888, SH889, SH88z, SH89., SH890, SH891, SH892, SH893, SH894, SH895, SH896, SH897, SH898, SH899, SH89A, SH89z, SH8z., SH9.., SH90., SH91., SH92., SH920, SH921, SH93., SH94., SH95., SH9z., SHz.., SJ..., SJ16., SJ160, SJ161, SJ1y., SJ1y2, SJ1yz, SJ1z., SJ2.., SJ3z., SJ7.., SJz.., SK..., SK0.., SK00., SK01., SK02., SK03., SK04., SK07., SK08., SK09., SK0y., SK0y7, SK0z., SK1.., SK1x., SK1y., SK1z., SKz.., SN48., SN480, SN481, SN482, SN48z, SN552, SN553, SN555, SN560, SN561, SN562, SN563, SN564, Sy..., Syu.., SyuB., SyuB9, SyuBA, SyuBB, SyuBC, SyuBD, SyuBE, SyuBF, SyuBG, SyuBH, SyuC., SyuD., SyuDA, SyuDB, Sz..., T...., T0..., T00.., T000., T0000, T0001, T0002, T0003, T000y, T000z, T001., T0010, T0011, T0012, T0013, T001y, T001z, T00z., T00z0, T00z1, T00z2, T00z3, T00zy, T00zz, T01.., T010., T0100, T0101, T0102, T0103, T010y, T010z, T011., T0110, T0111, T0112, T0113, T011y, T011z, T012., T0120, T0121, T0122, T0123, T012y, T012z, T013., T0130, T0131, T0132, T0133, T013y, T013z, T014., T0140, T0141, T0142, T0143, T014y, T014z, T015., T0150, T0151, T0152, T0153, T015y, T015z, T01x., T01x0, T01x1, T01x2, T01x3, T01xy, T01xz, T01y., T01y0, T01y1, T01y2, T01y3, T01yy, T01yz, T01z., T01z0, T01z1, T01z2, T01z3, T01zy, T01zz, T02.., T020., T0200, T0201, T0202, T0203, T020y, T020z, T021., T0210, T0211, T0212, T0213, T021y, T021z, T02z., T02z0, T02z1, T02z2, T02z3, T02zy, T02zz, T03.., T030., T0300, T0301, T0302, T0303, T030y, T030z, T031., T0310, T0311, T0312, T0313, T031y, T031z, T032., T0320, T0321, T0322, T0323, T032y, T032z, T03z., T03z0, T03z1, T03z2, T03z3, T03zy, T03zz, T04.., T040., T0400, T0401, T040y, T040z, T041., T0410, T0411, T0412, T0413, T041y, T041z, T042., T0420, T0421, T042y, T042z, T04z., T04z0, T04z1, T04z2, T04z3, T04zy, T04zz, T05.., T050., T0500, T0501, T0502, T0503, T050y, T050z, T051., T0510, T0511, T0512, T0513, T051y, T051z, T052., T0520, T0521, T0522, T0523, T052y, T052z, T053., T0530, T0531, T0532, T0533, T053y, T053z, T05z., T05z0, T05z1, T05z2, T05z3, T05zy, T05zz, T0x.., T0x0., T0x00, T0x01, T0x0y, T0x0z, T0x1., T0x10, T0x11, T0x12, T0x13, T0x1y, T0x1z, T0x2., T0x20, T0x21, T0x22, T0x23, T0x2y, T0x2z, T0x3., T0x30, T0x31, T0x32, T0x33, T0x3y, T0x3z, T0xz., T0xz0, T0xz1, T0xz2, T0xz3, T0xzy, T0xzz, T0y.., T0y0., T0y00, T0y01, T0y02, T0y03, T0y0y, T0y0z, T0y1., T0y10, T0y11, T0y12, T0y13, T0y1y, T0y1z, T0yz., T0yz0, T0yz1, T0yz2, T0yz3, T0yzy, T0yzz, T0z.., T1..., T10.., T100., T1000, T1001, T1002, T1003, T1004, T1005, T1006, T1007, T100y, T100z, T10z., T10z0, T10z1, T10z2, T10z3, T10z4, T10z5, T10z6, T10z7, T10zy, T10zz, T11.., T110., T1100, T1101, T1102, T1103, T1104, T1105, T1106, T1107, T110y, T110z, T11z., T11z0, T11z1, T11z2, T11z3, T11z4, T11z5, T11z6, T11z7, T11zy, T11zz, T12.., T120., T1200, T1201, T1202, T1203, T1204, T1205, T1206, T1207, T120y, T120z, T121., T1210, T1211, T1212, T1213, T1214, T1215, T1216, T1217, T121y, T121z, T122., T1220, T1221, T1222, T1223, T1224, T1225, T1226, T1227, T122y, T122z, T123., T1230, T1231, T1232, T1233, T1234, T1235, T1236, T1237, T123y, T123z, T124., T1240, T1241, T1242, T1243, T1244, T1245, T1246, T1247, T124y, T124z, T12z., T12z0, T12z1, T12z2, T12z3, T12z4, T12z5, T12z6, T12z7, T12zy, T12zz, T13.., T130., T1300, T1301, T1302, T1303, T1304, T1305, T1306, T1307, T130y, T130z, T131., T1310, T1311, T1312, T1313, T1314, T1315, T1316, T1317, T131y, T131z, T132., T1320, T1321, T1322, T1323, T1324, T1325, T1326, T1327, T132y, T132z, T133., T1330, T1331, T1332, T1333, T1334, T1335, T1336, T1337, T133y, T133z, T13z., T13z0, T13z1, T13z2, T13z3, T13z4, T13z5, T13z6, T13z7, T13zy, T13zz, T14.., T140., T1400, T1401, T1402, T1403, T1404, T1405, T1406, T1407, T140y, T140z, T141., T1410, T1411, T1412, T1413, T1414, T1415, T1416, T1417, T141y, T141z, T142., T1420, T1421, T1422, T1423, T1424, T1425, T1426, T1427, T142y, T142z, T14z., T14z0, T14z1, T14z2, T14z3, T14z4, T14z5, T14z6, T14z7, T14zy, T14zz, T15.., T150., T1500, T1501, T1502, T1503, T1504, T1505, T1506, T1507, T150y, T150z, T151., T1510, T1511, T1512, T1513, T1514, T1515, T1516, T1517, T151y, T151z, T152., T1520, T1521, T1522, T1523, T1524, T1525, T1526, T1527, T152y, T152z, T153., T1530, T1531, T1532, T1533, T1534, T1535, T1536, T1537, T153y, T153z, T154., T1540, T1541, T1542, T1543, T1544, T1545, T1546, T1547, T154y, T154z, T155., T1550, T1551, T1552, T1553, T1554, T1555, T1556, T1557, T155y, T155z, T156., T1560, T1561, T1562, T1563, T1564, T1565, T1566, T1567, T156y, T156z, T15z., T15z0, T15z1, T15z2, T15z3, T15z4, T15z5, T15z6, T15z7, T15zy, T15zz, T16.., T160., T1600, T1601, T1602, T1603, T1604, T1605, T1606, T1607, T160y, T160z, T161., T1610, T1611, T1612, T1613, T1614, T1615, T1616, T1617, T161y, T161z, T162., T1620, T1621, T1622, T1623, T1624, T1625, T1626, T1627, T162y, T162z, T163., T1630, T1631, T1632, T1633, T1634, T1635, T1636, T1637, T163y, T163z, T164., T1640, T1641, T1642, T1643, T1644, T1645, T1646, T1647, T164y, T164z, T165., T1650, T1651, T1652, T1653, T1654, T1655, T1656, T1657, T165y, T165z, T166., T1660, T1661, T1662, T1663, T1664, T1665, T1666, T1667, T166y, T166z, T16z., T16z0, T16z1, T16z2, T16z3, T16z4, T16z5, T16z6, T16z7, T16zy, T16zz, T17.., T170., T1700, T1701, T1702, T1703, T1704, T1705, T1706, T1707, T170y, T170z, T171., T1710, T1711, T1712, T1713, T1714, T1715, T1716, T1717, T171y, T171z, T172., T1720, T1721, T1722, T1723, T1724, T1725, T1726, T1727, T172y, T172z, T173., T1730, T1731, T1732, T1733, T1734, T1735, T1736, T1737, T173y, T173z, T17y., T17y0, T17y1, T17y2, T17y3, T17y4, T17y5, T17y6, T17y7, T17yy, T17yz, T17z., T17z0, T17z1, T17z2, T17z3, T17z4, T17z5, T17z6, T17z7, T17zy, T17zz, T18.., T181., T1810, T1811, T1812, T1813, T1814, T1815, T1816, T1817, T181y, T181z, T182., T1820, T1821, T1822, T1823, T1824, T1825, T1826, T1827, T182y, T182z, T183., T1830, T1831, T1832, T1833, T1834, T1835, T1836, T1837, T183y, T183z, T184., T1840, T1841, T1842, T1843, T1844, T1845, T1846, T1847, T184y, T184z, T185., T1850, T1851, T1852, T1853, T1854, T1855, T1856, T1857, T185y, T185z, T186., T1860, T1861, T1862, T1863, T1864, T1865, T1866, T1867, T186y, T186z, T187., T1870, T1871, T1872, T1873, T1874, T1875, T1876, T1877, T187y, T187z, T188., T1880, T1881, T1882, T1883, T1884, T1885, T1886, T1887, T188y, T188z, T189., T1890, T1891, T1892, T1893, T1894, T1895, T1896, T1897, T189y, T189z, T18z., T18z0, T18z1, T18z2, T18z3, T18z4, T18z5, T18z6, T18z7, T18zy, T18zz, T19.., T19z., T19z0, T19z1, T19z2, T19z3, T19z4, T19z5, T19z6, T19z7, T19zy, T19zz, T1z.., T2..., T20.., T200., T2000, T2001, T2002, T2003, T2004, T2005, T2006, T2007, T200y, T200z, T201., T2010, T2011, T2012, T2013, T2014, T2015, T2016, T2017, T201y, T201z, T202., T2020, T2021, T2022, T2023, T2024, T2025, T2026, T2027, T202y, T202z, T203., T2030, T2031, T2032, T2033, T2034, T2035, T2036, T2037, T203y, T203z, T204., T2040, T2041, T2042, T2043, T2044, T2045, T2046, T2047, T204y, T204z, T205., T2050, T2051, T2052, T2053, T2054, T2055, T2056, T2057, T205y, T205z, T20z., T20z0, T20z1, T20z2, T20z3, T20z4, T20z5, T20z6, T20z7, T20zy, T20zz, T21.., T210., T2100, T2101, T2102, T2103, T2104, T2105, T2106, T2107, T210y, T210z, T211., T2110, T2111, T2112, T2113, T2114, T2115, T2116, T2117, T211y, T211z, T212., T2120, T2121, T2122, T2123, T2124, T2125, T2126, T2127, T212y, T212z, T213., T2130, T2131, T2132, T2133, T2134, T2135, T2136, T2137, T213y, T213z, T214., T2140, T2141, T2142, T2143, T2144, T2145, T2146, T2147, T214y, T214z, T215., T2150, T2151, T2152, T2153, T2154, T2155, T2156, T2157, T215y, T215z, T216., T2160, T2161, T2162, T2163, T2164, T2165, T2166, T2167, T216y, T216z, T217., T2170, T2171, T2172, T2173, T2174, T2175, T2176, T2177, T217y, T217z, T218., T2180, T2181, T2182, T2183, T2184, T2185, T2186, T2187, T218y, T218z, T21z., T21z0, T21z1, T21z2, T21z3, T21z4, T21z5, T21z6, T21z7, T21zy, T21zz, T22.., T220., T2200, T2201, T2202, T2203, T2204, T2205, T2206, T2207, T220y, T220z, T221., T2210, T2211, T2212, T2213, T2214, T2215, T2216, T2217, T221y, T221z, T222., T2220, T2221, T2222, T2223, T2224, T2225, T2226, T2227, T222y, T222z, T223., T2230, T2231, T2232, T2233, T2234, T2235, T2236, T2237, T223y, T223z, T224., T2240, T2241, T2242, T2243, T2244, T2245, T2246, T2247, T224y, T224z, T22z., T22z0, T22z1, T22z2, T22z3, T22z4, T22z5, T22z6, T22z7, T22zy, T22zz, T23.., T23z., T23z0, T23z1, T23z2, T23z3, T23z4, T23z5, T23z6, T23z7, T23zy, T23zz, T24.., T240., T2400, T2401, T2402, T2403, T2404, T2405, T2406, T2407, T240y, T240z, T241., T2410, T2411, T2412, T2413, T2414, T2415, T2416, T2417, T241y, T241z, T242., T2420, T2421, T2422, T2423, T2424, T2425, T2426, T2427, T242y, T242z, T24z., T24z0, T24z1, T24z2, T24z3, T24z4, T24z5, T24z6, T24z7, T24zy, T24zz, T25.., T251., T2510, T2511, T2512, T2513, T2514, T2515, T2516, T2517, T251y, T251z, T252., T2520, T2521, T2522, T2523, T2524, T2525, T2526, T2527, T252y, T252z, T253., T2530, T2531, T2532, T2533, T2534, T2535, T2536, T2537, T253y, T253z, T254., T2540, T2541, T2542, T2543, T2544, T2545, T2546, T2547, T254y, T254z, T255., T2550, T2551, T2552, T2553, T2554, T2555, T2556, T2557, T255y, T255z, T256., T2560, T2561, T2562, T2563, T2564, T2565, T2566, T2567, T256y, T256z, T257., T2570, T2571, T2572, T2573, T2574, T2575, T2576, T2577, T257y, T257z, T258., T2580, T2581, T2582, T2583, T2584, T2585, T2586, T2587, T258y, T258z, T259., T2590, T2591, T2592, T2593, T2594, T2595, T2596, T2597, T259y, T259z, T25A., T25A0, T25A1, T25A2, T25A3, T25A4, T25A5, T25A6, T25A7, T25Ay, T25Az, T25B., T25B0, T25B1, T25B2, T25B3, T25B4, T25B5, T25B6, T25B7, T25By, T25Bz, T25z., T25z0, T25z1, T25z2, T25z3, T25z4, T25z5, T25z6, T25z7, T25zy, T25zz, T2z.., T3..., T30.., T300., T3000, T3001, T3002, T3003, T3004, T300y, T300z, T301., T3010, T3011, T3012, T3013, T3014, T301y, T301z, T302., T3020, T3021, T3022, T3023, T3024, T302y, T302z, T303., T3030, T3031, T3032, T3033, T3034, T303y, T303z, T304., T3040, T3041, T3042, T3043, T3044, T304y, T304z, T305., T3050, T3051, T3052, T3053, T3054, T305y, T305z, T306., T3060, T3061, T3062, T3063, T3064, T306y, T306z, T307., T3070, T3071, T3072, T3073, T3074, T307y, T307z, T308., T3080, T3081, T3082, T3083, T3084, T308y, T308z, T30z., T30z0, T30z1, T30z2, T30z3, T30z4, T30zy, T30zz, T31.., T310., T3100, T3101, T3102, T3103, T310y, T310z, T311., T3110, T3111, T3112, T3113, T311y, T311z, T312., T3120, T3121, T3122, T3123, T312y, T312z, T313., T3130, T3131, T3132, T3133, T313y, T313z, T314., T3140, T3141, T3142, T3143, T314y, T314z, T315., T3150, T3151, T3152, T3153, T315y, T315z, T316., T3160, T3161, T3162, T3163, T316y, T316z, T317., T3170, T3171, T3172, T3173, T317y, T317z, T318., T3180, T3181, T3182, T3183, T318y, T318z, T319., T3190, T3191, T3192, T3193, T319y, T319z, T31z., T31z0, T31z1, T31z2, T31z3, T31zy, T31zz, T32.., T320., T3200, T3201, T3202, T320y, T320z, T321., T3210, T3211, T3212, T321y, T321z, T322., T3220, T3221, T3222, T322y, T322z, T323., T3230, T3231, T3232, T323y, T323z, T324., T3240, T3241, T3242, T324y, T324z, T325., T3250, T3251, T3252, T325y, T325z, T326., T3260, T3261, T3262, T326y, T326z, T327., T3270, T3271, T3272, T327y, T327z, T328., T3280, T3281, T3282, T328y, T328z, T32z., T32z0, T32z1, T32z2, T32zy, T32zz, T33.., T330., T3300, T3301, T330y, T330z, T331., T3310, T3311, T331y, T331z, T332., T3320, T3321, T332y, T332z, T333., T3330, T3331, T333y, T333z, T334., T3340, T3341, T334y, T334z, T335., T3350, T3351, T335y, T335z, T336., T3360, T3361, T336y, T336z, T337., T3370, T3371, T337y, T337z, T338., T3380, T3381, T338y, T338z, T339., T3390, T3391, T339y, T339z, T33A., T33A0, T33A1, T33Ay, T33Az, T33B., T33B0, T33B1, T33By, T33Bz, T33C., T33C0, T33C1, T33Cy, T33Cz, T33x., T33x0, T33x1, T33xy, T33xz, T33y., T33y0, T33y1, T33yy, T33yz, T33z., T33z0, T33z1, T33zy, T33zz, T3z.., T4..., T41.., T410., T4100, T4101, T4102, T4103, T4104, T4105, T4106, T410y, T410z, T411., T4110, T4111, T4112, T4113, T4114, T4115, T4116, T411y, T411z, T412., T4120, T4121, T4122, T4123, T4124, T4125, T4126, T412y, T412z, T413., T4130, T4131, T4132, T4133, T4134, T4135, T4136, T413y, T413z, T414., T4140, T4141, T4142, T4143, T4144, T4145, T4146, T414y, T414z, T415., T4150, T4151, T4152, T4153, T4154, T4155, T4156, T415y, T415z, T416., T4160, T4161, T4162, T4163, T4164, T4165, T4166, T416y, T416z, T41z., T41z0, T41z1, T41z2, T41z3, T41z4, T41z5, T41z6, T41zy, T41zz, T43.., T430., T4300, T4301, T4302, T4303, T4304, T4305, T4306, T430y, T430z, T431., T4310, T4311, T4312, T4313, T4314, T4315, T4316, T431y, T431z, T43z., T43z0, T43z1, T43z2, T43z3, T43z4, T43z5, T43z6, T43zy, T43zz, T44.., T440., T4400, T4401, T4402, T4403, T4404, T4405, T4406, T440y, T440z, T44z., T44z0, T44z1, T44z2, T44z3, T44z4, T44z5, T44z6, T44zy, T44zz, T45.., T450., T4500, T4501, T4502, T4503, T4504, T4505, T4506, T450y, T450z, T451., T4510, T4511, T4512, T4513, T4514, T4515, T4516, T451y, T451z, T452., T4520, T4521, T4522, T4523, T4524, T4525, T4526, T452y, T452z, T453., T4530, T4531, T4532, T4533, T4534, T4535, T4536, T453y, T453z, T454., T4540, T4541, T4542, T4543, T4544, T4545, T4546, T454y, T454z, T45z., T45z0, T45z1, T45z2, T45z3, T45z4, T45z5, T45z6, T45zy, T45zz, T46.., T460., T4600, T4601, T4602, T4603, T4604, T4605, T4606, T460y, T460z, T461., T4610, T4611, T4612, T4613, T4614, T4615, T4616, T461y, T461z, T46z., T46z0, T46z1, T46z2, T46z3, T46z4, T46z5, T46z6, T46zy, T46zz, T47.., T471., T4710, T4711, T4712, T4713, T4714, T4715, T4716, T471y, T471z, T472., T4720, T4721, T4722, T4723, T4724, T4725, T4726, T472y, T472z, T473., T4730, T4731, T4732, T4733, T4734, T4735, T4736, T473y, T473z, T474., T4740, T4741, T4742, T4743, T4744, T4745, T4746, T474y, T474z, T475., T4750, T4751, T4752, T4753, T4754, T4755, T4756, T475y, T475z, T476., T4760, T4761, T4762, T4763, T4764, T4765, T4766, T476y, T476z, T477., T4770, T4771, T4772, T4773, T4774, T4775, T4776, T477y, T477z, T47z., T47z0, T47z1, T47z2, T47z3, T47z4, T47z5, T47z6, T47zy, T47zz, T4z.., T5..., T50.., T500., T5000, T5001, T5002, T5003, T5004, T5005, T5006, T5007, T5008, T500z, T501., T5010, T5011, T5012, T5013, T5014, T5015, T5016, T5017, T5018, T501z, T502., T5020, T5021, T5022, T5023, T5024, T5025, T5026, T5027, T5028, T502z, T503., T5030, T5031, T5032, T5033, T5034, T5035, T5036, T5037, T5038, T503z, T504., T5040, T5041, T5042, T5043, T5044, T5045, T5046, T5047, T5048, T504z, T505., T5050, T5051, T5052, T5053, T5054, T5055, T5056, T5057, T5058, T505z, T506., T5060, T5061, T5062, T5063, T5064, T5065, T5066, T5067, T5068, T506z, T507., T5070, T5071, T5072, T5073, T5074, T5075, T5076, T5077, T5078, T507z, T508., T5080, T5081, T5082, T5083, T5084, T5085, T5086, T5087, T5088, T508z, T509., T5090, T5091, T5092, T5093, T5094, T5095, T5096, T5097, T5098, T509z, T50y., T50y0, T50y1, T50y2, T50y3, T50y4, T50y5, T50y6, T50y7, T50y8, T50yz, T50z., T50z0, T50z1, T50z2, T50z3, T50z4, T50z5, T50z6, T50z7, T50z8, T50zz, T51.., T510., T5100, T5101, T5102, T5103, T5104, T5105, T5106, T5107, T5108, T510z, T511., T5110, T5111, T5112, T5113, T5114, T5115, T5116, T5117, T5118, T511z, T512., T5120, T5121, T5122, T5123, T5124, T5125, T5126, T5127, T5128, T512z, T513., T5130, T5131, T5132, T5133, T5134, T5135, T5136, T5137, T5138, T513z, T514., T5140, T5141, T5142, T5143, T5144, T5145, T5146, T5147, T5148, T514z, T51z., T51z0, T51z1, T51z2, T51z3, T51z4, T51z5, T51z6, T51z7, T51z8, T51zz, T52.., T520., T5200, T5201, T5202, T520z, T521., T5210, T5211, T5212, T521z, T522., T5220, T5221, T5222, T522z, T523., T5230, T5231, T5232, T523z, T524., T5240, T5241, T5242, T524z, T52z., T52z0, T52z1, T52z2, T52zz, T53.., T530., T5300, T5301, T5302, T5303, T5304, T5305, T5306, T5307, T5308, T530z, T531., T5310, T5311, T5312, T5313, T5314, T5315, T5316, T5317, T5318, T531z, T532., T5320, T5321, T5322, T5323, T5324, T5325, T5326, T5327, T5328, T532z, T533., T5330, T5331, T5332, T5333, T5334, T5335, T5336, T5337, T5338, T533z, T534., T5340, T5341, T5342, T5343, T5344, T5345, T5346, T5347, T5348, T534z, T53z., T53z0, T53z1, T53z2, T53z3, T53z4, T53z5, T53z6, T53z7, T53z8, T53zz, T54.., T540., T5400, T5401, T5402, T5403, T5404, T5405, T5406, T5407, T5408, T540z, T541., T5410, T5411, T5412, T5413, T5414, T5415, T5416, T5417, T5418, T541z, T542., T5420, T5421, T5422, T5423, T5424, T5425, T5426, T5427, T5428, T542z, T543., T5430, T5431, T5432, T5433, T5434, T5435, T5436, T5437, T5438, T543z, T544., T5440, T5441, T5442, T5443, T5444, T5445, T5446, T5447, T5448, T544z, T546., T5460, T5461, T5462, T5463, T5464, T5465, T5466, T5467, T5468, T546z, T54z., T54z0, T54z1, T54z2, T54z3, T54z4, T54z5, T54z6, T54z7, T54z8, T54zz, T55.., T550., T5500, T5501, T550z, T55z., T55z0, T55z1, T55zz, T5z.., T6..., T60.., T600., T601., T602., T603., T604., T605., T606., T607., T608., T609., T60A., T60B., T60C., T60D., T60E., T60F., T60G., T60z., T61.., T610., T611., T612., T613., T614., T615., T616., T61z., T62.., T620., T621., T62z., T6z.., T7..., T70.., T700., T701., T702., T703., T704., T705., T706., T707., T708., T709., T70A., T70B., T70C., T70z., T71.., T710., T711., T71z., T72.., T720., T721., T722., T72z., T73.., T730., T731., T732., T733., T734., T735., T736., T737., T738., T739., T73A., T73B., T73C., T73D., T73z., T74.., T740., T741., T742., T743., T744., T745., T746., T747., T748., T749., T74A., T74B., T74C., T74D., T74E., T74F., T74G., T74H., T74J., T74K., T74L., T74M., T74N., T74P., T74Q., T74R., T74S., T74T., T74U., T74z., T75.., T750., T751., T75z., T76.., T760., T761., T762., T763., T764., T765., T766., T767., T768., T769., T76A., T76B., T76C., T76D., T76E., T76F., T76G., T76H., T76J., T76K., T76L., T76M., T76N., T76P., T76Q., T76R., T76S., T76T., T76U., T76V., T76z., T77.., T770., T771., T772., T773., T774., T775., T776., T777., T77z., T7y.., T7y0., T7y1., T7y2., T7y3., T7y4., T7y5., T7y6., T7y7., T7y8., T7y9., T7yA., T7yB., T7yC., T7yD., T7yE., T7yF., T7yG., T7yH., T7yJ., T7yK., T7yL., T7yM., T7yN., T7yP., T7yQ., T7yz., T7z.., TC..., TC0.., TC00., TC000, TC001, TC00z, TC01., TC010, TC011, TC01z, TC02., TC020, TC021, TC02z, TC0z., TC1.., TC10., TC11., TC1z., TC2.., TC20., TC21., TC22., TC23., TC24., TC25., TC26., TC27., TC28., TC29., TC2z., TC3.., TC30., TC300, TC301, TC302, TC303, TC304, TC305, TC30z, TC31., TC32., TC320, TC321, TC32z, TC3y., TC3y0, TC3y1, TC3y2, TC3y3, TC3y4, TC3y5, TC3y6, TC3yz, TC3z., TC4.., TC40., TC41., TC42., TC420, TC421, TC42z, TC4y., TC4y0, TC4y1, TC4y2, TC4y3, TC4yz, TC4z., TC5.., TC50., TC51., TC52., TC53., TC5z., TC6.., TC60., TC600, TC60y, TC60z, TC6y., TC6y0, TC6y1, TC6y2, TC6yz, TC6z., TC7.., TCy.., TCy0., TCyz., TCz.., TD..., TD0.., TD00., TD000, TD001, TD002, TD003, TD004, TD005, TD006, TD007, TD008, TD009, TD00A, TD00z, TD05., TD050, TD051, TD052, TD053, TD054, TD055, TD056, TD057, TD058, TD059, TD05A, TD05z, TD06., TD060, TD061, TD062, TD063, TD064, TD065, TD066, TD067, TD068, TD069, TD06A, TD06z, TD07., TD070, TD071, TD072, TD073, TD074, TD075, TD076, TD077, TD078, TD079, TD07A, TD07z, TD08., TD080, TD081, TD082, TD083, TD084, TD085, TD086, TD087, TD088, TD089, TD08A, TD08z, TD09., TD090, TD091, TD092, TD093, TD094, TD095, TD096, TD097, TD098, TD099, TD09A, TD09z, TD0y., TD0y0, TD0y1, TD0y2, TD0y3, TD0y4, TD0y5, TD0y6, TD0y7, TD0y8, TD0y9, TD0yA, TD0yz, TD0z., TD1.., TD10., TD100, TD101, TD102, TD103, TD104, TD105, TD106, TD107, TD108, TD109, TD10A, TD10B, TD10z, TD15., TD150, TD151, TD152, TD153, TD154, TD155, TD156, TD157, TD158, TD159, TD15A, TD15B, TD15z, TD16., TD160, TD161, TD162, TD163, TD164, TD165, TD166, TD167, TD168, TD169, TD16A, TD16B, TD16z, TD17., TD170, TD171, TD172, TD173, TD174, TD175, TD176, TD177, TD178, TD179, TD17A, TD17B, TD17z, TD18., TD180, TD181, TD182, TD183, TD184, TD185, TD186, TD187, TD188, TD189, TD18A, TD18B, TD18z, TD19., TD190, TD191, TD192, TD193, TD194, TD195, TD196, TD197, TD198, TD199, TD19A, TD19B, TD19z, TD1y., TD1y0, TD1y1, TD1y2, TD1y3, TD1y4, TD1y5, TD1y6, TD1y7, TD1y8, TD1y9, TD1yA, TD1yB, TD1yz, TD1z., TD2.., TD20., TD21., TD22., TD23., TD24., TD25., TD26., TD27., TD2z., TD3.., TD30., TD300, TD301, TD302, TD303, TD304, TD305, TD306, TD307, TD30z, TD31., TD310, TD311, TD312, TD313, TD314, TD315, TD316, TD317, TD31z, TD32., TD320, TD321, TD322, TD32z, TD3y., TD3y0, TD3y1, TD3y2, TD3y3, TD3y4, TD3y5, TD3y6, TD3y7, TD3y8, TD3y9, TD3yz, TD3z., TD4.., TD40., TD41., TD42., TD43., TD44., TD45., TD46., TD4z., TD5.., TD50., TD51., TD52., TD53., TD54., TD55., TD56., TD57., TD570, TD571, TD572, TD573, TD5z., TD6.., TD60., TD61., TD62., TD63., TD64., TD65., TD66., TD67., TD6z., TD7.., TD70., TD71., TD72., TD7z., TDy.., TDy0., TDyy., TDyy0, TDyy1, TDyy2, TDyy3, TDyy4, TDyy5, TDyy6, TDyy7, TDyy8, TDyy9, TDyyA, TDyyz, TDyz., TDz.., TDz1., TE6.., TE60., TE61., TE62., TE63., TE630, TE631, TE632, TE633, TE63z, TE64., TE640, TE64z, TE6y., TE6y0, TE6y1, TE6y2, TE6y3, TE6y4, TE6y5, TE6y6, TE6y7, TE6y8, TE6yz, TE6z., TE7.., TE8.., TE80., TE81., TE82., TE83., TE84., TE85., TE86., TE87., TE88., TE89., TE8A., TE8z., TE9.., TE90., TE91., TE92., TE93., TE94., TE95., TE96., TE97., TE9z., TEz.., TF..., TF5.., TFz.., TG..., TG0.., TG00., TG01., TG02., TG03., TG04., TG05., TG06., TG0z., TG1.., TG10., TG100, TG101, TG102, TG103, TG104, TG105, TG106, TG107, TG10y, TG10z, TG11., TG110, TG111, TG112, TG11z, TG12., TG13., TG130, TG131, TG132, TG133, TG134, TG13z, TG14., TG140, TG141, TG142, TG143, TG144, TG14z, TG15., TG150, TG151, TG152, TG153, TG154, TG15z, TG16., TG160, TG161, TG162, TG163, TG164, TG16z, TG17., TG170, TG171, TG172, TG173, TG174, TG17z, TG1z., TG1z0, TG1zz, TG2.., TG20., TG21., TG22., TG23., TG24., TG25., TG26., TG2z., TG3.., TG30., TG300, TG301, TG302, TG303, TG304, TG306, TG307, TG308, TG309, TG30B, TG30C, TG30D, TG30E, TG30F, TG30G, TG30H, TG30z, TG31., TG310, TG311, TG312, TG313, TG314, TG315, TG316, TG317, TG31z, TG32., TG320, TG321, TG322, TG323, TG324, TG32z, TG33., TG330, TG331, TG332, TG333, TG334, TG335, TG336, TG337, TG338, TG33z, TG34., TG340, TG341, TG342, TG343, TG344, TG345, TG346, TG347, TG348, TG34z, TG35., TG350, TG351, TG352, TG353, TG354, TG355, TG356, TG357, TG35z, TG36., TG360, TG361, TG362, TG363, TG36z, TG37., TG370, TG371, TG372, TG373, TG374, TG375, TG376, TG37z, TG38., TG380, TG381, TG382, TG3y., TG3y0, TG3y1, TG3y2, TG3y3, TG3y4, TG3y5, TG3y6, TG3y7, TG3yz, TG3z., TG4.., TG40., TG41., TG410, TG411, TG412, TG413, TG414, TG415, TG41z, TG42., TG420, TG421, TG422, TG423, TG424, TG425, TG42z, TG43., TG430, TG431, TG432, TG43z, TG44., TG440, TG441, TG442, TG443, TG444, TG445, TG446, TG447, TG448, TG449, TG44A, TG44B, TG44C, TG44D, TG44E, TG44z, TG4y., TG4y0, TG4y1, TG4y2, TG4y3, TG4y4, TG4y5, TG4y6, TG4y7, TG4y8, TG4yz, TG4z., TG5.., TG50., TG51., TG510, TG511, TG51z, TG5y., TG5y0, TG5y1, TG5y2, TG5yz, TG5z., TG6.., TG60., TG600, TG601, TG60z, TG61., TG62., TG63., TG630, TG631, TG63z, TG6y., TG6y0, TG6yz, TG6z., TG6z0, TG6z1, TG6zz, TG7.., TG70., TG71., TG710, TG711, TG712, TG713, TG71z, TG72., TG720, TG721, TG722, TG723, TG724, TG725, TG726, TG72y, TG72z, TG7y., TG7y0, TG7y1, TG7y2, TG7y3, TG7y4, TG7y5, TG7y6, TG7y7, TG7yz, TG7z., TG8.., TG80., TG800, TG801, TG802, TG803, TG804, TG805, TG806, TG807, TG808, TG809, TG80A, TG80B, TG80C, TG80D, TG80y, TG80z, TG81., TG810, TG811, TG812, TG813, TG814, TG815, TG81y, TG81z, TG8y., TG8y0, TG8y1, TG8y2, TG8y3, TG8y4, TG8yz, TG8z., TG9.., TG90., TG91., TG910, TG911, TG912, TG91z, TG92., TG920, TG921, TG922, TG923, TG92z, TG9y., TG9y0, TG9y1, TG9y2, TG9y3, TG9y4, TG9yz, TG9z., TG9z0, TG9z1, TG9z2, TG9zz, TGyy., TGyz., TGyz0, TGyz1, TGyz3, TGyz4, TGyz5, TGyz6, TGyz7, TGyzz, TGz.., TK..., TK5.., TK50., TK51., TK52., TK53., TK54., TK55., TK5z., TK6.., TK60., TK61., TK6z., TK7.., TK70., TK71., TK72., TK7z., TKx.., TKx0., TKx00, TKx01, TKx0z, TKx1., TKx2., TKx3., TKx4., TKx5., TKx6., TKx7., TKxy., TKxz., TKz.., TL..., TL0.., TL00., TL0z., TL1.., TL10., TL11., TL1z., TL5.., TL50., TL500, TL501, TL50z, TL51., TL52., TL53., TL54., TL55., TL56., TL57., TL5x., TL5y., TL5y0, TL5y1, TL5y2, TL5yy, TL5yz, TL5z., TL6.., TL60., TL61., TL62., TL63., TL64., TL6z., TL7.., TL70., TL7y., TL7z., TL9.., TLx.., TLx0., TLx00, TLx0z, TLx1., TLx2., TLx20, TLx21, TLx2z, TLx3., TLxy., TLxy0, TLxyz, TLxz., TLxz0, TLxz1, TLxz2, TLxz3, TLxz4, TLxzz, TLz.., TM..., TM0.., TM00., TM01., TM02., TM03., TM0z., TM1.., TM10., TM11., TM12., TM13., TM1z., TM3.., TM30., TM31., TM3z., TM4.., TM40., TM4x., TM4y., TM4z., TM5.., TM50., TM51., TM5z., TM6.., TM8.., TM82., TM85., TM8z., TMz.., TN..., TN5.., TN50., TN51., TN52., TN53., TN54., TN55., TN5z., TN6.., TN60., TN61., TN6z., TN7.., TN70., TN71., TN72., TN7z., TN8.., TN80., TN800, TN801, TN80z, TN81., TN82., TN84., TN85., TN86., TN87., TN8y., TN8z., TNz.., TP..., TP0.., TP00., TP0z., TP1.., TP10., TP11., TP12., TP120, TP121, TP122, TP123, TP124, TP12z, TP13., TP1z., TP1z0, TP1z1, TP1z2, TP1z3, TP1z4, TP1z5, TP1z6, TP1z7, TP1zz, TP2.., TP20., TP21., TP22., TP23., TP24., TP2z., TP3.., TP30., TP31., TP32., TP33., TP34., TP35., TP36., TP3z., TP4.., TP40., TP41., TP42., TP43., TP4z., TP5.., TP50., TP51., TP5z., TP6.., TP60., TP61., TP62., TP63., TP6z., TP7.., TP70., TP71., TP72., TP722, TP72z, TP7y., TP7z., TP8.., TPz.., Tz..., U0..., U00.., U000., U0000, U0001, U000z, U001., U0010, U0011, U001z, U002., U0020, U0021, U002z, U003., U0030, U0031, U003z, U004., U0040, U0041, U004z, U005., U0050, U0051, U005z, U00z., U00z0, U00z1, U00z2, U00z3, U00zz, U01.., U010., U0100, U0101, U0102, U0103, U0104, U0105, U010z, U011., U0110, U0111, U0112, U0113, U0114, U0115, U011z, U012., U0120, U0121, U0122, U0123, U0124, U0125, U012z, U013., U0130, U0131, U0132, U0133, U0134, U0135, U013z, U014., U0140, U0141, U0142, U0143, U0144, U0145, U014z, U015., U0150, U0151, U0152, U0153, U0154, U0155, U015z, U016., U0160, U0161, U0162, U0163, U0164, U0165, U016z, U017., U0170, U0171, U0172, U0173, U0174, U0175, U017z, U018., U0180, U0181, U0182, U0183, U0184, U0185, U018z, U01z., U01z0, U01z1, U01z2, U01z3, U01z4, U01z5, U01z6, U01z8, U01zz, U02.., U020., U0200, U0201, U0202, U0203, U0204, U0205, U020z, U021., U0210, U0211, U0212, U0213, U0214, U0215, U021z, U022., U0220, U0221, U0222, U0223, U0224, U0225, U022z, U023., U0230, U0231, U0232, U0233, U0234, U0235, U023z, U024., U0240, U0241, U0242, U0243, U0244, U0245, U024z, U025., U0250, U0251, U0252, U0253, U0254, U0255, U025z, U026., U0260, U0261, U0262, U0263, U0264, U0265, U026z, U027., U0270, U0271, U0272, U0273, U0274, U0275, U027z, U028., U0280, U0281, U0282, U0283, U0284, U0285, U028z, U02z., U02z0, U02z1, U02z2, U02z3, U02z4, U02z5, U02z6, U02z8, U02zz, U03.., U030., U0300, U0301, U0302, U0303, U0304, U0305, U0306, U0307, U030z, U031., U0310, U0311, U0312, U0313, U0314, U0315, U0316, U0317, U031z, U032., U0320, U0321, U0322, U0323, U0324, U0325, U0326, U0327, U032z, U033., U0330, U0331, U0332, U0333, U0334, U0335, U0336, U0337, U033z, U034., U0340, U0341, U0342, U0343, U0344, U0345, U0346, U0347, U034z, U035., U0350, U0351, U0352, U0353, U0354, U0355, U0356, U0357, U035z, U036., U0360, U0361, U0362, U0363, U0364, U0365, U0366, U0367, U036z, U037., U0370, U0371, U0372, U0373, U0374, U0375, U0376, U0377, U037z, U038., U0380, U0381, U0382, U0383, U0384, U0385, U0386, U0387, U038z, U03z., U03z0, U03z1, U03z2, U03z3, U03z4, U03z5, U03z6, U03z8, U03zz, U04.., U040., U0400, U0401, U0402, U0403, U0404, U0405, U0406, U0407, U040z, U041., U0410, U0411, U0412, U0413, U0414, U0415, U0416, U0417, U041z, U042., U0420, U0421, U0422, U0423, U0424, U0425, U0426, U0427, U042z, U043., U0430, U0431, U0432, U0433, U0434, U0435, U0436, U0437, U043z, U044., U0440, U0441, U0442, U0443, U0444, U0445, U0446, U0447, U044z, U045., U0450, U0451, U0452, U0453, U0454, U0455, U0456, U0457, U045z, U046., U0460, U0461, U0462, U0463, U0464, U0465, U0466, U0467, U046z, U047., U0470, U0471, U0472, U0473, U0474, U0475, U0476, U0477, U047z, U048., U0480, U0481, U0482, U0483, U0484, U0485, U0486, U0487, U048z, U04z., U04z0, U04z1, U04z2, U04z3, U04z4, U04z5, U04z6, U04z8, U04zz, U05.., U050., U0500, U0501, U0502, U0503, U0504, U0505, U0506, U0507, U050z, U051., U0510, U0511, U0512, U0513, U0514, U0515, U0516, U0517, U051z, U052., U0520, U0521, U0522, U0523, U0524, U0525, U0526, U0527, U052z, U053., U0530, U0531, U0532, U0533, U0534, U0535, U0536, U0537, U053z, U054., U0540, U0541, U0542, U0543, U0544, U0545, U0546, U0547, U054z, U055., U0550, U0551, U0552, U0553, U0554, U0555, U0556, U0557, U055z, U056., U0560, U0561, U0562, U0563, U0564, U0565, U0566, U0567, U056z, U057., U0570, U0571, U0572, U0573, U0574, U0575, U0576, U0577, U057z, U058., U0580, U0581, U0582, U0583, U0584, U0585, U0586, U0587, U058z, U05z., U05z0, U05z1, U05z2, U05z3, U05z4, U05z5, U05z6, U05z8, U05zz, U06.., U060., U0600, U0601, U0602, U0603, U0604, U0605, U0606, U0607, U060z, U061., U0610, U0611, U0612, U0613, U0614, U0615, U0616, U0617, U061z, U062., U0620, U0621, U0622, U0623, U0624, U0625, U0626, U0627, U062z, U063., U0630, U0631, U0632, U0633, U0634, U0635, U0636, U0637, U063z, U064., U0640, U0641, U0642, U0643, U0644, U0645, U0646, U0647, U064z, U065., U0650, U0651, U0652, U0653, U0654, U0655, U0656, U0657, U065z, U066., U0660, U0661, U0662, U0663, U0664, U0665, U0666, U0667, U066z, U067., U0670, U0671, U0672, U0673, U0674, U0675, U0676, U0677, U067z, U068., U0680, U0681, U0682, U0683, U0684, U0685, U0686, U0687, U068z, U06z., U06z0, U06z1, U06z2, U06z3, U06z4, U06z5, U06z6, U06z8, U06zz, U07.., U070., U0700, U0701, U0702, U0703, U0704, U0705, U0706, U0707, U070z, U071., U0710, U0711, U0712, U0713, U0714, U0715, U0716, U0717, U071z, U072., U0720, U0721, U0722, U0723, U0724, U0725, U0726, U0727, U072z, U073., U0730, U0731, U0732, U0733, U0734, U0735, U0736, U0737, U073z, U074., U0740, U0741, U0742, U0743, U0744, U0745, U0746, U0747, U074z, U075., U0750, U0751, U0752, U0753, U0754, U0755, U0756, U0757, U075z, U076., U0760, U0761, U0762, U0763, U0764, U0765, U0766, U0767, U076z, U077., U0770, U0771, U0772, U0773, U0774, U0775, U0776, U0777, U077z, U078., U0780, U0781, U0782, U0783, U0784, U0785, U0786, U0787, U078z, U07z., U07z0, U07z1, U07z2, U07z3, U07z4, U07z5, U07z6, U07z8, U07zz, U08.., U080., U0800, U0801, U0802, U0803, U0804, U0805, U0806, U0807, U0808, U080z, U081., U0810, U0811, U0812, U0813, U0814, U0815, U0816, U0817, U0818, U081z, U082., U0820, U0821, U0822, U0823, U0824, U0825, U0826, U0827, U0828, U082z, U083., U0830, U0831, U0832, U0833, U0834, U0835, U0836, U0837, U083z, U084., U0840, U0841, U0842, U0843, U0844, U0845, U0846, U0847, U084z, U085., U0850, U0851, U0852, U0853, U0854, U0855, U0856, U0857, U085z, U086., U0860, U0861, U0862, U0863, U0864, U0865, U0866, U0867, U086z, U087., U0870, U0871, U0872, U0873, U0874, U0875, U0876, U0877, U0878, U087z, U088., U0880, U0881, U0882, U0883, U0884, U0885, U0886, U0887, U0888, U088z, U08z., U08z0, U08z1, U08z2, U08z3, U08zz, U09.., U091., U0910, U0911, U0912, U0913, U0914, U0915, U0916, U0917, U0918, U091z, U093., U0930, U0931, U0932, U0933, U0934, U0935, U0936, U0937, U0938, U093z, U09z., U09z0, U09z1, U09z2, U09z3, U09z4, U09z5, U09z6, U09z7, U09z8, U09zz, U0A.., U0A0., U0A00, U0A01, U0A02, U0A03, U0A04, U0A0y, U0A0z, U0A1., U0A10, U0A11, U0A12, U0A1y, U0A1z, U0Ay., U0Ay0, U0Ay1, U0Ay2, U0Ay3, U0Ayy, U0z.., U0zy., U0zz., U1..., U10.., U100., U1000, U1001, U1002, U1003, U1004, U1005, U1006, U1007, U100y, U100z, U101., U1010, U1011, U1012, U1013, U1014, U1015, U1016, U1017, U101y, U101z, U102., U1020, U1021, U1022, U1023, U1024, U1025, U1026, U1027, U102y, U102z, U103., U1030, U1031, U1032, U1033, U1034, U1035, U1036, U1037, U103y, U103z, U104., U1040, U1041, U1042, U1043, U1044, U1045, U1046, U1047, U104y, U104z, U105., U1050, U1051, U1052, U1053, U1054, U1055, U1056, U1057, U105y, U105z, U106., U1060, U1061, U1062, U1063, U1064, U1065, U1066, U1067, U106y, U106z, U107., U1070, U1071, U1072, U1073, U1074, U1075, U1076, U1077, U107y, U107z, U108., U1080, U1081, U1082, U1083, U1084, U1085, U1086, U1087, U108y, U108z, U109., U1090, U1091, U1092, U1093, U1094, U1095, U1096, U1097, U109y, U109z, U10A., U10A0, U10A1, U10A2, U10A3, U10A4, U10A5, U10A6, U10A7, U10Ay, U10Az, U10B., U10B0, U10B1, U10B2, U10B3, U10B4, U10B5, U10B6, U10B7, U10By, U10Bz, U10C., U10C0, U10C1, U10C2, U10C3, U10C4, U10C5, U10C6, U10C7, U10Cy, U10Cz, U10D., U10D0, U10D1, U10D2, U10D3, U10D4, U10D5, U10D6, U10D7, U10Dy, U10Dz, U10E., U10E0, U10E1, U10E2, U10E3, U10E4, U10E5, U10E6, U10E7, U10Ey, U10Ez, U10F., U10F0, U10F1, U10F2, U10F3, U10F4, U10F5, U10F6, U10F7, U10Fy, U10Fz, U10G., U10G0, U10G1, U10G2, U10G3, U10G4, U10G5, U10G6, U10G7, U10Gy, U10Gz, U10H., U10H0, U10H1, U10H2, U10H3, U10H4, U10H5, U10H6, U10H7, U10Hy, U10Hz, U10J., U10J0, U10J1, U10J2, U10J3, U10J4, U10J5, U10J6, U10J7, U10Jy, U10Jz, U10z., U10z0, U10z1, U10z2, U10z3, U10z4, U10z5, U10z6, U10z7, U10zy, U10zz, U11.., U110., U1100, U1101, U1102, U1103, U1104, U1105, U1106, U1107, U110y, U110z, U111., U1110, U1111, U1112, U1113, U1114, U1115, U1116, U1117, U111y, U111z, U112., U1120, U1121, U1122, U1123, U1124, U1125, U1126, U1127, U112y, U112z, U113., U1130, U1131, U1132, U1133, U1134, U1135, U1136, U1137, U113y, U113z, U114., U1140, U1141, U1142, U1143, U1144, U1145, U1146, U1147, U114y, U114z, U115., U1150, U1151, U1152, U1153, U1154, U1155, U1156, U1157, U115y, U115z, U116., U1160, U1161, U1162, U1163, U1164, U1165, U1166, U1167, U116y, U116z, U117., U1170, U1171, U1172, U1173, U1174, U1175, U1176, U1177, U1178, U117z, U118., U1180, U1181, U1182, U1183, U1184, U1185, U1186, U1187, U118y, U118z, U119., U1190, U1191, U1192, U1193, U1194, U1195, U1196, U1197, U119y, U119z, U11A., U11A0, U11A1, U11A2, U11A3, U11A4, U11A5, U11A6, U11A7, U11Ay, U11Az, U11B., U11B0, U11B1, U11B2, U11B3, U11B4, U11B5, U11B6, U11B7, U11By, U11Bz, U11C., U11C0, U11C1, U11C2, U11C3, U11C4, U11C5, U11C6, U11C7, U11Cy, U11Cz, U11D., U11D0, U11D1, U11D2, U11D3, U11D4, U11D5, U11D6, U11D7, U11Dy, U11Dz, U11E., U11E0, U11E1, U11E2, U11E3, U11E4, U11E5, U11E6, U11E7, U11E8, U11E9, U11F., U11F0, U11F1, U11F2, U11F3, U11F4, U11F5, U11F6, U11F7, U11Fy, U11Fz, U11G., U11G0, U11G1, U11G2, U11G3, U11G4, U11G5, U11G6, U11G7, U11Gy, U11Gz, U11H., U11H0, U11H1, U11H2, U11H3, U11H4, U11H5, U11H6, U11H7, U11Hy, U11Hz, U11J., U11J0, U11J1, U11J2, U11J3, U11J4, U11J5, U11J6, U11J7, U11Jy, U11Jz, U11K., U11K0, U11K1, U11K2, U11K3, U11K4, U11K5, U11K6, U11K7, U11Ky, U11Kz, U11L., U11L0, U11L1, U11L2, U11L3, U11L4, U11L5, U11L6, U11L7, U11Ly, U11Lz, U11M., U11M0, U11M1, U11M2, U11M3, U11M4, U11M5, U11M6, U11M7, U11My, U11Mz, U11Q., U11Q0, U11Q1, U11Q2, U11Q3, U11Q4, U11Q5, U11Q6, U11Q7, U11Qy, U11Qz, U11R., U11R0, U11R1, U11R2, U11R3, U11R4, U11R5, U11R6, U11R7, U11Ry, U11Rz, U11S., U11S0, U11S1, U11S2, U11S3, U11S4, U11S5, U11S6, U11S7, U11Sy, U11Sz, U11y., U11y0, U11y1, U11y2, U11y3, U11y4, U11y5, U11y6, U11y7, U11yy, U11yz, U12.., U120., U1200, U1201, U1202, U1203, U1204, U1205, U1206, U1207, U120y, U120z, U121., U1210, U1211, U1212, U1213, U1214, U1215, U1216, U1217, U121y, U121z, U122., U1220, U1221, U1222, U1223, U1224, U1225, U1226, U1227, U122y, U122z, U123., U1230, U1231, U1232, U1233, U1234, U1235, U1236, U1237, U123y, U123z, U124., U1240, U1241, U1242, U1243, U1244, U1245, U1246, U1247, U124y, U124z, U125., U1250, U1251, U1252, U1253, U1254, U1255, U1256, U1257, U125y, U125z, U126., U1260, U1261, U1262, U1263, U1264, U1265, U1266, U1267, U126y, U126z, U127., U1270, U1271, U1272, U1273, U1274, U1275, U1276, U1277, U127y, U127z, U128., U1280, U1281, U1282, U1283, U1284, U1285, U1286, U1287, U128y, U128z, U129., U1290, U1291, U1292, U1293, U1294, U1295, U1296, U1297, U129y, U129z, U12A., U12A0, U12A1, U12A2, U12A3, U12A4, U12A5, U12A6, U12A7, U12Ay, U12Az, U12y., U12y0, U12y1, U12y2, U12y3, U12y4, U12y5, U12y6, U12y7, U12yy, U12yz, U15.., U150., U1500, U1501, U1502, U1503, U1504, U1505, U1506, U1507, U150y, U150z, U151., U1510, U1511, U1512, U1513, U1514, U1515, U1516, U1517, U151y, U151z, U152., U1520, U1521, U1522, U1523, U1524, U1525, U1526, U1527, U152y, U152z, U16.., U160., U1600, U1601, U1602, U1603, U1604, U1605, U1606, U1607, U160y, U160z, U161., U1610, U1611, U1612, U1613, U1614, U1615, U1616, U1617, U161y, U161z, U162., U1620, U1621, U1622, U1623, U1624, U1625, U1626, U1627, U162y, U162z, U163., U1630, U1631, U1632, U1633, U1635, U1636, U1637, U1638, U163y, U163z, U164., U1640, U1641, U1642, U1643, U1644, U1645, U1646, U1647, U164y, U164z, U165., U1650, U1651, U1652, U1653, U1654, U1655, U1656, U1657, U165y, U165z, U166., U1660, U1661, U1662, U1663, U1664, U1665, U1666, U1667, U166y, U166z, U16y., U16y0, U16y1, U16y2, U16y3, U16y4, U16y5, U16y6, U16y7, U16yy, U16yz, U16z., U16z0, U16z1, U16z2, U16z3, U16z4, U16z5, U16z6, U16z7, U16zy, U16zz, U17.., U170., U1700, U1701, U1702, U1703, U1704, U1705, U1706, U1707, U170y, U170z, U171., U1710, U1711, U1712, U1713, U1714, U1715, U1716, U1717, U171y, U171z, U172., U1720, U1721, U1722, U1723, U1724, U1725, U1726, U1727, U172y, U172z, U173., U1730, U1731, U1732, U1733, U1734, U1735, U1736, U1737, U173y, U173z, U174., U1740, U1741, U1742, U1743, U1744, U1745, U1746, U1747, U174y, U174z, U175., U1750, U1751, U1752, U1753, U1754, U1755, U1756, U1757, U175y, U175z, U176., U1760, U1761, U1762, U1763, U1764, U1765, U1766, U1767, U176y, U176z, U177., U1770, U1771, U1772, U1773, U1774, U1775, U1776, U1777, U177y, U177z, U178., U1780, U1781, U1782, U1783, U1784, U1785, U1786, U1787, U178y, U178z, U17y., U17y0, U17y1, U17y2, U17y3, U17y4, U17y5, U17y6, U17y7, U17yy, U17yz, U18.., U180., U1800, U1801, U1802, U1803, U1804, U1805, U1806, U1807, U180y, U180z, U181., U1810, U1811, U1812, U1813, U1814, U1815, U1816, U1817, U181y, U181z, U182., U1820, U1821, U1822, U1823, U1824, U1825, U1826, U1827, U182y, U182z, U183., U1830, U1831, U1832, U1833, U1834, U1835, U1836, U1837, U183y, U183z, U184., U1840, U1841, U1842, U1843, U1844, U1845, U1846, U1847, U184y, U184z, U185., U1850, U1851, U1852, U1853, U1854, U1855, U1856, U1857, U185y, U185z, U186., U1860, U1861, U1862, U1863, U1864, U1865, U1866, U1867, U186y, U186z, U187., U1870, U1871, U1872, U1873, U1874, U1875, U1876, U1877, U187y, U187z, U18y., U18y0, U18y1, U18y2, U18y3, U18y4, U18y5, U18y6, U18y7, U18yy, U18yz, U18z., U18z0, U18z1, U18z2, U18z3, U18z4, U18z5, U18z6, U18z7, U18zy, U18zz, U194., U1940, U1941, U1942, U1943, U1944, U1945, U1946, U1947, U194y, U194z, U195., U1950, U1951, U1952, U1953, U1954, U1955, U1956, U1957, U195y, U195z, U196., U1960, U1961, U1962, U1963, U1964, U1965, U1966, U1967, U196y, U196z, U197., U1970, U1971, U1972, U1973, U1974, U1975, U1976, U1977, U197y, U197z, U198., U1980, U1981, U1982, U1983, U1984, U1985, U1986, U1987, U198y, U198z, U1C.., U1Cy., U1Cy0, U1Cy1, U1Cy2, U1Cy3, U1Cy4, U1Cy5, U1Cy6, U1Cy7, U1Cyy, U1Cyz, U1Cz., U1Cz0, U1Cz1, U1Cz2, U1Cz3, U1Cz4, U1Cz5, U1Cz6, U1Cz7, U1Czy, U1Czz, U2..., U23.., U230., U231., U232., U233., U234., U235., U236., U237., U23y., U23z., U24.., U240., U241., U242., U243., U244., U245., U246., U247., U24y., U24z., U25.., U250., U251., U252., U253., U254., U255., U256., U257., U25y., U25z., U26.., U260., U261., U262., U263., U264., U265., U266., U267., U26y., U26z., U27.., U270., U271., U272., U273., U274., U275., U276., U277., U27y., U27z., U28.., U280., U281., U282., U283., U284., U285., U286., U287., U28y., U28z., U29.., U290., U291., U292., U293., U294., U295., U296., U297., U29y., U29z., U2A.., U2A0., U2A1., U2A2., U2A3., U2A4., U2A5., U2A6., U2A7., U2Ay., U2Az., U2B.., U2B0., U2B1., U2B2., U2B3., U2B4., U2B5., U2B6., U2B7., U2By., U2Bz., U2C.., U2C0., U2C1., U2C2., U2C3., U2C4., U2C5., U2C6., U2C7., U2Cy., U2Cz., U2D.., U2D0., U2D1., U2D2., U2D3., U2D4., U2D5., U2D6., U2D7., U2Dy., U2Dz., U2E.., U2y.., U2y0., U2y1., U2y2., U2y3., U2y4., U2y5., U2y6., U2y7., U2yy., U2yz., U2z.., U2z0., U2z1., U2z2., U2z3., U2z4., U2z5., U2z6., U2z7., U2zy., U2zz., U3..., U31.., U310., U311., U312., U313., U314., U315., U316., U317., U31y., U31z., U32.., U320., U321., U322., U323., U324., U325., U326., U327., U32y., U32z., U34.., U340., U341., U342., U343., U344., U345., U346., U347., U34y., U34z., U35.., U350., U351., U352., U353., U354., U355., U356., U357., U35y., U35z., U38.., U380., U381., U382., U383., U384., U385., U386., U387., U38y., U38z., U39.., U390., U391., U392., U393., U394., U395., U396., U397., U39y., U39z., U3A.., U3A0., U3A1., U3A2., U3A3., U3A4., U3A5., U3A6., U3A7., U3Ay., U3Az., U3B.., U3B0., U3B1., U3B2., U3B3., U3B4., U3B5., U3B6., U3B7., U3By., U3Bz., U3C.., U3C0., U3C1., U3C2., U3C3., U3C4., U3C5., U3C6., U3C7., U3Cy., U3Cz., U3D.., U3D0., U3D1., U3D2., U3D3., U3D4., U3D5., U3D6., U3D7., U3Dy., U3Dz., U3E.., U3E0., U3E1., U3E2., U3E3., U3E4., U3E5., U3E6., U3E7., U3Ey., U3Ez., U3F.., U3F0., U3F1., U3F2., U3F3., U3F4., U3F5., U3F6., U3F7., U3Fy., U3Fz., U3G.., U3G0., U3G1., U3G2., U3G3., U3G4., U3G5., U3G6., U3G7., U3Gy., U3Gz., U3H.., U3H0., U3H1., U3H2., U3H3., U3H4., U3H5., U3H6., U3H7., U3Hy., U3Hz., U3J.., U3J0., U3J1., U3J2., U3J3., U3J4., U3J5., U3J6., U3J7., U3Jy., U3Jz., U3K.., U3K0., U3K1., U3K2., U3K3., U3K4., U3K5., U3K6., U3K7., U3Ky., U3Kz., U3y.., U3y0., U3y1., U3y2., U3y3., U3y4., U3y5., U3y6., U3y7., U3yy., U3yz., U3z.., U3z0., U3z1., U3z2., U3z3., U3z4., U3z5., U3z6., U3z7., U3zy., U3zz., U4..., U43.., U430., U431., U432., U433., U434., U435., U436., U437., U43y., U43z., U44.., U440., U441., U442., U443., U444., U445., U446., U447., U44y., U44z., U45.., U450., U451., U452., U453., U454., U455., U456., U457., U45y., U45z., U46.., U460., U461., U462., U463., U464., U465., U466., U467., U46y., U46z., U47.., U470., U471., U472., U473., U474., U475., U476., U477., U47y., U47z., U48.., U480., U481., U482., U483., U484., U485., U486., U487., U48y., U48z., U49.., U490., U491., U492., U493., U494., U495., U496., U497., U49y., U49z., U4A.., U4A0., U4A1., U4A2., U4A3., U4A4., U4A5., U4A6., U4A7., U4Ay., U4Az., U4B.., U4B0., U4B1., U4B2., U4B3., U4B4., U4B5., U4B6., U4B7., U4By., U4Bz., U4C.., U4C0., U4C1., U4C2., U4C3., U4C4., U4C5., U4C6., U4C7., U4Cy., U4Cz., U4D.., U4D0., U4D1., U4D2., U4D3., U4D4., U4D5., U4D6., U4D7., U4Dy., U4Dz., U4y.., U4y0., U4y1., U4y2., U4y3., U4y4., U4y5., U4y6., U4y7., U4yy., U4yz., U4z.., U4z0., U4z1., U4z2., U4z3., U4z4., U4z5., U4z6., U4z7., U4zy., U4zz., U5..., U50.., U500., U501., U502., U503., U504., U505., U50y., U50z., U51.., U510., U511., U512., U513., U514., U515., U516., U517., U518., U51z. |
