# Supplementary material for: Educational Attainment at Age 10–11 Years Predicts Health Risk Behaviors and Injury Risk During Adolescence
Source: J Adolesc Health. 2017 Aug;61(2):212–8. doi: 10.1016/j.jadohealth.2017.02.003 (PMC5516262; doi:10.1016/j.jadohealth.2017.02.003)
Supplement: Supplement 3 [file mmc3.docx]

Supplement 3: ICD10 injury codes for hospital admissions

|  | **Categories** | **ICD10 codes** |
| --- | --- | --- |
| Injury nature | 1 Significant intracranial injury | S061 - S069 |
|  | 2 Burn | T200 - T329, T330 - T359 |
|  | 3 Poison/Toxic | T360 - T659 |
|  | 4 Long bone fracture | S421 - S427, S480 - S489, S520 - S529, S580 - S589, S720 - S729, S780 - S789, S820 - S829, S880 - S889 |
|  | 5 Other | remaining ICD 10 codes not mentioned above but within S00-S99, T00-T65, T70.4, T70.8, T70.9, T71, T74.1, T75.1, T75.4, T79 |
| External cause | 1 Cut | W25-W299, W45-W459, X78-X789, X99-X999, Y28-Y289, Y354 |
|  | 2 Drowning | W65-W749, X71-X719, X92-X929, Y21-Y219 |
|  | 3 Falls | W00-W199, X80-X809, Y01-Y019, Y30-Y309 |
|  | 4 Fire/hot object or substance | X00-X199, X76-X779, X97-X989, Y26-Y279, Y363 |
|  | 5 Firearm related | W32-W349, X72-X749, X93-X959, Y22-Y249, Y350 |
|  | 6 MVTC | V304-V309, V314-V319, V324-V329, V334-V339, V344-V349, V354-V359, V364-V369, V374-V379, V384-V389, V394-V399, V404-V409, V414-V419, V424-V429, V434-V439, V444-V449, V454-V459, V464-V469, V474-V479, V484-V489, V494-V499, V504-V509, V514-V519, V524-V529, V534-V539, V544-V549, V554-V559, V564-V569, V574-V579, V584-V589, V594-V599, V604-V609, V614-V619, V624-V629, V634-V639, V644-V649, V654-V659, V664-V669, V674-V679, V684-V689, V694-V699, V704-V709, V714-V719, V724-V729, V734-V739, V744-V749, V754-V759, V764-V769, V774-V779, V784-V789, V794-V799, V830-V833, V840-V843, V850-V853, V860-V863, V203-V209, V213-V219, V223-V229, V233-V239, V243-V249, V253-V259, V263-V269, V273-V279, V283-V289, V294-V299, V123-V129, V133-V139, V143-V149, V194-V196, V021-V029, V031-V039, V041-V049, V092, V803-V805, V811, V821, V870-V878, V892 |
|  | 7 Poisoning | X40-X499, X60-X699, X85-X909, Y10-Y199, Y352 |
|  | 8 Struck by/against | W20-W229, W50-W529, X79-X799, Y00-Y009, Y04-Y049, Y29-Y299, Y353 |
|  | 9 Other | remaining ICD 10 codes not mentioned above but within V01-V99, W00-W99, X00-X99, and Y01-Y369 in any diagnostic position |
| Intention of injury | 1 Unintentional | V01-X599 |
|  | 2 Self-harm | X60-X849 |
|  | 3 Assault | X85-Y099 |
|  | 4 Other | remaining ICD 10 codes not mentioned above but within V01-V99, W00-W99, X00-X99, and Y01-Y369 |
