# Supplementary material for: Educational Attainment at Age 10–11 Years Predicts Health Risk Behaviors and Injury Risk During Adolescence
Source: J Adolesc Health. 2017 Aug;61(2):212–8. doi: 10.1016/j.jadohealth.2017.02.003 (PMC5516262; doi:10.1016/j.jadohealth.2017.02.003)
Supplement: Supplement 4 [file mmc4.docx]

Supplement 4: HBSC variables and questions used in the analysis

| **Variable** | **Question number** | **Question** |
| --- | --- | --- |
| Current alcohol consumption | Q27 | At present, how often do you drink anything alcoholic?  (Every day, Every week, Every month, Rarely, Never) |
| Ever been drunk | Q29 | In your lifetime have you ever had so much alcohol that you were really drunk?  (No never, Yes once, Yes 2-3 times, Yes 4-10 times, Yes more than 10 times) |
| Physical activity | M16 | Over a typical or usual week, on how many days are you physically active for a total of at least 60 minutes per day?  (0-7) |
