# Supplementary figures and images for: Educational Attainment at Age 10–11 Years Predicts Health Risk Behaviors and Injury Risk During Adolescence
Source: J Adolesc Health. 2017 Aug;61(2):212–8. doi: 10.1016/j.jadohealth.2017.02.003 (PMC5516262; doi:10.1016/j.jadohealth.2017.02.003)

Supplement 5: Record linkage between the WECC cohort, the education dataset and the HBSC survey


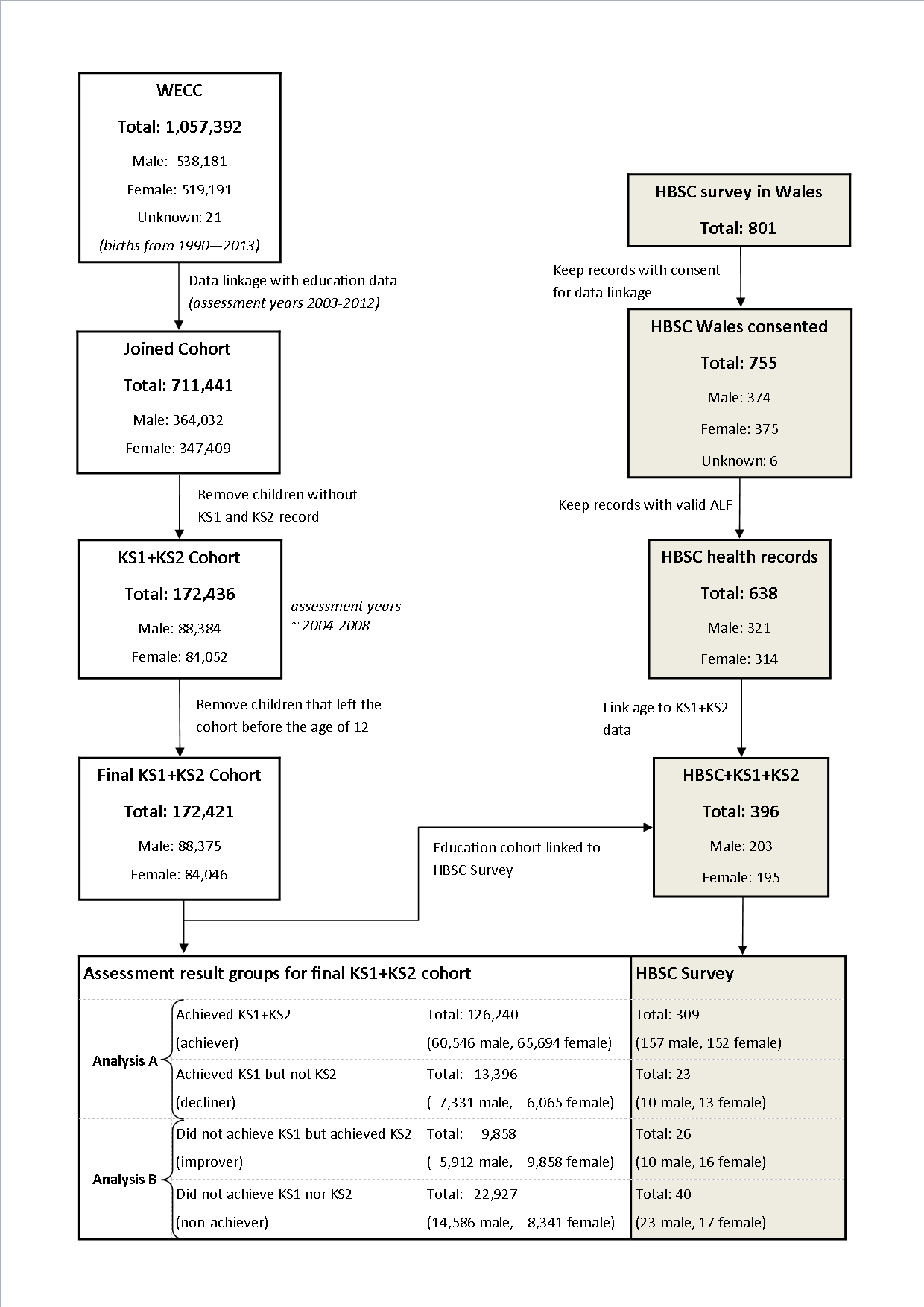

Supplement: Supplement 5 [file mmc5.docx]
