# Supplementary material for: Educational Attainment at Age 10–11 Years Predicts Health Risk Behaviors and Injury Risk During Adolescence
Source: J Adolesc Health. 2017 Aug;61(2):212–8. doi: 10.1016/j.jadohealth.2017.02.003 (PMC5516262; doi:10.1016/j.jadohealth.2017.02.003)
Supplement: Supplement 6 [file mmc6.docx]

Supplement 6: Injury rate. Time to first GP contact for injury by Key Stage achievement and gender. Decliners versus Improvers

|  | **Number of Injuries** | **Follow up years** | **Crude Incidence rate (95%CI)** | **Crude Hazard ratio (95%CI)** | **Hazard ratio adjusted for Free School Meals entitlement (95%CI)** |
| --- | --- | --- | --- | --- | --- |
| **Total** |  |  |  |  |  |
| **Declining** (n= 13,394) | 1,271 | 44,450 | 2.85%  (2.70 to 3.02) |  |  |
| **Improving** (n=9,856) | 729 | 27,103 | 2.68%  (2.49 to 2.88) | 0.90  (0.82 – 0.99)* | 0.90  (0.82 to 0.99)* |
| **Boys** |  |  |  |  |  |
| **Declining** (n=7,330) | 803 | 24,005 | 3.34%  (3.12 to 3.58) |  |  |
| **Improving** (n=6,064) | 518 | 16,072 | 3.21%  (2.95 to 3.50) | 0.92  (0.82 to 1.03) | 0.92  (0.82 to 1.03) |
| **Girls** |  |  |  |  |  |
| **Declining** (n=5,910) | 468 | 20,445 | 2.28%  (2.09 to 2.50) |  |  |
| **Improving** (n=3,946) | 211 | 11,030 | 1.91%  (1.67 to 2.19) | 0.81  (0.69 to 0.95)* | 0.81  (0.69 to 0.95)* |

** Statistically significant*
