# Supplementary material for: Educational Attainment at Age 10–11 Years Predicts Health Risk Behaviors and Injury Risk During Adolescence
Source: J Adolesc Health. 2017 Aug;61(2):212–8. doi: 10.1016/j.jadohealth.2017.02.003 (PMC5516262; doi:10.1016/j.jadohealth.2017.02.003)
Supplement: Supplement 7 [file mmc7.docx]

Supplement 7: Injury rate. Time to first GP contact (group level including multiple injuries per individual) for injury by Key Stage achievement and gender. Decliners versus Improvers

|  | **Number of Injuries** | **Follow up years** | **Crude Incidence rate (95%CI)** | **Crude Hazard ratio (95%CI)** | **Hazard ratio adjusted for Free School Meals entitlement (95%CI)** |
| --- | --- | --- | --- | --- | --- |
| **Total** |  |  |  |  |  |
| **Declining** (n=14,155) | 2,032 | 46,314 | 4.38%  (4.20 to 4.58) |  |  |
| **Improving** (n=10,244) | 1,117 | 28,017 | 3.98%  (3.75 to 4.22) | 0.90  (0.83 to 0.97)* | 0.90  (0.84 to 0.97) |
| **Boys** |  |  |  |  |  |
| **Declining** (n=7,810) | 1,283 | 25,160 | 5.10%  (4.82 to 5.38) |  |  |
| **Improving** (n=6,345) | 814 | 21,153 | 4.48%  (4.52 to 5.19) | 0.94  (0.86 to 1.03) | 0.94  (0.86 to 1.03) |
| **Girls** |  |  |  |  |  |
| **Declining** (n=6,206) | 749 | 16,760 | 3.54%  (3.29 to 3.80) |  |  |
| **Improving** (n=4,038) | 303 | 11,256 | 2.69%  (2.41 to 3.01) | 0.76  (0.66 to 0.86)* | 0.76  (0.66 to 0.87)* |

** Statistically significant*
