# Supplementary material for: Educational Attainment at Age 10–11 Years Predicts Health Risk Behaviors and Injury Risk During Adolescence
Source: J Adolesc Health. 2017 Aug;61(2):212–8. doi: 10.1016/j.jadohealth.2017.02.003 (PMC5516262; doi:10.1016/j.jadohealth.2017.02.003)
Supplement: Supplement 8 [file mmc8.docx]

Supplement 8: Injury rate. Time to first hospital admission for injury by Key Stage achievement and gender. Decliners versus Improvers

|  | **Number of Injuries** | **Follow up years** | **Crude Incidence rate (95%CI)** | **Crude Hazard ratio (95%CI)** | **Hazard ratio adjusted for Free School Meals entitlement (95%CI)** |
| --- | --- | --- | --- | --- | --- |
| **Total** |  |  |  |  |  |
| **Declining** (n=13,396) | 580 | 46,393 | 1.25%  (1.15 to 1.36) |  |  |
| **Improving** (n=9,858) | 328 | 28,051 | 1.17%  (1.05 to 1.30) | 0.92  (0.80 to 1.05) | 0.92  (0.80 to 1.05) |
| **Boys** |  |  |  |  |  |
| **Declining** (n=7,331) | 385 | 25,193 | 1.53%  (1.38 to 1.69) |  |  |
| **Improving** (n=6,065) | 223 | 21,200 | 1.33%  (1.17 to 1.52) | 0.85  (0.72 to 1.00)* | 0.85  (0.72 to 1.00) |
| **Girls** |  |  |  |  |  |
| **Declining** (n=5,912) | 195 | 16,781 | 0.92%  (0.80 to 1.06) |  |  |
| **Improving** (n=3,946) | 105 | 11,270 | 0.93%  (0.77 to 1.13) | 1.01  (0.80 to 1.28) | 1.01  (0.80 to 1.28) |

** Statistically significant*
