# Supplementary material for: Educational Attainment at Age 10–11 Years Predicts Health Risk Behaviors and Injury Risk During Adolescence
Source: J Adolesc Health. 2017 Aug;61(2):212–8. doi: 10.1016/j.jadohealth.2017.02.003 (PMC5516262; doi:10.1016/j.jadohealth.2017.02.003)
Supplement: Supplement 9 [file mmc9.docx]

Supplement 9: Injury rate. Time to hospital admission (group level including multiple injuries per individual) for injury by Key Stage achievement and gender. Decliners versus Improvers

|  | **Number of Injuries** | **Follow up years** | **Crude Incidence rate (95%CI)** | **Crude Hazard ratio (95%CI)** | **Hazard ratio adjusted for Free School Meals entitlement (95%CI)** |
| --- | --- | --- | --- | --- | --- |
| **Total** |  |  |  |  |  |
| **Declining** (n=13,503) | 687 | 46,656 | 1.47%  (1.37 to 1.59) |  |  |
| **Improving** (n=9,897) | 367 | 28,140 | 1.30%  (1.18 to 1.44) | 0.88  (0.77 to 0.99)* | 0.88  (0.77 to 1.00)* |
| **Boys** |  |  |  |  |  |
| **Declining** (n=7,381) | 435 | 25,324 | 1.72%  (1.56 to 1.89) |  |  |
| **Improving** (n=6,122) | 251 | 21,332 | 1.49%  (1.32 to 1.69) | 0.85  (0.73 to 0.99)* | 0.85  (0.73 to 1.00)* |
| **Girls** |  |  |  |  |  |
| **Declining** (n=5,940) | 252 | 16,839 | 1.18%  (1.04 to 1.34) |  |  |
| **Improving** (n=3,957) | 116 | 11,300 | 1.03%  (0.86 to 1.23) | 0.87  (0.70 to 1.09) | 0.87  (0.70 to -1.09) |

** Statistically significant*
